# Tree canopy arthropods have idiosyncratic responses to plant ecophysiological traits in a warm temperate forest complex

**DOI:** 10.1038/s41598-020-76868-8

**Published:** 2020-11-16

**Authors:** Rudi C. Swart, Michael J. Samways, Francois Roets

**Affiliations:** Department of Conservation Ecology and Entomology, Stellenbosch University, Private Bag X1, Matieland, 7602 South Africa

**Keywords:** Ecology, Biodiversity, Ecosystem ecology, Forest ecology

## Abstract

Biodiversity studies on forest canopies often have narrow arthropod taxonomic focus, or refer to a single species of tree. In response, and to better understand the wide range of drivers of arthropod diversity in tree canopies, we conducted a large-scale, multi-taxon study which (a) included effect of immediate surroundings of an individual tree on plant physiological features, and (b), how these features affect compositional and functional arthropod diversity, in a warm, southern Afro-temperate forest. We found that tree species differed significantly in plant physiological features and arthropod diversity patterns. Surprisingly, we found negative correlation between surrounding canopy cover, and both foliar carbon and arthropod diversity in host trees, regardless of tree species. Subtle, tree intraspecific variation in physiological features correlated significantly with arthropod diversity measures, but direction and strength of correlations differed among tree species. These findings illustrate great complexity in how canopy arthropods respond to specific tree species, to immediate surroundings of host trees, and to tree physiological features. We conclude that in natural forests, loss of even one tree species, as well as homogenization of the crown layer and/or human-induced environmental change, could lead to profound and unpredictable canopy arthropod biodiversity responses, threatening forest integrity.

## Introduction

Trees and their associated arthropods contribute greatly to terrestrial biodiversity, though ecologists only recently have begun to decipher the complexities of their interactions^1^. This progress has been possible as arthropods in tree canopies are now more accessible, making way for greater insights into the complexities of tree-arthropod interactions, including canopy arthropod feeding guild partitioning^2^, incorporation of evolutionary histories of host tree species^3^, and effects of host-tree genetic diversity on arthropod diversity^4^. However, how arthropod diversity in these little-known yet important elevated communities respond to subtle changes in plant physiological features, many of which are human-induced, remains poorly studied. Also, due to the large numbers of arthropod species supported at canopy level, many studies often focus on one arthropod taxonomic group^2,5^ or on a single tree species^6,7^. As the multi-taxon approach provides deeper insight into ecological patterns and processes^8^, much of our earlier understanding regarding tree-arthropod interactions only applies in narrow contexts. A larger scale, multi-taxon approach is required to understand the drivers of arthropod diversity in forest canopies.

Host specificity of arthropods in tree canopies is lower than previously suggested^9^. Nevertheless, host tree species identity is an important driver of arthropod diversity in forest canopies, determined largely by differences in plant physiological features such as leaf size and shape, chemical defences, turgor, and nitrogen content^10^. The impact of the immediate surroundings of a tree individual on arthropod diversity, and tree physiology, remains poorly studied, yet important^11^. When surrounded by dense canopies, a focal tree individual receives less direct sunlight, which decreases its photosynthetic capabilities and alters its nutritional value^12^. Changes in available nutrients also affect plant physiological features^13^. These various changes influence arthropod diversity at tree canopy level, yet are poorly understood^14^.

Effect of climate change on the interaction between trees and associated arthropods also requires consideration, especially the extent to which tree responses to changing conditions might spill over to higher trophic levels^15^. Our poor understanding of these interactions is concerning given that even a 1ºC increase in global temperature may have profound effects on the interactions between trees and arthropods^16^. Decreased rainfall from a changing climate can increase water stress, reduce growth, and so disrupt plant-arthropod dynamics, but can also increase forest vigour and growth, and lead to higher water use efficiency and extended growing seasons^16,17^.

Precisely how changes in environmental factors influence plant physiological features and how arthropods respond to these changes is difficult to predict, as there may be differences between plant species, as well as between arthropod taxa. For example, some generalist herbivores benefit from severe drought conditions, while certain specialist herbivores benefit from moderate drought levels^15^. This means that when incorporating tree physiological features in canopy-arthropod studies, multiple tree species and arthropod guilds are required. Such studies can provide new insight into how future climate change will shape tree canopy-arthropod interactions, as it does for plant-pollinator interactions^18^.

Analysis of leaf physiological features might provide information on tree nutrient status and investment in defence compounds, while contributing to assessment of tree stress, such as moisture deficiency. Foliar N concentration, in particular, is an important determinant of herbivore arthropod diversity, distribution, and feeding behaviour^19^. Conversely, increased levels of foliar C often indicate plant investment in anti-herbivory structural defence compounds, phenols, and tannins^20^. Consequently, relative increases in foliar C/N ratio often indicate more allocation to carbon-based defences against herbivores, with still-contested effects on higher trophic levels^21^. Plant investment in production of these compounds is determined by level of light exposure^21^, which can fluctuate with subtle changes in moisture availability and temperature^22^. Drought-stress, for example, can increase relative metabolic uptake of δ13C^23^. In turn, variations in δ15N indicate differences in N sources, for example, whether derived from the soil or atmosphere, while also indicating plant stress or exposure to pollution^24^. Response of canopy-associated arthropod assemblages to changes in N and C isotopes can therefore provide valuable information on their responses to various stressors as predicted under future climate change.

Few studies have evaluated canopy arthropod diversity patterns while including surrounding plot characteristics, with some seminal work including canopy density effects^25^ and tree immediate surrounds^11^. However, we are unaware of any work on how plot characteristics affect physiological features among a mix of different tree species in an indigenous forest system, and in turn, affect tree-associated arthropod diversity. In this novel study, we ask: (1) How do tree identity and plot characteristics affect tree physiological features, and arthropod diversity within tree canopies, and (2) How does canopy arthropod diversity change in response to intraspecific variations in tree physiological features. We therefore focus on the importance of tree surrounds, and use proxies for tree drought stress, carbon-based defences and differences in nutrient resources, to describe the diversity and distribution of canopy arthropod diversity.

## Results

In total, 20,645 arthropod individuals were sampled, comprising 1512 species (Supplementary table S1 online). Estimates of species richness of 2569.5 (± 109.98) and 2679.2 were obtained for Chao2 and Jacknife2 indexes respectively, indicating that we sampled just over 58% of estimated species richness (Supplementary Fig. S1 online). Predators were the most species-rich guild, totalling 768 species and 51.79% of all sampled species, followed by herbivores (321 spp.), detritivores (207 spp.), tourists (129 spp.), and ants (36 spp.). Predators were also the most abundant guild, with 7549 individuals, 36.57% of all sampled individuals. Herbivores comprised 5538 of individuals, 26.82% of all catches. These were followed by detritivores (4366 individuals = 21.15% of all catches), ants (1175 individuals = 5.69% of all catches) and tourists (1153 individuals = 5.58% of all catches).

### Effect of tree identity and plot characteristics on tree physiological features

Except for foliar C, which was negatively correlated with plot cover, and foliar δ15N/14N increasing with focal tree cover, all plant physiological features were influenced only by tree identity (Table 1). Foliar N was the highest in *O. ventosa* (Supplementary Fig. S2 online). There was large variance around medians for δ15N/14N between tree species, with few significant differences detected (Supplementary Fig. S3 online). Significantly higher leaf C was only detected in *C. dentata* (Supplementary Fig. S4 online), with *C. capensis* having lowest levels of foliar C. Three tree species, *C. capensis*, *O. ventosa* and *P. latifolius*, had significantly higher levels of foliar δ13C/12C compared to *O. c. macrocarpa*, *P. tricuspidatus, P. trifoliatus* and *R. melanophloeos* (Supplementary Fig. S5 online). *Curtisia dentata* showed intermediate levels of δ13C/12C, and *O. ventosa* had the lowest C/N ratio (Supplementary Fig. S6 online).

**Table 1:** Results of the linear mixed models indicating F-values for each of the model variables for the respective plant physiological variables, including the significance for each variable.

Physiological variable	Variable	num. df	F	Pr (> F)	Sig
N	Focal tree species	7	21.38	0.000	***
	Focal tree cover	1	0.43	0.51	ns
	Host same-species cover	1	0.07	0.79	ns
	Plot richness	1	0.15	0.70	ns
	Plot cover	1	1.33	0.25	ns
δ15N/14N	Focal tree species	7	6.08	0.000	***
	Focal tree cover	1	7.05	0.009	**
	Host same-species cover	1	0.50	0.48	ns
	Plot richness	1	0.12	0.73	ns
	Plot cover	1	2.24	0.14	ns
C	Focal tree species	7	37.06	0.000	***
	Focal tree cover	1	1.99	0.16	ns
	Host same-species cover	1	0.75	0.39	ns
	Plot richness	1	0.45	0.51	ns
	Plot cover	1	5.48	0.02	*
δ13C/12C	Focal tree species	7	13.47	0.000	***
	Focal tree cover	1	0.33	0.57	ns
	Host same-species cover	1	0.00	0.97	ns
	Plot richness	1	1.24	0.27	ns
	Plot cover	1	2.20	0.14	ns
C/N	Focal tree species	7	27.42	0.000	***
	Focal tree cover	1	0.05	0.83	ns
	Host same-species cover	1	0.33	0.57	ns
	Plot richness	1	0.83	0.36	ns
	Plot cover	1	1.73	0.19	ns

‘.’ *P* < 0.01, ‘*’ *P* < 0.05, ‘**’* P* < 0.01, ‘***’* P* < 0.001.

### Effect of tree identity and plot characteristics on canopy arthropod diversity

Arthropod species density (richness per standardized sampling area) varied between a mean of 48.8 (± 5.09 SE) and 79.27 (± 8.92 SE), and abundance between 122.33 (± 19.46 SE) and 212.27 (± 29.82 SE) for the different tree species (Table 2). Focal tree species was an important explanatory variable for differences in arthropod abundance for all groups except the predators and ants (Table 3). However, there were no significant differences between particular tree species after post-hoc analyses for overall arthropods or herbivores. Detritivores were most abundant in canopies of *C. capensis*, *C. dentata, O. c. macrocarpa, O. ventosa* and *P. latifolius*, and least abundant in the canopy of *P. trifoliatus* (Supplementary Fig. S7 online), whereas more tourists were sampled from the canopy of *P. trifoliatus* compared to *P. tricuspidatus* (Supplementary Fig. S8 online). Plot canopy cover was the second most important factor explaining abundance of sampled arthropods on focal trees, with plot cover negatively correlating with abundances of overall arthropods, herbivores, predators, detritivores and tourists (Table 3). Ant abundance was positively correlated to tree species richness (Table 3).

**Table 2:** Summary statistics of abundance and species density (mean ± s.e.m.) sampled per individual tree from each of the respective tree species (n = 15) for the respective canopy arthropod guilds.

Diversity indices	*C. capensis*	*C. dentata*	*O. c. macrocarpa*	*O. ventosa*	*P. latifolius*	*P. tricuspidatus*	*P. trifoliatus*	*R. melanophloeos*
N	188.2 ± 29.11	212.27 ± 29.82	169.47 ± 15.06	203.67 ± 28.91	163.8 ± 26.33	146 ± 25.64	125.87 ± 23.87	122.33 ± 19.46
S	66.73 ± 7.68	79.27 ± 8.92	64.53 ± 4.26	69.13 ± 7.67	56.93 ± 4.75	58.67 ± 6.92	48.8 ± 5.09	55.2 ± 7.24
Herbivore N	46.53 ± 8.45	53.07 ± 10.59	52.93 ± 7.15	55.93 ± 12.15	58.47 ± 13.67	35.27 ± 7.22	35.87 ± 10.45	31.13 ± 5.19
Herbivore S	13.2 ± 1.52	14.73 ± 2.05	13.87 ± 1.21	13.33 ± 1.6	13.73 ± 1.36	12.47 ± 1.59	10.67 ± 1.11	11.53 ± 1.25
Predator N	68.8 ± 13.76	84.4 ± 12.22	56.53 ± 6.2	78.79 ± 13.829	65 ± 8.14	54 ± 10.44	43.67 ± 8.43	52 ± 11.40
Predator S	30.53 ± 4.31	39.4 ± 5.08	30 ± 2.84	35.2 ± 4.62	31.3 ± 2.82	27.27 ± 4.01	21.13 ± 2.99	29.87 ± 4.68
Detritivore N	44.07 ± 7.43	42.4 ± 6.55	39.2 ± 5.26	40.27 ± 6.04	41.27 ± 5.21	27.2 ± 3.38	21.93 ± 3.64	34.73 ± 5.38
Detritivore S	12.8 ± 1.29	14.67 ± 1.49	13.07 ± 0.84	12.67 ± 1.33	12.47 ± 1.11	11.53 ± 1.25	7.93 ± 0.97	12.27 ± 1.46
Tourist N	12.27 ± 3.77	11.73 ± 2.16	6.47 ± 2.07	5.27 ± 0.9	10.13 ± 3.31	5.67 ± 0.98	16.8 ± 6.26	7.53 ± 1.42
Tourist S	5.2 ± 0.9	6.53 ± 1.05	3.6 ± 0.71	3.73 ± 0.51	5.07 ± 1.09	3 ± 0.38	5.53 ± 0.97	4 ± 0.67
Ant N	8.73 ± 2.41	10.53 ± 3.29	6.87 ± 1.63	16.13 ± 5.13	7.87 ± 4.33	15.73 ± 6.31	4.53 ± 1	8.13 ± 4.89
Ant S	2.47 ± 0.36	1.53 ± 0.24	2.07 ± 0.33	2.73 ± 0.44	1.93 ± 0.36	2.73 ± 0.57	1.67 ± 0.33	1.47 ± 0.34

N = Number of specimens (abundance); S = Species density.

**Table 3:** Results of the Generalized linear mixed modelling indicating chi-square values of each of the model variables for the respective arthropod guilds for abundance and species density data, including significance for each model variable.

Guild	Variable	Abundance				Species density			
		df	Chi-square	Pr (> Chisq)	Sig	df	Chi-square	Pr (> Chisq)	Sig
Overall	Focal tree species	7	14.41	0.04	*	7	157.61	< 0.001	***
	Focal tree cover	1	1.31	0.25	ns	1	20.72	< 0.001	***
	Host same-species cover	1	0.50	0.48	ns	1	11.23	< 0.001	***
	Plot richness	1	1.09	0.30	ns	1	10.12	< 0.01	**
	Plot cover	1	(-)11.96	< 0.001	***	1	(-)99.45	< 0.001	***
Herbivore	Focal tree species	7	14.59	0.04	*	7	21.37	< 0.01	**
	Focal tree cover	1	0.32	0.57	ns	1	2.00	0.157	ns
	Host same-species cover	1	2.22	0.14	ns	1	3.28	0.07	ns
	Plot richness	1	0.26	0.61	ns	1	0.16	0.69	ns
	Plot cover	1	(-)10.69	0.001	**	1	(-)28.24	< 0.001	***
Predator	Focal tree species	7	13.65	0.06	ns	7	118.2	< 0.001	***
	Focal tree cover	1	1.3	0.25	ns	1	15.6	< 0.001	***
	Host same-species cover	1	0.01	0.93	ns	1	4.19	< 0.05	*
	Plot richness	1	0.5	0.48	ns	1	13.53	< 0.001	***
	Plot cover	1	(-)6.53	0.01	*	1	(-)51.5	< 0.001	***
Detritivore	Focal tree species	7	24.82	< 0.001	***	7	38.01	< 0.001	***
	Focal tree cover	1	3.11	0.08	ns	1	2.65	0.01	ns
	Host same-species cover	1	(-)0.65	0.42	ns	1	0.64	0.43	ns
	Plot richness	1	1.01	0.32	ns	1	(-)0.17	0.68	ns
	Plot cover	1	(-)6.62	0.01	*	1	(-)9.3	< 0.01	**
Tourists	Focal tree species	7	19.55	< 0.01	**	7	36.18	< 0.001	***
	Focal tree cover	1	0.06	0.82	ns	1	0.32	0.57	ns
	Host same-species cover	1	0.45	0.50	ns	1	1.29	0.25	ns
	Plot richness	1	(-)0.04	0.85	ns	1	1.43	0.23	ns
	Plot cover	1	(-)7.97	< 0.01	**	1	(-)9.57	< 0.01	**
Ants	Focal tree species	7	13.34	0.06	ns	7	12.83	0.08	ns
	Focal tree cover	1	0.05	0.82	ns	1	3.82	0.05	ns
	Host same-species cover	1	1.33	0.25	ns	1	2.29	0.13	ns
	Plot richness	1	7.29	< 0.01	**	1	1.36	0.24	ns
	Plot cover	1	(-)0.90	0.32	ns	1	(-)2.01	0.16	ns

‘.’ *P* < 0.01, ‘*’ *P* < 0.05, ‘**’* P* < 0.01, ‘***’* P* < 0.001.

(-) represents a negative correlation.

Species density of overall arthropods, herbivores, predators, detritivores and tourists were significantly different between different tree species (Table 3). Highest overall and predator species density was in canopies of *C. dentata* (Supplementary Figs. S9-10 online). Also, *C. dentata* hosted significantly more herbivore species than *P. trifoliatus* and *R. melanophloeos* (Supplementary Fig. S11 online), and had comparatively high numbers of detritivores and tourists (Supplementary Figs. S12-S13 online). Species density of all guilds negatively correlated with plot canopy cover, except for ants, which were not affected by any of the variables here. Species density of predators positively correlated with focal tree cover, host same-species cover and an increase in tree species per plot, with overall patterns seemingly driven by predator responses (Table 3).

**Figure 1:**
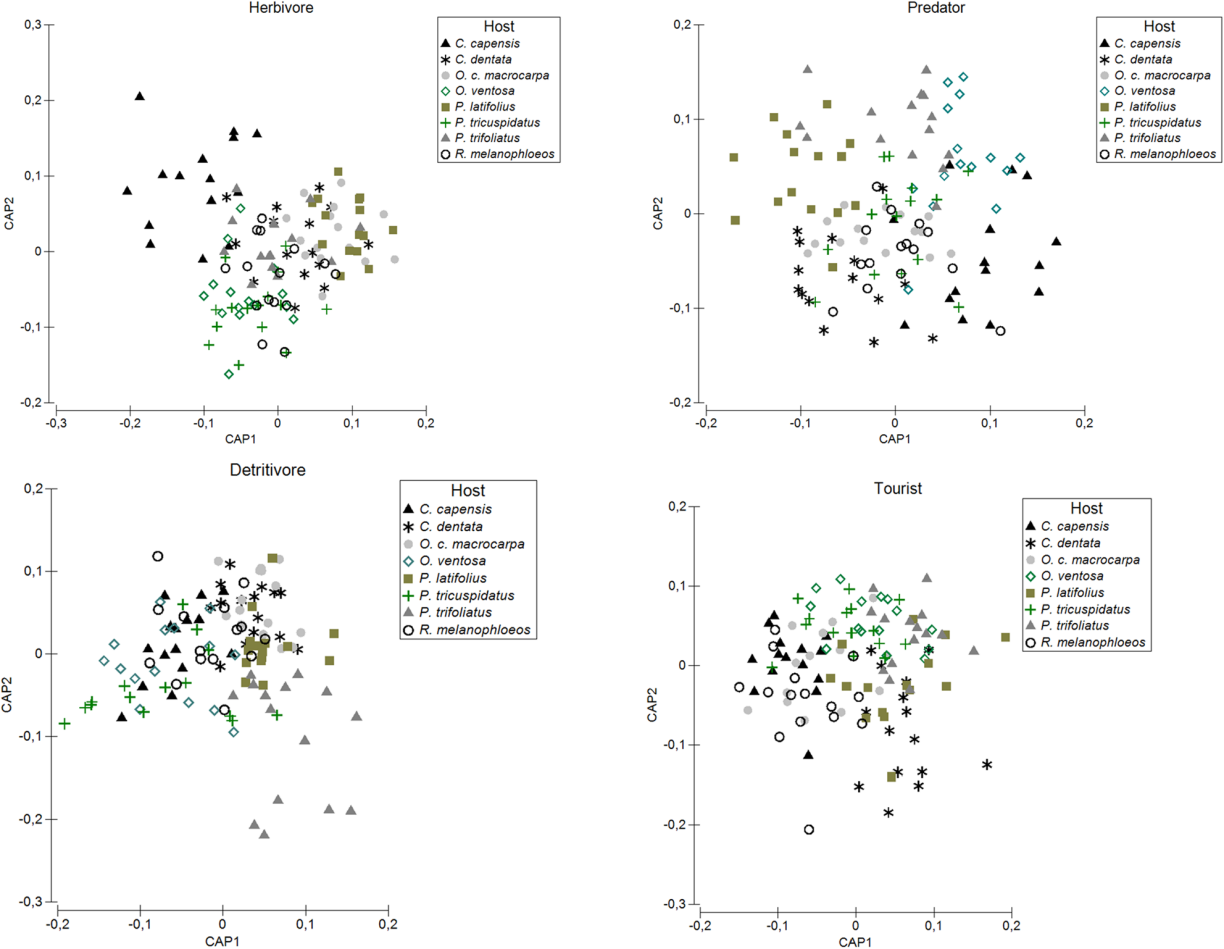
Visualization of assemblage composition results for the herbivore, predator, detritivore and tourist guilds using Canonical Analysis of Principal coordinates (CAP) between the eight tree species based on Bray–Curtis dissimilarities.

Similar to the other arthropod diversity measures, assemblage composition differed significantly for focal tree species identity, with all guilds, except the ants, revealing differences (Supplementary table S2 online). Tree species with the most dissimilar overall assemblage composition was *C. dentata*. Herbivores from *C. capensis* were most divergent in terms of their assemblage composition from those collected from other host species, with herbivore assemblages from *R. melanophloeos* overlapping considerably with those from other hosts (Fig. 1). Predator assemblage composition in canopies of *C. dentata* and *O. ventosa* differed substantially from those in canopies of other host tree species, and detritivore assemblages in canopies of *P. trifoliatus* was significantly different than those in the canopies of most other tree species (Fig. 1). Tourist assemblage composition showed the smallest response to different host tree species (Supplementary table S2 online; Fig. 1). Focal tree canopy cover affected only ant assemblage composition. Host same-species cover within a plot significantly explained overall, herbivore, predator and ant assemblage composition on focal trees (Table 4). Plot tree species richness explained variation only for herbivore assemblage composition. Assemblage composition of all arthropod guilds was significantly explained by total plot canopy cover.

**Table 4:** Results of the distance-based linear modelling sequential tests indicating the significance of the selected variables on assemblage composition of different arthropod guilds from the canopies, regardless of species.

Variable	Statistic	Overall	Herbivores	Predators	Detritivores	Tourists	Ants
Focal tree cover	AICc	972.23	959.06	974.1	945.89	956.19	931.17
	SS	4136.5	4293.3	3202.7	3636.9	2918.3	6094.9
	Pseudo-F	1.28	1.48	0.97	1.40	1.03	2.65**
	Prop. Variance	0.01	0.01	0.01	0.01	0.01	0.02
	Cum. Variance	0.01	0.01	0.01	0.01	0.01	0.02
Host same-species cover	AICc	972.62	959.09	975.25	946.77	956.95	931.29
	SS	5411.9	5881.5	5045.3	3125.9	3743.4	4456.7
	Pseudo-F	1.68**	2.04**	1.53**	1.2	1.32	1.95*
	Prop. Variance	0.01	0.02	0.01	0.01	0.01	0.02
	Cum. Variance	0.02	0.03	0.02	0.02	0.02	0.04
Plot richness	AICc	973.58	959.4	976.4	947.69	958.51	932.44
	SS	3684.1	5110.3	3200.5	3075.7	1600.9	2203.3
	Pseudo-F	1.14	1.79*	0.97	1.18	0.56	0.96
	Prop. Variance	0.01	0.01	0.01	0.01	0.00	0.01
	Cum. Variance	0.03	0.04	0.03	0.03	0.02	0.05
Plot cover	AICc	973.87	959.51	977.1	947.96	958.39	932.6
	SS	5843	5656.7	4685.2	4739.4	6246.6	4407.7
	Pseudo-F	1.83***	1.99**	1.43*	1.84*	2.22**	1.94*
	Prop. Variance	0.02	0.02	0.01	0.02	0.02	0.01
	Cum. Variance	0.05	0.06	0.04	0.05	0.04	0.06

‘*’ *P* < 0.05, ‘**’* P* < 0.01, ‘***’* P* < 0.001.

### Effect of intraspecific physiological variation on arthropod diversity

Overall arthropod abundance was significantly positively correlated with foliar N, but negatively correlated with foliar C for *C. capensis* and *O. ventosa* (Table 5). The converse was the case for association between overall arthropods collected from *P. tricuspidatus* canopies, in which overall arthropod abundances negatively correlated with foliar N, and positively to foliar C. Overall arthropod abundance significantly correlated with foliar δ13C/12C in all tree canopies. However, in certain host species these correlations were positive, while in others negative. Among herbivores, abundance positively correlated with foliar N but negatively with foliar C in some host species (*C. capensis*, *O. c macrocarpa* and *O. ventosa*), and a converse pattern in others (*P. tricuspidatus* and *P. trifoliatus*) (Table 5). Herbivore abundances significantly correlated with foliar total C in all tree canopies, except *C. dentata*. These responses ranged from being either positive or negative, depending on identity of host species. Herbivore abundance correlated positively (*C. capensis*, *O. c. macrocarpa* and *O. ventosa*), negatively (*P. tricuspidatus*, *P. trifoliatus* and *R. melanophloeos*) or neutral (*C. dentata* and *P. latifolius*) towards changes in C/N ratio. Ants were the guild most responsive to foliar δ15N/14N, with five tree species showing significant correlations. However, these correlations were either positive or negative, again depending on host tree species (Table 5).

**Table 5:** Results of the model selection procedure (based on second order Akaike Information Criterion) indicating correlations of measured plant characteristics on canopy arthropod abundances for each of the respective arthropod guilds among eight tree species. Reported z-values.

Guild	Variable	*C. capensis*	*C. dentata*	*O. c. macrocarpa*	*O. ventosa*	*P. latifolius*	*P. tricuspidatus*	*P. trifoliatus*	*R. melanophloeos*
Overall	N	5.58***		− 2.95**	2.95**		− 6.59***	− 5.07***	
	δ15N/14N	3.04**	− 3.63***	− 2.84**			5.76***		− 16.54***
	C	− 8.10***	− 4.67***		− 6.64***	4.08***	4.96***		
	δ13C/12C	− 5.47***	2.59**	− 4.49***	5.21***	10.95***	− 5.54***	14.00***	3.60***
	C/N	5.27***		− 6.36***	3.52***	− 9.64***	− 9.12***	− 4.51***	
Herbivores	N	5.17***	− 6.47***	5.57***	2.76**		− 3.76***	− 3.44***	
	δ15N/14N		− 3.26**	− 3.29***		3.44***			
	C	− 6.93***		− 6.50***	− 5.01***	7.50***	8.70***	4.21***	− 2.60**
	δ13C/12C	− 5.77***	8.97***		− 6.02***	4.19***	− 7.75***	8.36***	
	C/N	4.79***		5.48***	4.00***		− 5.19***	− 3.39***	− 5.33***
Predators	N		3.97***	2.72**			− 7.71***		
	δ15N/14N						10.43***	− 4.56***	− 10.55***
	C	− 5.27***	− 5.12***		− 4.41***			− 4.84***	
	δ13C/12C	− 6.57***		− 4.66***	6.92***	5.91***		6.83***	
	C/N	− 4.50***	3.74***			− 7.87***	− 8.60***		
Detritivores	N			− 5.28***	3.75***	3.56***			
	δ15N/14N		− 3.02**						− 6.50***
	C	− 4.65***		4.88***	− 3.41***	− 4.41***		− 2.21*	
	δ13C/12C	4.07***	− 2.85**	− 2.50*	3.66***	4.01***		4.41***	
	C/N			− 5.58***	3.76***		− 2.88***		
Tourists	N		3.56***			3.29**			
	δ15N/14N	3.69***							− 4.11***
	C		− 4.15***	− 3.44***		2.59**		− 2.72**	
	δ13C/12C	− 5.73***	2.75**			5.30***		7.50***	
	C/N		3.72***	− 5.07***		2.98**		5.06***	
Ants	N	− 5.00***	2.01*			− 6.07***			
	δ15N/14N	2.45*		− 3.43***	3.97***	− 3.41***			− 2.71**
	C			− 3.19**	− 5.23***		7.83***		− 1.97*
	δ13C/12C	− 2.88**	− 4.79***		7.84***	3.00**	− 7.99***		3.69***
	C/N					− 6.15***	− 5.01***		

‘*’ *P* < 0.05, ‘**’* P* < 0.01, ‘***’* P* < 0.001.

Overall species density both positively and negatively correlated with foliar N, depending on host tree species (Table 6). Herbivore species density positively correlated with foliar N in only one host species, *P. latifolius*, and was negatively correlated to foliar C, but in *C. capensis* canopies only. Predators showed mixed patterns in response to variation in foliar N, δ15N/14N, δ13C/12C and C/N. For example, predator species density was positively correlated with δ13C/12C in canopies of *O. ventosa*, *P. trifoliatus* and *P. latifolius*, whereas a negative correlation in canopies of *O. c. marcocarpa* was found (Table 6).

**Table 6:** Results of the model selection procedure (based on second order Akaike Information Criterion) indicating the effects of measured plant characteristics on canopy arthropod species density for each of respective arthropod guilds among eight tree species. Reported z-values.

Guild	Variable	*C. capensis*	*C. dentata*	*O. c. macrocarpa*	*O. ventosa*	*P. latifolius*	*P. tricuspidatus*	*P. trifoliatus*	*R. melanophloeos*
Overall	N	3.57***	− 2.52*			4.46***	− 4.29***		
	δ15N/14N		− 4.26***				5.64***	− 2.67**	− 3.69***
	C	− 5.85***						− 3.11**	
	δ13C/12C	− 2.50*			2.27*	4.89***		5.39***	
	C/N			− 2.63**	3.89***		− 4.71***		− 1.97*
Herbivores	N					2.95**			
	δ15N/14N			− 2.36*					
	C	− 2.23*							
	C/N			− 2.20*					− 2.27*
Predators	N	2.41*					− 3.27**	− 2.50*	
	δ15N/14N		− 2.88**				4.20***		− 6.19***
	C	− 5.63***				2.32*			
	δ13C/12C			− 2.09*	2.35*	3.17**		3.53***	
	C/N			− 2.30*	3.08**	− 2.71**	− 3.68***		
Detritivores	δ15N/14N		− 3.46***						− 3.71***
	C	− 2.03*							
	C/N		2.04*		2.73*				
Tourists	δ15N/14N			3.05**					
	C					2.13*			
	δ13C/12C	− 2.28*				2.53*	2.28*		
Ants	δ15N/14N								− 2.55**

‘*’ *P* < 0.05, ‘**’* P* < 0.01, ‘***’* P* < 0.001.

Intraspecific responses revealed the most important physiological variable explaining variation in arthropod assemblage composition was δ15N/14N, with all eight species showing significance for at least one guild (Table 7). For four tree species, ant assemblage composition was best explained by the variable δ15N/14N. In *C. dentata*, *P. latifolius, P. tricuspidatus* and *R. melanophloeos* canopies, herbivore assemblage composition was best explained by δ15N/14N. Changes in δ13C/12C was associated with changes in assemblage composition of overall arthropod, herbivore, detritivore and predator guilds, although on only one host, *P. tricuspidatus* (Table 7).

**Table 7:** Results of distance-based linear modelling (DistLM) sequential tests, indicating most descriptive plant physiological variable/s for each selected canopy arthropod guild assemblage composition among selected tree species.

Tree species	Guild	Variable	Pseudo-F	Variation explained (%)
*C. capensis*	Predators	C	1.62*	10.45
	Detritivores	δ15N/14N	1.73*	12.01
	Ants	δ15N/14N	2.32*	14.97
*C. dentata*	Herbivores	δ15N/14N	1.86*	12.86
*O. c. macrocarpa*	Predators	C	1.64*	10.82
	Ants	δ15N/14N	2.28*	15.30
*O. ventosa*	Overall	δ15N/14N	1.55*	10.55
	Predators	N	1.42*	9.83
	Ants	δ15N/14N	3.77**	22.24
*P. latifolius*	Herbivores	δ15N/14N	1.77*	11.85
*P. tricuspidatus*	Overall	δ15N/14N	1.56*	10.73
		δ13C/12C	2.10**	13.22
	Herbivores	δ15N/14N	1.73*	11.86
		δ13C/12C	1.83*	11.72
	Predators	δ13C/12C	1.88*	12.27
	Detritivores	δ13C/12C	2.55**	15.90
	Tourists	N	2.79**	17.69
	Ants	δ15N/14N	2.63*	16.78
*P. trifoliatus*	Tourists	δ15N/14N	2.24*	14.98
*R. melanophloeos*	Overall	δ15N/14N	1.65*	11.36
	Herbivores	δ15N/14N	1.87*	12.66
	Detritivores	δ15N/14N	1.88*	12.74
	Tourists	δ15N/14N	2.45**	15.36

‘.’ *P* < 0.01, ‘*’ *P* < 0.05, ‘**’* P* < 0.01, ‘***’* P* < 0.001.

## Discussion

Diversity and distribution of canopy arthropods in an African warm temperate forest are remarkably intricate, with each tree species playing a central role in determining their diversity (density, abundance) and assemblage composition. Moreover, our results indicate that, underlying the role of different tree species in dictating patterns in diverse, higher trophic levels, are the physiology of a tree individual, its immediate surrounds within diverse forests, and the interactions between tree physiology and immediate surrounds. Based on this, predictions regarding canopy arthropod responses to future change appears difficult to confidently make.

Here, most tree species hosted unique arthropod assemblages, with the herbivores and the predators showing the most dissimilarity between tree species. In agreement with most global literature, in which herbivorous arthropods are understood to be at least as specialized as pollinators^9^, these dissimilarities could be driven by host specificity. Host specificity among herbivores arises from interspecific differences in tree morphology, physiology, and phenology, with adaptations to these becoming increasingly host species-specific over time^10,26^. From the assemblage composition analyses, and similar to previous work^27^, we suggest that arthropod specialization towards different tree species does not seem to be restricted only to folivores, but might also shift beyond lower trophic levels to include predatory arthropods^28^. Also, the assemblages of herbivores and predators, along with the ants, but not detritivores or tourists, were strongly explained by host same-species cover (the canopy cover of tree species similar to focal tree species within a plot). This might further indicate host specificity, relating to the resource concentration hypothesis: that the distribution of species will reflect the density of its preferred resources, such as herbivores on host trees^29^. Subsequently, many arthropod guilds can be considered host specific, albeit secondarily, highlighting the importance of retaining different tree species in the conservation of higher trophic levels. Losing a single tree species from these scattered forest patches could cause the local extirpation of unique assemblages, which will include numerous cryptic and undescribed species.

Contrary to resource concentration hypotheses, and increases in microhabitats with denser foliage^25^, we found a strong negative correlation between plot cover and arthropod diversity on host trees. This is similar to Finnish forests, where gall abundance on spruce trees decreased with increased cover^30^. Decreased arthropod diversity associated with increased canopy cover is often related to decreases in light exposure^21^. We show that foliar C concentration in leaves was lowest where light exposure was least (re: high plot cover), a finding supported elsewhere, with reductions in carbon-based phenol concentrations in less light-exposed leaves^31,32^. Interestingly, foliar C, as well as arthropod diversity, increased on focal trees with less surrounding canopy cover. In line with these findings, increased carbon-based defences and greater herbivore damage occurred among tree seedlings in tree-fall gaps compared to forest interiors in a temperate Chilean rainforest^20^. Therefore, despite increases in carbon-based defences in more light exposed plants, herbivore diversity does not necessarily respond negatively. This suggests that arthropod diversity is strongly driven by canopy structural heterogeneity, creating numerous microhabitats, ranging from highly exposed to light to completely shaded^33^. Our results concur with this, and show all arthropod guilds’ assemblage compositions were significantly explained by variations in plot cover. More directly, light exposure leads to increased temperatures that have positive effects on development of numerous arthropod taxa^34^, while homogenization of forests, through decreases in both structural diversity and tree richness, can reduce arthropod diversity^35^. This is concerning, with losses of the natural heterogeneity of unmanaged, old-growth forests predictably negatively impacting optimal ecosystem-level conservation, while plantation forests and large-scale homogenization are increasing worldwide^36^.

With the exception of total plot canopy cover, few other plot-scale characteristics influenced arthropod abundance and density of focal trees. However, it is interesting that predator species density was most affected by plot-scale characteristics, with density increasing with increased tree species richness around the focal tree. This supports the enemies hypothesis, which predicts increases in predator diversity in more diverse plant communities^37^. For example, an increase in localized tree richness will have a direct effect on the number of micro-habitats, and provide more variation in prey base and temporal stability in prey availability^37^. Interestingly, plot tree richness explained variation in the assemblages of only one guild, the herbivores. The relationship between plant richness, herbivore assemblages and predator diversity was not specifically tested here, and future work could shed light on these interesting patterns.

Whereas species density of especially the predators, and little else, seemed to be driven by plot-scale variables, ecophysiological variables strongly correlated with the abundances more than with densities of various guilds. Here, we show correlations between intraspecific variability of leaf physiological traits and abundances of all arthropod guilds. Foliar resource availability and its effect on higher trophic levels, although widely studied, is poorly understood. Differences in physiological traits can be due to host genetics^38^, but often also due to differences in nutrient or moisture availability^22^, expected to increase fluctuations beyond species’ thresholds under global climate change^39^. Differences in N between trees, for example, could greatly affect eventual ecosystem processes, especially nutrient cycling, due to N investment in metabolic compounds^40^, with N concentration often but not exclusively an indication of leaf quality for insect herbivores^19^. Foliar N here had differential impacts on arthropods, exhibiting stronger correlations compared to relatively low or no impacts in other tree species. Essentially, not only a tree species’ visibly unique traits (i.e. phenology, morphology), but also its physiological differences compared to other species, appear to drive especially arthropod abundances in higher trophic levels.

With δ13C/12C often used as a general proxy for moisture stress in plants^23^, our results indicate that arthropod diversity will likely respond differentially to future drought episodes depending on respective host tree species. For example, herbivore density and abundance increased with decreased δ13C/12C on one species, but on another species, there was a converse relationship. Under future scenarios of moisture stress, altering foliar δ13C/12C in any one direction might therefore not have predictable herbivore responses, i.e. that stressed trees are more susceptible to herbivore attack, or that stressed trees provide less nutritious leaves and dispel herbivores. Instead, we might expect to find a plethora of idiosyncratic responses to moisture stress, which includes tree intraspecific differences. Conditions in which trees are found may promote these differences, for example when grown closer to perennial streams, or forest edges. Trees in the current study were chosen based on a range of different local conditions (such as distance to streams, rockiness, slope), but these conditions were not considered here. It is well-known that certain tree species prefer wetter microsites within diverse forest communities, and during adverse dry spells, their overall fitness could be more, or less, resilient compared to trees growing in drier microsites. Other species, that grow in a range of microsites, from dry to wet, might reveal intraspecific responses to dry spells, from which differential arthropod responses toward individual trees can be expected. Thus, there will likely be no uniformity in response by canopy arthropod diversity towards increased environmental and climatic changes, as has been suggested for the interactions between herbivorous insects and trees globally^15^.

It is important to be clear on the interaction between carbon-based plant physiology and arthropod diversity in tree canopies, especially under predictions of elevated levels of CO2 globally^41^. Increases in CO2 in the atmosphere will promote plant productivity, likely without simultaneous increases in nutrient uptake^42^. In turn, this might increase the C/N ratio in many plants, often an important indication of both food quality and plant defences^40^. Exposing various *Quercus*-species to elevated levels of CO2 led not only to a rise in foliar C/N ratios, but also to a decrease in associated insect herbivory^43^. Furthermore, insect herbivores will have reduced fitness under elevated CO2 levels, through reduced growth rates and longer development time, as well as reductions in food conversion efficiency^44^. However, increases in C/N may trigger increased herbivore consumption rates, to compensate for diluted nitrogen concentrations in leaves^45^. More compensating strategies by insect herbivores, during increases in C/N, include increased nitrogen utilization efficiency^46^ and stimulation of enzymes detoxifying secondary metabolites in leaves^47^. This means that increased C/N ratios would not necessarily guarantee decreased herbivore performance. Here, C/N ratio correlations with arthropod diversity varied between positive, neutral, and negative, depending on the focal tree species, and showing great interspecific variability in foliar C/N. This suggests that tree species will respond differentially towards elevated CO2 levels, as they do to drought^13^, and lead to many stressors on existing plant–insect interactions.

In conclusion, under future change scenarios, multifaceted responses in canopy arthropod diversity, that are difficult to predict, can be expected. This is especially relevant in small, isolated forest patches, such as the Afro-temperate forests studied here, with limited opportunities for arthropod dispersal between forest patches. Moreover, for many arthropod species, being host-recurrent, shifting from one host species to another during stressful conditions will also not be possible^48^. Conversely, other, generalist arthropods might be able to move between host species, and become more numerous^15^. Ecological impacts of drought, or shifts in climatic regimes, could therefore cause arthropod populations to undergo great changes, threatening ecological integrity. However, host-specific responses of different arthropod guilds towards plant physiology make predictions on how future climates might be shaping canopy communities especially difficult. Our results indicate that these responses encompass factors associated with tree species, plot characteristics, and plant physiology, notwithstanding the direct impacts of environmental change on insect physiology and phenology.

## Methods

### Study area

**Figure 2:**
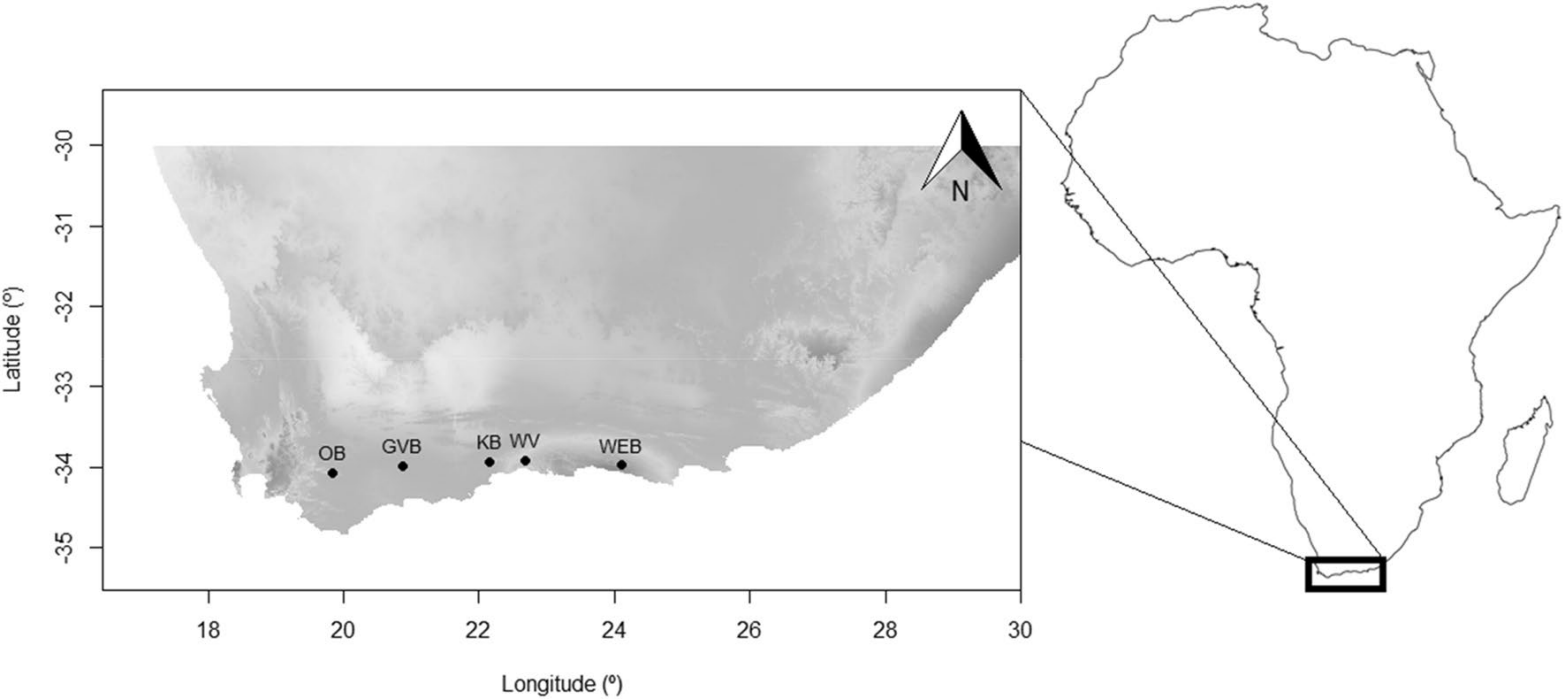
Study region in Africa, showing the five forests from which arthropods were sampled. OB = Oubos, GVB = Grootvadersbosch, KB = Kleinbos, WV = Woodville, WEB = Witelsbos*.* Mean annual temperature for southern Afro-temperate forests is 16.7 °C, characterized by cool winters (8–20 °C) and warm summer (13–25 °C) months. The three western study forests ranged in elevation between 370 – 410 m above sea level, and the two eastern forests, closer to the coast, are located ca. 250 m.a.s.l. Map generated using R statistical software (rstudio.com) version 3.6.2 through *ggplot2*, *raster* and *rgdal* packages with shape file imported from the GADM database (GADM.org).

This study was conducted in five southern Afro-temperate forests from Riviersonderend in the west (Lat -34.04; lon 19.83) to Witelsbos in the east (Lat -33.98; lon 24.11), in the southern Cape of South Africa (Fig. 2). Southern Afro-temperate forests grow on nutrient-poor soils and receive rain all year. Variations in nutrient availability for particular forest trees might arise from differences in leaf-litter nutrient release^49^, competition with other plants^50^, and/or moisture availability^51^, especially in nutrient-poor soils. Average annual rainfall at the five study sites for the five years leading up to sampling (2012–2016) was 1003.64 mm ± 47.10 (s.e.m.), with no significant differences between sites (Supplementary Fig. S14 online).

### Tree selection and arthropod collection

Eight focal tree species were selected, including the three most dominant species in southern Afro-temperate forests (*O. c. macrocarpa*, *R. melanophloeos* and *P. latifolius*), and five species, of various levels of dominance and phylogenetic relatedness (Supplementary table S3 online), commonly encountered throughout the study region^52^. Three individuals of each species were selected per forest, considering general accessibility, tree size (DBH > 50 cm, height > 12 m < 28 m), understorey density (< 20%), and > 15 m apart. These trees also represent a range of different soil depths (according to rockiness of the terrain), distances from surface water (annual streams), and competition from other trees (canopy densities). Accordingly, 24 individual trees were selected per forest, with 15 individual trees per species across the five forests (120 tree individuals in total).

Trees were treated with insecticide fog early morning or late afternoon (05:00–07:00, or after 18:00), under windless conditions, to avoid fog scatter^11^. Trees were treated over two summer months, January and February 2017, corresponding to peak activity for arthropods^53^. We used a Typhoon hand-held fogging machine (45 L/hr solution output) and a pyrethroid insecticide blend (1% deltamethrin, 0.6% permethrin, 6% piperonyl butoxide, 5% aromatic hydrocarbon solvent and 88% diesel), obtained from Dyna-Fog Africa. This machine provides a consistent, warm cloud of fog rising in cool ambient forest conditions. Fog was emitted at ground level for 2 min, to avoid lateral movement into neighbouring trees, while ensuring the entire tree was enveloped in the fog. Ground level fogging has limitations compared to rope-and-pulley systems^54^; fog may not reach all parts of the tree in similar densities. However, it enables quicker implementation and larger mobility of the handler to reach as many trees as possible during limited time of prevailing favourable conditions. Selected trees had no visible epiphytes, flowers, or fruit. Where understorey was present, vegetation was lodged away from the immediate fogging area, or physically removed. Two collecting sheets, each 320 cm × 148 cm in size (= 9.47 m^2^), were placed underneath the crown of each focal tree, while avoiding areas that had overlapping branches of other trees, and suspended ca. 1 m from the forest floor using steel stakes and rope to avoid litter fauna moving onto sheets. A waiting time of 50 min after insecticide application was given to ensure maximal collection time before larger arthropods started to recover. Arthropods on collecting sheets were transferred to collecting jars containing 70% ethanol.

### Arthropods

Collected arthropods were sorted to morphospecies, and assigned to an arthropod order. Where possible, specimens were identified to family level, with spiders and ants identified to genus and/or species level. Additionally, all arthropods were grouped according to functional feeding guild using field guides^55^ and by examining their mouthparts^56^, specific to life stage. Nectarivores, frugivores, granivores, xylophages and phytophages, including combinations of these, were collectively classified under *herbivores*. All predators and parasitoids were classified under *predators*. Fungivores, scavengers, omnivores, scatophages and saprophages, including combinations of these, were classified as *detritivores*. Many fly species have unknown or no feeding strategies as adults. Therefore, considering the life stage collected, species that could not be confidently placed in the categories of herbivore, predator or detritivore, were placed in a separate category termed *tourist*, similar to previous canopy research^57,58^. All ants, having a wide range of diets and unique social structures, were treated as a separate group. Parasites and pollinators comprised relatively few individuals, and were excluded from guild analyses. However, they were included with all other guilds in the *overall* category. A reference collection of all morphospecies is in the Entomology Museum, Stellenbosch University, but spiders were deposited in the South African National Collection of Arachnida, Pretoria, and all hymenopterans, including the ants, at Iziko Museum, Cape Town.

### Plant characteristics

We collected random, mature leaves from the lower branches of each individual focal tree using a pole pruner and a ladder. Leaves were air dried for 4 months in brown paper bags. Then, 0.02 g powdered dry leaf material was sent to the Stable Isotope Laboratory at the Department of Archaeology, University of Cape Town, South Africa to determine total nitrogen content (N), total carbon content (C), carbon: nitrogen ratio (C/N), δ15N/14N ratio (δ15N/14N) and δ13C/12C ratio (δ13C/12C) for each individual tree. Around each focal tree, a circular plot was established with a radius of 8 m (= plot size of ca. 200 m^2^). In each plot, the DBH of the focal tree was measured, its height estimated by a single observer using the mechanical method^59^, and its percentage canopy cover estimated relative to the plot. Where the focal tree covered the whole plot, a cover rating of 100% was given, while a focal tree covering one quarter of the plot was given a cover rating of 25%. For all other trees in the plot with a DBH larger than 15 cm, we determined the species identity and percentage canopy cover. Thus, due to canopy overlap, the total cover per plot could be > 100%.

### Statistical analyses

#### Effect of tree identity and plot characteristics on tree physiological features

Data for N and C were subjected to Yeo-Johnson and inverse hyperbolic sine transformation respectively using the *bestNormalize* package in R version 3.5.1^60^ to normalise distributions. Data for δ15N/14N, C/N and δ13C/12C had normal distributions, determined through Shapiro-Wilks W statistics, and not transformed. Linear mixed-effect modelling (LMMs) was used to evaluate the effect of various plot factors on these data, using the *lme4* package in R^61^. Each model contained the variables host tree identity (species), host tree canopy cover, host same-species canopy cover in plot, total plot tree species richness, and plot canopy cover, with forest site (re: patch) included as random variable in the model^62^. Pairwise differences for the variables for which focal tree species had a significant influence were separated using a Tukey post-hoc test^63^.

#### Effect of tree identity and plot characteristics on canopy arthropod diversity

Species estimates were performed in PRIMER 6, using the Chao2 and Jacknife2 indices^64^. Both abundance and species density data for the respective guilds were tested for overdispersion in R. Overdispersed data were analysed using the negative binomial family, with equidispersed data analysed using the Poisson distribution family. Generalized linear mixed models were constructed for both abundance and species density using the package *glmmTMB* in R^65^. This was done for each of the respective guilds, with each model containing the variables host tree species, host tree canopy cover, host same-species cover in plot, plot tree species richness, and plot canopy cover, with forest site included as random variable. Pairwise differences for abundance and species density for which host tree species revealed significance, were separated using a Tukey post-hoc test using the *emmeans* package in R^63^.

At the small spatial scale, analyses of arthropod assemblage composition can be a more sensitive tool than analyses of species density and abundance data alone^66^. To determine differences in arthropod assemblages between selected tree species, we conducted permutational multivariate analysis of variance (PERMANOVA) in the programme PRIMER 6^64^, on square-root transformed data, using Bray–Curtis similarity matrices. These results were visualised using Canonical Analysis of Principal coordinates (CAP) in the same programme. The respective effects of the different variables mentioned above on arthropod assemblage composition were determined using distance-based linear modelling (DistLM) of Bray–Curtis similarity matrices, using specified selection in PRIMER 6. This method allows for the addition of variables to the analyses based on their total variation explained, until no further variables improved the model based on AICc^67^.

#### Effect of intraspecific physiological variation on arthropod diversity

To test the extent to which a tree’s physiological features explained variation in arthropod species density and abundance for each of the arthropod groups, several candidate models were evaluated for each tree species separately. The full model included the variables N, C, δ15N/14N, δ13C/12C and C/N, with forest site included as random variable, and response variable being either abundance or species density for each arthropod guild. The candidate models for each guild totalled 32, with each model containing a unique combination of variables. Relative support for the models was then determined using the AICc criterion with best model selection using the package *AICcmodavg* in R, after which Generalised Linear Mixed Models (GLMMs) with poisson distributions were constructed using the candidate model with lowest AICc. To explain variation in arthropod assemblage composition as described by the five plant physiological characteristics, distance-based linear modelling (DistLM) was performed for each arthropod guild, on each tree species, based on Bray–Curtis similarity matrices, using specified selection in PRIMER 6^64^.

## Supplementary information

Supplementary Information.
