# Supplementary material for: Tree canopy arthropods have idiosyncratic responses to plant ecophysiological traits in a warm temperate forest complex
Source: Sci Rep. 2020 Nov 16;10:19905. doi: 10.1038/s41598-020-76868-8 (PMC7670454; doi:10.1038/s41598-020-76868-8)
Supplement: Supplementary file 1 — Supplementary Information. [file 41598_2020_76868_MOESM1_ESM.docx]

**Supplementary material to:**

**Tree canopy arthropods have idiosyncratic responses to plant ecophysiological traits in a warm temperate forest complex**

Rudi C. Swart, Michael J. Samways, Francois Roets

Department of Conservation Ecology and Entomology, Stellenbosch University, Private Bag X1, Matieland 7602, South Africa

Corresponding author: swartrudolph90@gmail.com

**Table S1**: Reference list of arthropod morphospecies sampled from 120 individual tree canopies in southern Afrotemperate forests.

| Class | Subclass | Order | Suborder | Family / Subfamily | Species | Code | Guild | Total |
| --- | --- | --- | --- | --- | --- | --- | --- | --- |
| Arachnida | Acari | Sarcoptiformes | Oribatida |  |  | C19 | Detritivore | 1 |
| Arachnida | Acari | Parasitiformes |  |  |  | T12 | Parasite | 15 |
| Arachnida | Acari | Parasitiformes |  |  |  | T14 | Parasite | 1 |
| Arachnida | Acari | Parasitiformes |  |  |  | T15 | Parasite | 4 |
| Arachnida | Acari | Parasitiformes |  |  |  | T16 | Parasite | 2 |
| Arachnida | Acari | Parasitiformes |  |  |  | T19 | Parasite | 1 |
| Arachnida | Acari | Parasitiformes |  |  |  | T2 | Parasite | 137 |
| Arachnida | Acari | Parasitiformes |  |  |  | T21 | Parasite | 2 |
| Arachnida | Acari | Parasitiformes |  |  |  | T3 | Parasite | 1 |
| Arachnida | Acari | Parasitiformes |  |  |  | T5 | Parasite | 4 |
| Arachnida | Acari | Parasitiformes |  |  |  | T20 | Parasite | 1 |
| Arachnida | Acari | Parasitiformes |  |  |  | T4 | Parasite | 574 |
| Arachnida |  | Araneae |  | Amaurobiidae | *Chresiona convexa* | A19 | Predator | 15 |
| Arachnida |  | Araneae |  | Amaurobiidae | *Chresiona* sp. | A60 | Predator | 12 |
| Arachnida |  | Araneae |  | Amaurobiidae | *Obatala armata* | A64 | Predator | 12 |
| Arachnida |  | Araneae |  | Anapidae | *Crozetulus rhodesiensis* | A168 | Predator | 19 |
| Arachnida |  | Araneae |  | Anapidae | Crozetulus sp. | A210 | Predator | 5 |
| Arachnida |  | Araneae |  | Anapidae | sp. *2* | A160 | Predator | 45 |
| Arachnida |  | Araneae |  | Anapidae | sp. *3* | A26 | Predator | 1 |
| Arachnida |  | Araneae |  | Anapidae | sp. *4* | A101 | Predator | 4 |
| Arachnida |  | Araneae |  | Anapidae | sp. *5* | A107 | Predator | 1 |
| Arachnida |  | Araneae |  | Anapidae | sp. *6* | A118 | Predator | 2 |
| Arachnida |  | Araneae |  | Anapidae | sp. *7* | A112 | Predator | 5 |
| Arachnida |  | Araneae |  | Araneidae | *Araneus holzapfelae* | A248 | Predator | 1 |
| Arachnida |  | Araneae |  | Araneidae | *Araneus* sp. *1* | A172 | Predator | 1 |
| Arachnida |  | Araneae |  | Araneidae | *Araneus* sp. *2* | A163 | Predator | 10 |
| Arachnida |  | Araneae |  | Araneidae | *Caerostris sexcuspidata* | A171 | Predator | 16 |
| Arachnida |  | Araneae |  | Araneidae | *Cyclosa insulana* | A68 | Predator | 6 |
| Arachnida |  | Araneae |  | Araneidae | *Cyphalonotus larvatus* | A173 | Predator | 2 |
| Arachnida |  | Araneae |  | Araneidae | *Eriovixia excelsa* | A1 | Predator | 16 |
| Arachnida |  | Araneae |  | Araneidae | *Eriovixia* sp. | A39 | Predator | 1 |
| Arachnida |  | Araneae |  | Araneidae | *Gasteracantha sanguinolenta* | A6 | Predator | 8 |
| Arachnida |  | Araneae |  | Araneidae | *Ideocaira triquetra* | A91 | Predator | 15 |
| Arachnida |  | Araneae |  | Araneidae | *Larinioides* sp. | A96 | Predator | 5 |
| Arachnida |  | Araneae |  | Araneidae | *Neoscona* sp. | A32 | Predator | 39 |
| Arachnida |  | Araneae |  | Araneidae | *Neoscona subfusca* | A116 | Predator | 24 |
| Arachnida |  | Araneae |  | Araneidae | *Prasonica* sp. | A51 | Predator | 30 |
| Arachnida |  | Araneae |  | Cheircanthiidae | *Cheiracanthium* sp. | A137 | Predator | 10 |
| Arachnida |  | Araneae |  | Clubionidae | *Clubiona* sp. | A132 | Predator | 430 |
| Arachnida |  | Araneae |  | Clubionidae | sp. *1* | A2 | Predator | 428 |
| Arachnida |  | Araneae |  | Clubionidae | sp. *2* | A111 | Predator | 11 |
| Arachnida |  | Araneae |  | Clubionidae | sp. *3* | A4 | Predator | 2 |
| Arachnida |  | Araneae |  | Clubionidae | sp. *4* | A147 | Predator | 6 |
| Arachnida |  | Araneae |  | Deinopidae | *Menneus* sp. | A252 | Predator | 8 |
| Arachnida |  | Araneae |  | Dictynidae | *Dictyna* sp. | A143 | Predator | 2 |
| Arachnida |  | Araneae |  | Dictynidae | *Mashimo leleupi* | A63 | Predator | 11 |
| Arachnida |  | Araneae |  | Eresidae | *Gandanameno fumosa* | A3 | Predator | 1 |
| Arachnida |  | Araneae |  | Eutichuridae | *Cheiramiona* sp. | A33 | Predator | 2 |
| Arachnida |  | Araneae |  | Gnaphosidae | *Aphantaulax signicollis* | A189 | Predator | 8 |
| Arachnida |  | Araneae |  | Gnaphosiidae | sp. *1* | A156 | Predator | 3 |
| Arachnida |  | Araneae |  | Hahniidae | *Hahnia* sp. | A201 | Predator | 1 |
| Arachnida |  | Araneae |  | Hersiliidae | *Hersilia setifrons* | A290 | Predator | 3 |
| Arachnida |  | Araneae |  | Linyphiidae | *Afribactrus stylifrons* | A212 | Predator | 8 |
| Arachnida |  | Araneae |  | Linyphiidae | *Mecynidis dentipalpis* | A93 | Predator | 11 |
| Arachnida |  | Araneae |  | Linyphiidae | *Pelecopsis* sp. | A142 | Predator | 33 |
| Arachnida |  | Araneae |  | Linyphiidae | sp. *1* | A100 | Predator | 8 |
| Arachnida |  | Araneae |  | Mimetidae | *Ero capensis* | A49 | Predator | 9 |
| Arachnida |  | Araneae |  | Mimetidae | *Mimetus* sp. | A167 | Predator | 10 |
| Arachnida |  | Araneae |  | Oonopidae | *Australoonops granulatus* | A81 | Predator | 8 |
| Arachnida |  | Araneae |  | Oxyopidae | *Hamataliwa strandi* | A73 | Predator | 3 |
| Arachnida |  | Araneae |  | Philodromidae | *Philodomus* sp. *1* | A282 | Predator | 4 |
| Arachnida |  | Araneae |  | Pholcidae | *Quamtana knysna* | A82 | Predator | 17 |
| Arachnida |  | Araneae |  | Salticidae | *Myrmarachne* sp. | A97 | Predator | 41 |
| Arachnida |  | Araneae |  | Salticidae | *Thyene coccineovittata* | A13 | Predator | 90 |
| Arachnida |  | Araneae |  | Scytodidae | *Scytodes cedri* | A152 | Predator | 65 |
| Arachnida |  | Araneae |  | Scytodidae | *Scytodes* sp. | A44 | Predator | 2 |
| Arachnida |  | Araneae |  | Tetragnathidae | *Leucauge argyrescens* | A35 | Predator | 15 |
| Arachnida |  | Araneae |  | Tetragnathidae | *Leucauge decorata* | A36 | Predator | 42 |
| Arachnida |  | Araneae |  | Tetragnathidae | *Leucauge* sp. | A276 | Predator | 2 |
| Arachnida |  | Araneae |  | Tetragnathidae | *Tetragnatha ceylonica* | A202 | Predator | 1 |
| Arachnida |  | Araneae |  | Theridiidae | *Argyrodes convivans* | A103 | Predator | 19 |
| Arachnida |  | Araneae |  | Theridiidae | *Argyrodes* sp. | A15 | Predator | 71 |
| Arachnida |  | Araneae |  | Theridiidae | *Episinus* sp. | A121 | Predator | 18 |
| Arachnida |  | Araneae |  | Theridiidae | *Latrodectus geometricus* | A207 | Predator | 4 |
| Arachnida |  | Araneae |  | Theridiidae | *Phoroncidia* sp. | A25 | Predator | 41 |
| Arachnida |  | Araneae |  | Theridiidae | *Phycosoma* sp. | A74 | Predator | 19 |
| Arachnida |  | Araneae |  | Theridiidae | *Platnickina mneon* | A105 | Predator | 1 |
| Arachnida |  | Araneae |  | Theridiidae | sp. *1* | A285 | Predator | 1 |
| Arachnida |  | Araneae |  | Theridiidae | *Theridion purcelli* | A5 | Predator | 9 |
| Arachnida |  | Araneae |  | Theridiidae | *Theridion* sp. *1* | A88 | Predator | 2 |
| Arachnida |  | Araneae |  | Theridiidae | *Theridion* sp. *2* | A154 | Predator | 195 |
| Arachnida |  | Araneae |  | Theridiidae | *Theridion* sp. *3* | A90 | Predator | 7 |
| Arachnida |  | Araneae |  | Theridiidae | *Theridula* sp. *2* | A104 | Predator | 4 |
| Arachnida |  | Araneae |  | Theridiosomatidae | *Baalzebub* sp. | A129 | Predator | 17 |
| Arachnida |  | Araneae |  | Theridiosomatidae | sp. *1* | A146 | Predator | 3 |
| Arachnida |  | Araneae |  | Theridiosomatidae | sp. *2* | A29 | Predator | 38 |
| Arachnida |  | Araneae |  | Theridiosomatidae | sp. *3* | A99 | Predator | 1 |
| Arachnida |  | Araneae |  | Thomisidae | *Diaea dorsata* | A24 | Predator | 2 |
| Arachnida |  | Araneae |  | Thomisidae | *Diaea puncta* | A50 | Predator | 38 |
| Arachnida |  | Araneae |  | Thomisidae | *Oxytate leruthi* | A66 | Predator | 40 |
| Arachnida |  | Araneae |  | Thomisidae | *Phaenopoma nigropunctatum* | A12 | Predator | 9 |
| Arachnida |  | Araneae |  | Thomisidae | *Pherecydes* sp. *1* | A69 | Predator | 16 |
| Arachnida |  | Araneae |  | Thomisidae | *Pherecydes* sp. *2* | A325 | Predator | 1 |
| Arachnida |  | Araneae |  | Thomisidae | *Phrynarachne melloleitaoi* | A123 | Predator | 5 |
| Arachnida |  | Araneae |  | Thomisidae | *Simorcus capensis* | A174 | Predator | 1 |
| Arachnida |  | Araneae |  | Thomisidae | *Synema vallotoni* | A272 | Predator | 3 |
| Arachnida |  | Araneae |  | Thomisidae | *Thomisus scrupeus* | A287 | Predator | 1 |
| Arachnida |  | Araneae |  | Thomisidae | *Tmarus cameliformis* | A175 | Predator | 19 |
| Arachnida |  | Araneae |  | Thomisidae | *Tmarus cancellatus* | A14 | Predator | 92 |
| Arachnida |  | Araneae |  | Thomisidae | *Tmarus* sp. | A145 | Predator | 7 |
| Arachnida |  | Araneae |  | Trachelidae | *Afroceto martini* | A85 | Predator | 3 |
| Arachnida |  | Araneae |  | Trachelidae | *Jocquestus capensis* | A108 | Predator | 7 |
| Arachnida |  | Araneae |  | Trachelidae | sp. *1* | A109 | Predator | 6 |
| Arachnida |  | Araneae |  | Trachelidae | sp. *2* | A249 | Predator | 1 |
| Arachnida |  | Araneae |  | Trachelidae | sp. *3* | A257 | Predator | 1 |
| Arachnida |  | Araneae |  | Trachelidae | sp. *4* | A284 | Predator | 1 |
| Arachnida |  | Araneae |  | Trachelidae | sp. *5* | A317 | Predator | 1 |
| Arachnida |  | Araneae |  | Trachelidae | *Trachelas penicillus* | A28 | Predator | 48 |
| Arachnida |  | Araneae |  | Trachelidae | *Trachelas setosus* | A130 | Predator | 9 |
| Arachnida |  | Araneae |  | Trochanteriidae | *Platyoides* sp. | A305 | Predator | 4 |
| Arachnida |  | Araneae |  | Uloboridae | *Miagrammopes* sp. | A298 | Predator | 1 |
| Arachnida |  | Araneae |  | Uloboridae | *Uloborus* sp. | A127 | Predator | 7 |
| Arachnida |  | Araneae |  | Uloboridae | *Zosis geniculata* | A155 | Predator | 6 |
| Arachnida |  | Araneae |  | Zodariidae | *Chariobas cylindraceus* | A193 | Predator | 6 |
| Arachnida |  | Opiliones |  |  | sp. *1* | A122 | Predator | 2 |
| Arachnida |  | Opiliones |  |  | sp. *2* | A43 | Predator | 7 |
| Arachnida |  | Pseudoscorpiones |  |  |  | P1 | Predator | 303 |
| Arachnida |  | Pseudoscorpiones |  |  |  | P2 | Predator | 9 |
| Arachnida |  | Pseudoscorpiones |  |  |  | P3 | Predator | 74 |
| Arachnida |  | Pseudoscorpiones |  |  |  | P4 | Predator | 269 |
| Arachnida |  | Pseudoscorpiones |  |  |  | P5 | Predator | 12 |
| Arachnida |  | Pseudoscorpiones |  |  |  | P6 | Predator | 2 |
| Arachnida |  | Pseudoscorpiones |  |  |  | P7 | Predator | 5 |
| Arachnida |  | Pseudoscorpiones |  |  |  | P8 | Predator | 2 |
| Arachnida | Acari | Sarcoptiformes |  | Phthiracaridae |  | T10 | Detritivore | 2 |
| Arachnida | Acari | Sarcoptiformes |  | Phthiracaridae |  | T6 | Detritivore | 31 |
| Arachnida | Acari | Sarcoptiformes |  |  |  | T11 | Detritivore | 63 |
| Arachnida | Acari | Sarcoptiformes |  |  |  | T17 | Detritivore | 1 |
| Arachnida | Acari | Sarcoptiformes |  |  |  | T18 | Detritivore | 2 |
| Arachnida | Acari | Sarcoptiformes |  |  |  | T8 | Detritivore | 5 |
| Arachnida | Acari | Trombidiformes |  | Caeculidae |  | T1 | Predator | 274 |
| Arachnida | Acari | Trombidiformes |  | Caeculidae |  | T13 | Predator | 1 |
| Arachnida | Acari | Trombidiformes |  | Caeculidae |  | T9 | Predator | 21 |
| Arachnida | Acari | Trombidiformes |  |  |  | T7 | Detritivore | 52 |
| Chilopoda |  | Scolopendromorpha |  |  |  | CE1 | Predator | 5 |
| Chilopoda |  | Scolopendromorpha |  |  |  | CE2 | Predator | 3 |
| Chilopoda |  | Scolopendromorpha |  |  |  | CE3 | Predator | 8 |
| Chilopoda |  | Scolopendromorpha |  |  |  | CE4 | Predator | 2 |
| Chilopoda |  | Scutigeromorpha |  |  |  | CE5 | Predator | 1 |
| Collembola |  |  |  | Poduridae |  | I10 | Detritivore | 2 |
| Collembola |  |  |  | Poduridae |  | I13 | Detritivore | 27 |
| Collembola |  |  |  | Poduridae |  | I9 | Detritivore | 4 |
| Collembola |  |  |  | Sminthuridae |  | U2 | Detritivore | 7 |
| Collembola |  |  |  |  |  | I12 | Detritivore | 2 |
| Collembola |  |  |  |  |  | I14 | Detritivore | 2 |
| Collembola |  |  |  |  |  | I15 | Detritivore | 1 |
| Collembola |  |  |  |  |  | U4 | Detritivore | 1 |
| Insecta |  | Archaeognatha |  | Meinertellidae |  | J1 | Detritivore | 118 |
| Insecta |  | Archaeognatha |  | Meinertellidae |  | J3 | Detritivore | 43 |
| Insecta |  | Blattodea |  | Blaberidae |  | B13 | Detritivore | 2 |
| Insecta |  | Blattodea |  | Blaberidae |  | B15 | Detritivore | 2 |
| Insecta |  | Blattodea |  | Blaberidae |  | B17 | Detritivore | 1 |
| Insecta |  | Blattodea |  | Blaberidae |  | B18 | Detritivore | 1 |
| Insecta |  | Blattodea |  | Blaberidae |  | B3 | Detritivore | 1 |
| Insecta |  | Blattodea |  | Blaberidae |  | B6 | Detritivore | 7 |
| Insecta |  | Blattodea |  | Blaberidae |  | B8 | Detritivore | 2 |
| Insecta |  | Blattodea |  | Blatellidae |  | B10 | Detritivore | 1 |
| Insecta |  | Blattodea |  | Blatellidae |  | B11 | Detritivore | 1 |
| Insecta |  | Blattodea |  | Blatellidae |  | B14 | Detritivore | 13 |
| Insecta |  | Blattodea |  | Blatellidae |  | B4 | Detritivore | 174 |
| Insecta |  | Blattodea |  | Blatellidae |  | B5 | Detritivore | 415 |
| Insecta |  | Blattodea |  | Blatellidae |  | C325 | Detritivore | 5 |
| Insecta |  | Blattodea |  | Blattidae |  | B1 | Detritivore | 129 |
| Insecta |  | Blattodea |  | Blattidae |  | B16 | Detritivore | 6 |
| Insecta |  | Blattodea |  | Blattidae |  | B2 | Detritivore | 36 |
| Insecta |  | Blattodea |  | Blattidae |  | B7 | Detritivore | 4 |
| Insecta |  | Blattodea |  | Termitidae |  | E3 | Detritivore | 1 |
| Insecta |  | Blattodea |  | Termitidae |  | V3 | Detritivore | 1 |
| Insecta |  | Coleoptera |  | Aderidae |  | C198 | Detritivore | 1 |
| Insecta |  | Coleoptera |  | Aderidae |  | C202 | Detritivore | 4 |
| Insecta |  | Coleoptera |  | Aderidae |  | C230 | Detritivore | 2 |
| Insecta |  | Coleoptera |  | Anobiidae |  | C128 | Herbivore | 4 |
| Insecta |  | Coleoptera |  | Anobiidae |  | C143 | Herbivore | 5 |
| Insecta |  | Coleoptera |  | Anobiidae |  | C147 | Herbivore | 1 |
| Insecta |  | Coleoptera |  | Anobiidae |  | C172 | Herbivore | 1 |
| Insecta |  | Coleoptera |  | Anobiidae |  | C174 | Herbivore | 1 |
| Insecta |  | Coleoptera |  | Anobiidae |  | C185 | Herbivore | 4 |
| Insecta |  | Coleoptera |  | Anobiidae |  | C192 | Herbivore | 7 |
| Insecta |  | Coleoptera |  | Anobiidae |  | C208 | Herbivore | 1 |
| Insecta |  | Coleoptera |  | Anobiidae |  | C241 | Herbivore | 1 |
| Insecta |  | Coleoptera |  | Anobiidae |  | C257 | Herbivore | 1 |
| Insecta |  | Coleoptera |  | Anobiidae |  | C287 | Herbivore | 1 |
| Insecta |  | Coleoptera |  | Anobiidae |  | C298 | Herbivore | 2 |
| Insecta |  | Coleoptera |  | Anobiidae |  | C323 | Herbivore | 1 |
| Insecta |  | Coleoptera |  | Anobiidae |  | C326 | Herbivore | 1 |
| Insecta |  | Coleoptera |  | Anobiidae |  | C334 | Herbivore | 2 |
| Insecta |  | Coleoptera |  | Anobiidae |  | C336 | Herbivore | 2 |
| Insecta |  | Coleoptera |  | Anobiidae |  | C346 | Herbivore | 1 |
| Insecta |  | Coleoptera |  | Anobiidae |  | C93 | Herbivore | 1 |
| Insecta |  | Coleoptera |  | Anthicidae |  | C1 | Detritivore | 29 |
| Insecta |  | Coleoptera |  | Anthicidae |  | C196 | Detritivore | 10 |
| Insecta |  | Coleoptera |  | Anthicidae |  | C214 | Detritivore | 1 |
| Insecta |  | Coleoptera |  | Anthicidae |  | C220 | Detritivore | 2 |
| Insecta |  | Coleoptera |  | Anthicidae |  | C229 | Detritivore | 1 |
| Insecta |  | Coleoptera |  | Anthicidae |  | C235 | Detritivore | 10 |
| Insecta |  | Coleoptera |  | Anthicidae |  | C242 | Detritivore | 3 |
| Insecta |  | Coleoptera |  | Anthicidae |  | C37 | Detritivore | 5 |
| Insecta |  | Coleoptera |  | Anthicidae |  | C47 | Detritivore | 212 |
| Insecta |  | Coleoptera |  | Anthicidae |  | C63 | Detritivore | 5 |
| Insecta |  | Coleoptera |  | Apionidae |  | C348 | Herbivore | 1 |
| Insecta |  | Coleoptera |  | Bostrichidae |  | C110 | Herbivore | 3 |
| Insecta |  | Coleoptera |  | Bostrichidae |  | C111 | Herbivore | 20 |
| Insecta |  | Coleoptera |  | Bostrichidae |  | C130 | Herbivore | 4 |
| Insecta |  | Coleoptera |  | Bostrichidae |  | C15 | Herbivore | 1 |
| Insecta |  | Coleoptera |  | Bostrichidae |  | C254 | Herbivore | 4 |
| Insecta |  | Coleoptera |  | Bostrichidae |  | C328 | Herbivore | 1 |
| Insecta |  | Coleoptera |  | Bostrichidae |  | C344 | Herbivore | 1 |
| Insecta |  | Coleoptera |  | Bostrichidae |  | LA15 | Herbivore | 1 |
| Insecta |  | Coleoptera |  | Bostrichidae |  | LA29 | Herbivore | 1 |
| Insecta |  | Coleoptera |  | Bostrichidae |  | LA36 | Herbivore | 1 |
| Insecta |  | Coleoptera |  | Bostrichidae |  | LA58 | Herbivore | 1 |
| Insecta |  | Coleoptera |  | Brentidae |  | C207 | Detritivore | 2 |
| Insecta |  | Coleoptera |  | Bruchidae |  | C125 | Herbivore | 1 |
| Insecta |  | Coleoptera |  | Bruchidae |  | C203 | Herbivore | 1 |
| Insecta |  | Coleoptera |  | Bruchidae |  | C30 | Herbivore | 3 |
| Insecta |  | Coleoptera |  | Buprestidae |  | C281 | Herbivore | 2 |
| Insecta |  | Coleoptera |  | Byrrhidae |  | C139 | Herbivore | 1 |
| Insecta |  | Coleoptera |  | Byrrhidae |  | C181 | Herbivore | 5 |
| Insecta |  | Coleoptera |  | Byrrhidae |  | C217 | Herbivore | 12 |
| Insecta |  | Coleoptera |  | Byrrhidae |  | C277 | Herbivore | 1 |
| Insecta |  | Coleoptera |  | Byrrhidae |  | C302 | Herbivore | 1 |
| Insecta |  | Coleoptera |  | Cantharidae |  | C24 | Predator | 1 |
| Insecta |  | Coleoptera |  | Carabidae |  | C134 | Predator | 13 |
| Insecta |  | Coleoptera |  | Carabidae |  | C164 | Predator | 5 |
| Insecta |  | Coleoptera |  | Carabidae |  | C17 | Predator | 2 |
| Insecta |  | Coleoptera |  | Carabidae |  | C195 | Predator | 3 |
| Insecta |  | Coleoptera |  | Carabidae |  | C204 | Predator | 1 |
| Insecta |  | Coleoptera |  | Carabidae |  | C222 | Predator | 1 |
| Insecta |  | Coleoptera |  | Carabidae |  | C25 | Predator | 29 |
| Insecta |  | Coleoptera |  | Carabidae |  | C258 | Predator | 1 |
| Insecta |  | Coleoptera |  | Carabidae |  | C293 | Predator | 3 |
| Insecta |  | Coleoptera |  | Carabidae |  | C327 | Predator | 1 |
| Insecta |  | Coleoptera |  | Carabidae |  | C54 | Predator | 1 |
| Insecta |  | Coleoptera |  | Carabidae |  | C55 | Predator | 1 |
| Insecta |  | Coleoptera |  | Carabidae |  | C7 | Predator | 1 |
| Insecta |  | Coleoptera |  | Carabidae |  | C8 | Predator | 175 |
| Insecta |  | Coleoptera |  | Carabidae |  | C85 | Predator | 20 |
| Insecta |  | Coleoptera |  | Carabidae |  | C9 | Predator | 15 |
| Insecta |  | Coleoptera |  | Carabidae |  | C90 | Predator | 2 |
| Insecta |  | Coleoptera |  | Carabidae |  | LA20 | Predator | 1 |
| Insecta |  | Coleoptera |  | Carabidae |  | LA22 | Predator | 1 |
| Insecta |  | Coleoptera |  | Carabidae |  | LA48 | Predator | 1 |
| Insecta |  | Coleoptera |  | Carabidae |  | LA53 | Predator | 2 |
| Insecta |  | Coleoptera |  | Carabidae |  | LA59 | Predator | 1 |
| Insecta |  | Coleoptera |  | Carabidae |  | LA67 | Predator | 1 |
| Insecta |  | Coleoptera |  | Carabidae |  | LA68 | Predator | 1 |
| Insecta |  | Coleoptera |  | Carabidae |  | LA71 | Predator | 1 |
| Insecta |  | Coleoptera |  | Carabidae |  | LA75 | Predator | 1 |
| Insecta |  | Coleoptera |  | Cerambycidae |  | C136 | Herbivore | 3 |
| Insecta |  | Coleoptera |  | Cerambycidae |  | C178 | Herbivore | 5 |
| Insecta |  | Coleoptera |  | Cerambycidae |  | C232 | Herbivore | 1 |
| Insecta |  | Coleoptera |  | Cerambycidae |  | C312 | Herbivore | 3 |
| Insecta |  | Coleoptera |  | Cerambycidae |  | C44 | Herbivore | 7 |
| Insecta |  | Coleoptera |  | Chrysomelidae |  | C103 | Herbivore | 1 |
| Insecta |  | Coleoptera |  | Chrysomelidae |  | C104 | Herbivore | 1 |
| Insecta |  | Coleoptera |  | Chrysomelidae |  | C105 | Herbivore | 4 |
| Insecta |  | Coleoptera |  | Chrysomelidae |  | C127 | Herbivore | 2 |
| Insecta |  | Coleoptera |  | Chrysomelidae |  | C148 | Herbivore | 17 |
| Insecta |  | Coleoptera |  | Chrysomelidae |  | C157 | Herbivore | 6 |
| Insecta |  | Coleoptera |  | Chrysomelidae |  | C179 | Herbivore | 6 |
| Insecta |  | Coleoptera |  | Chrysomelidae |  | C223 | Herbivore | 1 |
| Insecta |  | Coleoptera |  | Chrysomelidae |  | C23 | Herbivore | 2 |
| Insecta |  | Coleoptera |  | Chrysomelidae |  | C251 | Herbivore | 4 |
| Insecta |  | Coleoptera |  | Chrysomelidae |  | C261 | Herbivore | 1 |
| Insecta |  | Coleoptera |  | Chrysomelidae |  | C263 | Herbivore | 2 |
| Insecta |  | Coleoptera |  | Chrysomelidae |  | C282 | Herbivore | 1 |
| Insecta |  | Coleoptera |  | Chrysomelidae |  | C315 | Herbivore | 2 |
| Insecta |  | Coleoptera |  | Chrysomelidae |  | C316 | Herbivore | 1 |
| Insecta |  | Coleoptera |  | Chrysomelidae |  | C322 | Herbivore | 10 |
| Insecta |  | Coleoptera |  | Chrysomelidae |  | C343 | Herbivore | 1 |
| Insecta |  | Coleoptera |  | Chrysomelidae |  | C350 | Herbivore | 2 |
| Insecta |  | Coleoptera |  | Chrysomelidae |  | C352 | Herbivore | 1 |
| Insecta |  | Coleoptera |  | Chrysomelidae |  | C354 | Herbivore | 1 |
| Insecta |  | Coleoptera |  | Chrysomelidae |  | C38 | Herbivore | 256 |
| Insecta |  | Coleoptera |  | Chrysomelidae |  | C4 | Herbivore | 5 |
| Insecta |  | Coleoptera |  | Chrysomelidae |  | C40 | Herbivore | 163 |
| Insecta |  | Coleoptera |  | Chrysomelidae |  | C45 | Herbivore | 124 |
| Insecta |  | Coleoptera |  | Chrysomelidae |  | C46 | Herbivore | 5 |
| Insecta |  | Coleoptera |  | Chrysomelidae |  | C58 | Herbivore | 11 |
| Insecta |  | Coleoptera |  | Chrysomelidae |  | C65 | Herbivore | 17 |
| Insecta |  | Coleoptera |  | Chrysomelidae |  | C66 | Herbivore | 4 |
| Insecta |  | Coleoptera |  | Chrysomelidae |  | C72 | Herbivore | 5 |
| Insecta |  | Coleoptera |  | Chrysomelidae |  | C88 | Herbivore | 1 |
| Insecta |  | Coleoptera |  | Ciidae |  | C197 | Detritivore | 1 |
| Insecta |  | Coleoptera |  | Ciidae |  | C224 | Detritivore | 1 |
| Insecta |  | Coleoptera |  | Ciidae |  | C250 | Detritivore | 1 |
| Insecta |  | Coleoptera |  | Clambidae |  | C123 | Detritivore | 8 |
| Insecta |  | Coleoptera |  | Clambidae |  | C142 | Detritivore | 10 |
| Insecta |  | Coleoptera |  | Clambidae |  | C253 | Detritivore | 2 |
| Insecta |  | Coleoptera |  | Clambidae |  | C267 | Detritivore | 4 |
| Insecta |  | Coleoptera |  | Clambidae |  | C283 | Detritivore | 1 |
| Insecta |  | Coleoptera |  | Clambidae |  | C329 | Detritivore | 1 |
| Insecta |  | Coleoptera |  | Clambidae |  | C35 | Detritivore | 122 |
| Insecta |  | Coleoptera |  | Cleridae |  | C118 | Predator | 1 |
| Insecta |  | Coleoptera |  | Cleridae |  | C119 | Predator | 4 |
| Insecta |  | Coleoptera |  | Cleridae |  | C122 | Predator | 20 |
| Insecta |  | Coleoptera |  | Cleridae |  | C165 | Predator | 1 |
| Insecta |  | Coleoptera |  | Cleridae |  | C176 | Predator | 3 |
| Insecta |  | Coleoptera |  | Cleridae |  | C215 | Predator | 1 |
| Insecta |  | Coleoptera |  | Cleridae |  | C26 | Predator | 1 |
| Insecta |  | Coleoptera |  | Cleridae |  | C273 | Predator | 2 |
| Insecta |  | Coleoptera |  | Cleridae |  | C294 | Predator | 1 |
| Insecta |  | Coleoptera |  | Cleridae |  | C31 | Predator | 1 |
| Insecta |  | Coleoptera |  | Cleridae |  | C331 | Predator | 1 |
| Insecta |  | Coleoptera |  | Cleridae |  | C68 | Predator | 8 |
| Insecta |  | Coleoptera |  | Cleridae |  | C87 | Predator | 2 |
| Insecta |  | Coleoptera |  | Cleridae |  | C97 | Predator | 2 |
| Insecta |  | Coleoptera |  | Cleridae |  | LA23 | Predator | 8 |
| Insecta |  | Coleoptera |  | Coccinellidae |  | C115 | Predator | 18 |
| Insecta |  | Coleoptera |  | Coccinellidae |  | C117 | Predator | 6 |
| Insecta |  | Coleoptera |  | Coccinellidae |  | C12 | Predator | 48 |
| Insecta |  | Coleoptera |  | Coccinellidae |  | C129 | Predator | 28 |
| Insecta |  | Coleoptera |  | Coccinellidae |  | C16 | Predator | 114 |
| Insecta |  | Coleoptera |  | Coccinellidae |  | C175 | Predator | 19 |
| Insecta |  | Coleoptera |  | Coccinellidae |  | C200 | Predator | 7 |
| Insecta |  | Coleoptera |  | Coccinellidae |  | C201 | Predator | 1 |
| Insecta |  | Coleoptera |  | Coccinellidae |  | C21 | Predator | 11 |
| Insecta |  | Coleoptera |  | Coccinellidae |  | C22 | Predator | 3 |
| Insecta |  | Coleoptera |  | Coccinellidae |  | C238 | Predator | 1 |
| Insecta |  | Coleoptera |  | Coccinellidae |  | C244 | Predator | 12 |
| Insecta |  | Coleoptera |  | Coccinellidae |  | C274 | Predator | 1 |
| Insecta |  | Coleoptera |  | Coccinellidae |  | C292 | Predator | 1 |
| Insecta |  | Coleoptera |  | Coccinellidae |  | C303 | Predator | 2 |
| Insecta |  | Coleoptera |  | Coccinellidae |  | C33 | Predator | 1 |
| Insecta |  | Coleoptera |  | Coccinellidae |  | C353 | Predator | 1 |
| Insecta |  | Coleoptera |  | Coccinellidae |  | C64 | Predator | 2 |
| Insecta |  | Coleoptera |  | Coccinellidae |  | C89 | Predator | 2 |
| Insecta |  | Coleoptera |  | Coccinellidae |  | LA17 | Predator | 4 |
| Insecta |  | Coleoptera |  | Coccinellidae |  | LA31 | Predator | 10 |
| Insecta |  | Coleoptera |  | Colydiidae |  | C338 | Predator | 2 |
| Insecta |  | Coleoptera |  | Corylophidae |  | C140 | Detritivore | 56 |
| Insecta |  | Coleoptera |  | Corylophidae |  | C180 | Detritivore | 2 |
| Insecta |  | Coleoptera |  | Corylophidae |  | C183 | Detritivore | 1 |
| Insecta |  | Coleoptera |  | Corylophidae |  | C191 | Detritivore | 3 |
| Insecta |  | Coleoptera |  | Corylophidae |  | C231 | Detritivore | 1 |
| Insecta |  | Coleoptera |  | Corylophidae |  | C307 | Detritivore | 1 |
| Insecta |  | Coleoptera |  | Cryptophagidae |  | C189 | Detritivore | 4 |
| Insecta |  | Coleoptera |  | Cryptophagidae |  | C213 | Detritivore | 1 |
| Insecta |  | Coleoptera |  | Cryptophagidae |  | C249 | Detritivore | 1 |
| Insecta |  | Coleoptera |  | Cryptophagidae |  | C299 | Detritivore | 3 |
| Insecta |  | Coleoptera |  | Cryptophagidae |  | C306 | Detritivore | 1 |
| Insecta |  | Coleoptera |  | Cryptophagidae |  | C332 | Detritivore | 1 |
| Insecta |  | Coleoptera |  | Cryptophagidae |  | C83 | Detritivore | 65 |
| Insecta |  | Coleoptera |  | Cucujidae |  | C159 | Predator | 13 |
| Insecta |  | Coleoptera |  | Cucujidae |  | C182 | Predator | 2 |
| Insecta |  | Coleoptera |  | Cucujidae |  | C304 | Predator | 1 |
| Insecta |  | Coleoptera |  | Cucujidae |  | C310 | Predator | 1 |
| Insecta |  | Coleoptera |  | Cucujidae |  | C60 | Predator | 3 |
| Insecta |  | Coleoptera |  | Cucujidae |  | LA25 | Predator | 6 |
| Insecta |  | Coleoptera |  | Cucujidae |  | LA26 | Predator | 1 |
| Insecta |  | Coleoptera |  | Cucujidae |  | LA37 | Predator | 1 |
| Insecta |  | Coleoptera |  | Cucujidae |  | LA38 | Predator | 4 |
| Insecta |  | Coleoptera |  | Cucujidae |  | LA64 | Predator | 1 |
| Insecta |  | Coleoptera |  | Cucujidae |  | LA70 | Predator | 1 |
| Insecta |  | Coleoptera |  | Curculionidae |  | C102 | Herbivore | 3 |
| Insecta |  | Coleoptera |  | Curculionidae |  | C106 | Herbivore | 1 |
| Insecta |  | Coleoptera |  | Curculionidae |  | C112 | Herbivore | 1 |
| Insecta |  | Coleoptera |  | Curculionidae |  | C113 | Herbivore | 4 |
| Insecta |  | Coleoptera |  | Curculionidae |  | C116 | Herbivore | 6 |
| Insecta |  | Coleoptera |  | Curculionidae |  | C144 | Herbivore | 10 |
| Insecta |  | Coleoptera |  | Curculionidae |  | C161 | Herbivore | 29 |
| Insecta |  | Coleoptera |  | Curculionidae |  | C162 | Herbivore | 5 |
| Insecta |  | Coleoptera |  | Curculionidae |  | C163 | Herbivore | 3 |
| Insecta |  | Coleoptera |  | Curculionidae |  | C169 | Herbivore | 1 |
| Insecta |  | Coleoptera |  | Curculionidae |  | C177 | Herbivore | 6 |
| Insecta |  | Coleoptera |  | Curculionidae |  | C188 | Herbivore | 4 |
| Insecta |  | Coleoptera |  | Curculionidae |  | C211 | Herbivore | 2 |
| Insecta |  | Coleoptera |  | Curculionidae |  | C218 | Herbivore | 3 |
| Insecta |  | Coleoptera |  | Curculionidae |  | C219 | Herbivore | 43 |
| Insecta |  | Coleoptera |  | Curculionidae |  | C227 | Herbivore | 1 |
| Insecta |  | Coleoptera |  | Curculionidae |  | C233 | Herbivore | 1 |
| Insecta |  | Coleoptera |  | Curculionidae |  | C255 | Herbivore | 2 |
| Insecta |  | Coleoptera |  | Curculionidae |  | C262 | Herbivore | 2 |
| Insecta |  | Coleoptera |  | Curculionidae |  | C266 | Herbivore | 2 |
| Insecta |  | Coleoptera |  | Curculionidae |  | C28 | Herbivore | 67 |
| Insecta |  | Coleoptera |  | Curculionidae |  | C286 | Herbivore | 1 |
| Insecta |  | Coleoptera |  | Curculionidae |  | C289 | Herbivore | 1 |
| Insecta |  | Coleoptera |  | Curculionidae |  | C29 | Herbivore | 36 |
| Insecta |  | Coleoptera |  | Curculionidae |  | C297 | Herbivore | 4 |
| Insecta |  | Coleoptera |  | Curculionidae |  | C3 | Herbivore | 5 |
| Insecta |  | Coleoptera |  | Curculionidae |  | C301 | Herbivore | 1 |
| Insecta |  | Coleoptera |  | Curculionidae |  | C313 | Herbivore | 1 |
| Insecta |  | Coleoptera |  | Curculionidae |  | C319 | Herbivore | 8 |
| Insecta |  | Coleoptera |  | Curculionidae |  | C330 | Herbivore | 1 |
| Insecta |  | Coleoptera |  | Curculionidae |  | C335 | Herbivore | 1 |
| Insecta |  | Coleoptera |  | Curculionidae |  | C342 | Herbivore | 2 |
| Insecta |  | Coleoptera |  | Curculionidae |  | C345 | Herbivore | 1 |
| Insecta |  | Coleoptera |  | Curculionidae |  | C349 | Herbivore | 1 |
| Insecta |  | Coleoptera |  | Curculionidae |  | C5 | Herbivore | 1316 |
| Insecta |  | Coleoptera |  | Curculionidae |  | C57 | Herbivore | 470 |
| Insecta |  | Coleoptera |  | Curculionidae |  | C70 | Herbivore | 12 |
| Insecta |  | Coleoptera |  | Curculionidae |  | C73 | Herbivore | 1 |
| Insecta |  | Coleoptera |  | Curculionidae |  | C78 | Herbivore | 3 |
| Insecta |  | Coleoptera |  | Curculionidae |  | C82 | Herbivore | 72 |
| Insecta |  | Coleoptera |  | Curculionidae |  | C86 | Herbivore | 2 |
| Insecta |  | Coleoptera |  | Curculionidae |  | C91 | Herbivore | 3 |
| Insecta |  | Coleoptera |  | Curculionidae |  | C98 | Herbivore | 59 |
| Insecta |  | Coleoptera |  | Curculionidae |  | C99 | Herbivore | 7 |
| Insecta |  | Coleoptera |  | Curculionidae |  | LA60 | Herbivore | 2 |
| Insecta |  | Coleoptera |  | Curculionidae |  | LA65 | Herbivore | 1 |
| Insecta |  | Coleoptera |  | Discolomidae |  | C108 | Detritivore | 2 |
| Insecta |  | Coleoptera |  | Discolomidae |  | C199 | Detritivore | 3 |
| Insecta |  | Coleoptera |  | Discolomidae |  | C239 | Detritivore | 1 |
| Insecta |  | Coleoptera |  | Elateridae |  | C318 | Herbivore | 1 |
| Insecta |  | Coleoptera |  | Elateridae |  | C6 | Herbivore | 6 |
| Insecta |  | Coleoptera |  | Elateridae |  | C71 | Herbivore | 28 |
| Insecta |  | Coleoptera |  | Eucnemidae |  | C317 | Detritivore | 1 |
| Insecta |  | Coleoptera |  | Hydrochidae |  | C51 | Detritivore | 1 |
| Insecta |  | Coleoptera |  | Lampyridae |  | C248 | Predator | 5 |
| Insecta |  | Coleoptera |  | Lampyridae |  | C279 | Predator | 1 |
| Insecta |  | Coleoptera |  | Lampyridae |  | C280 | Predator | 3 |
| Insecta |  | Coleoptera |  | Lampyridae |  | LA32 | Predator | 1 |
| Insecta |  | Coleoptera |  | Lampyridae |  | LA4 | Predator | 2 |
| Insecta |  | Coleoptera |  | Languridae |  | C170 | Herbivore | 1 |
| Insecta |  | Coleoptera |  | Languriidae |  | C264 | Herbivore | 1 |
| Insecta |  | Coleoptera |  | Lyctidae |  | C2 | Herbivore | 2 |
| Insecta |  | Coleoptera |  | Meloidae |  | C290 | Herbivore | 5 |
| Insecta |  | Coleoptera |  | Meloidae |  | LA55 | Predator | 1 |
| Insecta |  | Coleoptera |  | Melyridae |  | C121 | Predator | 6 |
| Insecta |  | Coleoptera |  | Melyridae |  | C166 | Predator | 3 |
| Insecta |  | Coleoptera |  | Melyridae |  | C295 | Predator | 1 |
| Insecta |  | Coleoptera |  | Mordellidae |  | C168 | Herbivore | 2 |
| Insecta |  | Coleoptera |  | Mordellidae |  | C20 | Herbivore | 3 |
| Insecta |  | Coleoptera |  | Mordellidae |  | C212 | Herbivore | 1 |
| Insecta |  | Coleoptera |  | Mordellidae |  | C96 | Herbivore | 1 |
| Insecta |  | Coleoptera |  | Nitidulidae |  | C126 | Detritivore | 3 |
| Insecta |  | Coleoptera |  | Nitidulidae |  | C14 | Detritivore | 19 |
| Insecta |  | Coleoptera |  | Nitidulidae |  | C141 | Detritivore | 1 |
| Insecta |  | Coleoptera |  | Nitidulidae |  | C145 | Detritivore | 3 |
| Insecta |  | Coleoptera |  | Nitidulidae |  | C155 | Detritivore | 3 |
| Insecta |  | Coleoptera |  | Nitidulidae |  | C256 | Detritivore | 1 |
| Insecta |  | Coleoptera |  | Nitidulidae |  | C268 | Detritivore | 1 |
| Insecta |  | Coleoptera |  | Nitidulidae |  | C309 | Detritivore | 1 |
| Insecta |  | Coleoptera |  | Nitidulidae |  | C32 | Detritivore | 22 |
| Insecta |  | Coleoptera |  | Nitidulidae |  | C41 | Detritivore | 2 |
| Insecta |  | Coleoptera |  | Nitidulidae |  | C67 | Detritivore | 3 |
| Insecta |  | Coleoptera |  | Oedemeridae |  | C296 | Herbivore | 32 |
| Insecta |  | Coleoptera |  | Phalacridae |  | C132 | Detritivore | 1 |
| Insecta |  | Coleoptera |  | Phalacridae |  | C243 | Detritivore | 1 |
| Insecta |  | Coleoptera |  | Phalacridae |  | C269 | Detritivore | 1 |
| Insecta |  | Coleoptera |  | Phalacridae |  | C74 | Detritivore | 3 |
| Insecta |  | Coleoptera |  | Phalacridae |  | C77 | Detritivore | 1 |
| Insecta |  | Coleoptera |  | Ptiliidae |  | C149 | Detritivore | 14 |
| Insecta |  | Coleoptera |  | Ripiphoridae |  | C135 | Predator | 2 |
| Insecta |  | Coleoptera |  | Salpingidae |  | C291 | Detritivore | 3 |
| Insecta |  | Coleoptera |  | Salpingidae |  | C324 | Detritivore | 1 |
| Insecta |  | Coleoptera |  | Salpingidae |  | C34 | Detritivore | 2 |
| Insecta |  | Coleoptera |  | Salpingidae |  | C42 | Detritivore | 2 |
| Insecta |  | Coleoptera |  | Scarabaeidae |  | C234 | Detritivore | 1 |
| Insecta |  | Coleoptera |  | Scarabaeidae |  | C278 | Detritivore | 4 |
| Insecta |  | Coleoptera |  | Scolytinae |  | C114 | Herbivore | 3 |
| Insecta |  | Coleoptera |  | Scolytinae |  | C236 | Herbivore | 1 |
| Insecta |  | Coleoptera |  | Scolytinae |  | C321 | Herbivore | 1 |
| Insecta |  | Coleoptera |  | Scraptiidae |  | C101 | Herbivore | 1 |
| Insecta |  | Coleoptera |  | Scraptiidae |  | C105b | Herbivore | 1 |
| Insecta |  | Coleoptera |  | Scraptiidae |  | C109 | Herbivore | 11 |
| Insecta |  | Coleoptera |  | Scraptiidae |  | C133 | Herbivore | 6 |
| Insecta |  | Coleoptera |  | Scraptiidae |  | C194 | Herbivore | 2 |
| Insecta |  | Coleoptera |  | Scraptiidae |  | C221 | Herbivore | 4 |
| Insecta |  | Coleoptera |  | Scraptiidae |  | C252 | Herbivore | 1 |
| Insecta |  | Coleoptera |  | Scraptiidae |  | C259 | Herbivore | 1 |
| Insecta |  | Coleoptera |  | Scraptiidae |  | C272 | Herbivore | 1 |
| Insecta |  | Coleoptera |  | Scraptiidae |  | C276 | Herbivore | 1 |
| Insecta |  | Coleoptera |  | Scraptiidae |  | C340 | Herbivore | 3 |
| Insecta |  | Coleoptera |  | Scraptiidae |  | C351 | Herbivore | 7 |
| Insecta |  | Coleoptera |  | Scraptiidae |  | C75 | Herbivore | 41 |
| Insecta |  | Coleoptera |  | Scraptiidae |  | C76 | Herbivore | 1 |
| Insecta |  | Coleoptera |  | Scydmaenidae |  | C160 | Predator | 1 |
| Insecta |  | Coleoptera |  | Silvanidae |  | C156 | Detritivore | 7 |
| Insecta |  | Coleoptera |  | Silvanidae |  | C184 | Detritivore | 1 |
| Insecta |  | Coleoptera |  | Silvanidae |  | C210 | Detritivore | 2 |
| Insecta |  | Coleoptera |  | Silvanidae |  | C237 | Detritivore | 1 |
| Insecta |  | Coleoptera |  | Silvanidae |  | C27 | Detritivore | 7 |
| Insecta |  | Coleoptera |  | Silvanidae |  | C285 | Detritivore | 1 |
| Insecta |  | Coleoptera |  | Silvanidae |  | C314 | Detritivore | 1 |
| Insecta |  | Coleoptera |  | Silvanidae |  | C320 | Detritivore | 1 |
| Insecta |  | Coleoptera |  | Silvanidae |  | C333 | Detritivore | 1 |
| Insecta |  | Coleoptera |  | Silvanidae |  | C339 | Detritivore | 2 |
| Insecta |  | Coleoptera |  | Silvanidae |  | C50 | Detritivore | 35 |
| Insecta |  | Coleoptera |  | Staphylinidae |  | C107 | Predator | 2 |
| Insecta |  | Coleoptera |  | Staphylinidae |  | C124 | Predator | 9 |
| Insecta |  | Coleoptera |  | Staphylinidae |  | C131 | Predator | 13 |
| Insecta |  | Coleoptera |  | Staphylinidae |  | C137 | Predator | 26 |
| Insecta |  | Coleoptera |  | Staphylinidae |  | C138 | Predator | 1 |
| Insecta |  | Coleoptera |  | Staphylinidae |  | C146 | Predator | 1 |
| Insecta |  | Coleoptera |  | Staphylinidae |  | C171 | Predator | 4 |
| Insecta |  | Coleoptera |  | Staphylinidae |  | C186 | Predator | 2 |
| Insecta |  | Coleoptera |  | Staphylinidae |  | C187 | Predator | 2 |
| Insecta |  | Coleoptera |  | Staphylinidae |  | C190 | Predator | 19 |
| Insecta |  | Coleoptera |  | Staphylinidae |  | C205 | Predator | 1 |
| Insecta |  | Coleoptera |  | Staphylinidae |  | C216 | Predator | 1 |
| Insecta |  | Coleoptera |  | Staphylinidae |  | C226 | Predator | 2 |
| Insecta |  | Coleoptera |  | Staphylinidae |  | C240 | Predator | 1 |
| Insecta |  | Coleoptera |  | Staphylinidae |  | C245 | Predator | 1 |
| Insecta |  | Coleoptera |  | Staphylinidae |  | C246 | Predator | 2 |
| Insecta |  | Coleoptera |  | Staphylinidae |  | C260 | Predator | 1 |
| Insecta |  | Coleoptera |  | Staphylinidae |  | C270 | Predator | 1 |
| Insecta |  | Coleoptera |  | Staphylinidae |  | C271 | Predator | 1 |
| Insecta |  | Coleoptera |  | Staphylinidae |  | C275 | Predator | 1 |
| Insecta |  | Coleoptera |  | Staphylinidae |  | C284 | Predator | 1 |
| Insecta |  | Coleoptera |  | Staphylinidae |  | C288 | Predator | 6 |
| Insecta |  | Coleoptera |  | Staphylinidae |  | C300 | Predator | 2 |
| Insecta |  | Coleoptera |  | Staphylinidae |  | C305 | Predator | 1 |
| Insecta |  | Coleoptera |  | Staphylinidae |  | C308 | Predator | 1 |
| Insecta |  | Coleoptera |  | Staphylinidae |  | C311 | Predator | 1 |
| Insecta |  | Coleoptera |  | Staphylinidae |  | C337 | Predator | 1 |
| Insecta |  | Coleoptera |  | Staphylinidae |  | C43 | Predator | 107 |
| Insecta |  | Coleoptera |  | Staphylinidae |  | C56 | Predator | 23 |
| Insecta |  | Coleoptera |  | Staphylinidae |  | C69 | Predator | 13 |
| Insecta |  | Coleoptera |  | Staphylinidae |  | C79 | Predator | 7 |
| Insecta |  | Coleoptera |  | Tenebrionidae |  | C13 | Detritivore | 1 |
| Insecta |  | Coleoptera |  | Tenebrionidae |  | C151 | Detritivore | 1 |
| Insecta |  | Coleoptera |  | Tenebrionidae |  | C228 | Detritivore | 4 |
| Insecta |  | Coleoptera |  | Tenebrionidae |  | C347 | Detritivore | 1 |
| Insecta |  | Coleoptera |  | Tenebrionidae |  | C36 | Detritivore | 1 |
| Insecta |  | Coleoptera |  | Tenebrionidae |  | C84 | Detritivore | 28 |
| Insecta |  | Coleoptera |  | Tenebrionidae |  | LA11 | Detritivore | 9 |
| Insecta |  | Dermaptera |  | Forficulidae |  | V1 | Detritivore | 49 |
| Insecta |  | Dermaptera |  | Forficulidae |  | V2 | Detritivore | 9 |
| Insecta |  | Diptera |  | Anthomyiidae |  | D107 | Tourist | 1 |
| Insecta |  | Diptera |  | Anthomyiidae |  | D4 | Tourist | 25 |
| Insecta |  | Diptera |  | Asilidae |  | D203 | Predator | 1 |
| Insecta |  | Diptera |  | Asilidae |  | D213 | Predator | 1 |
| Insecta |  | Diptera |  | Asilidae |  | D216 | Predator | 1 |
| Insecta |  | Diptera |  | Asilidae |  | D77 | Predator | 2 |
| Insecta |  | Diptera |  | Asteiidae |  | D3 | Tourist | 92 |
| Insecta |  | Diptera |  | Aulacigastridae |  | D7 | Herbivore | 108 |
| Insecta |  | Diptera |  | Calliphoridae |  | D11 | Pollinator | 6 |
| Insecta |  | Diptera |  | Calliphoridae |  | D12 | Pollinator | 2 |
| Insecta |  | Diptera |  | Calliphoridae |  | D13 | Pollinator | 8 |
| Insecta |  | Diptera |  | Calliphoridae |  | D156 | Pollinator | 2 |
| Insecta |  | Diptera |  | Calliphoridae |  | D158 | Pollinator | 1 |
| Insecta |  | Diptera |  | Calliphoridae |  | D167 | Pollinator | 1 |
| Insecta |  | Diptera |  | Calliphoridae |  | D17 | Pollinator | 4 |
| Insecta |  | Diptera |  | Calliphoridae |  | D176 | Pollinator | 2 |
| Insecta |  | Diptera |  | Calliphoridae |  | D178 | Pollinator | 3 |
| Insecta |  | Diptera |  | Calliphoridae |  | D184 | Pollinator | 2 |
| Insecta |  | Diptera |  | Calliphoridae |  | D185 | Pollinator | 1 |
| Insecta |  | Diptera |  | Calliphoridae |  | D30 | Pollinator | 5 |
| Insecta |  | Diptera |  | Calliphoridae |  | D75 | Pollinator | 1 |
| Insecta |  | Diptera |  | Calliphoridae |  | D78 | Pollinator | 2 |
| Insecta |  | Diptera |  | Cecidomyiidae |  | D121 | Tourist | 1 |
| Insecta |  | Diptera |  | Cecidomyiidae |  | D130 | Tourist | 1 |
| Insecta |  | Diptera |  | Cecidomyiidae |  | D143 | Tourist | 1 |
| Insecta |  | Diptera |  | Cecidomyiidae |  | D146 | Tourist | 2 |
| Insecta |  | Diptera |  | Cecidomyiidae |  | D150 | Tourist | 93 |
| Insecta |  | Diptera |  | Cecidomyiidae |  | D159 | Tourist | 4 |
| Insecta |  | Diptera |  | Cecidomyiidae |  | D180 | Tourist | 1 |
| Insecta |  | Diptera |  | Cecidomyiidae |  | D23 | Tourist | 11 |
| Insecta |  | Diptera |  | Ceratopogonidae |  | D140 | Tourist | 2 |
| Insecta |  | Diptera |  | Ceratopogonidae |  | D197 | Tourist | 1 |
| Insecta |  | Diptera |  | Ceratopogonidae |  | D84 | Tourist | 2 |
| Insecta |  | Diptera |  | Chamaemyiidae |  | D22 | Predator | 2 |
| Insecta |  | Diptera |  | Chaoboridae |  | D154 | Tourist | 1 |
| Insecta |  | Diptera |  | Chaoboridae |  | D31 | Tourist | 58 |
| Insecta |  | Diptera |  | Chaoboridae |  | D67 | Tourist | 5 |
| Insecta |  | Diptera |  | Chironomidae |  | D153 | Tourist | 3 |
| Insecta |  | Diptera |  | Chironomidae |  | D191 | Tourist | 1 |
| Insecta |  | Diptera |  | Chironomidae |  | D196 | Tourist | 3 |
| Insecta |  | Diptera |  | Chironomidae |  | D202 | Tourist | 1 |
| Insecta |  | Diptera |  | Chironomidae |  | D205 | Tourist | 1 |
| Insecta |  | Diptera |  | Chironomidae |  | D29 | Tourist | 95 |
| Insecta |  | Diptera |  | Chloropidae |  | D148 | Tourist | 1 |
| Insecta |  | Diptera |  | Chloropidae |  | D82 | Tourist | 1 |
| Insecta |  | Diptera |  | Chloropidae |  | D87 | Tourist | 3 |
| Insecta |  | Diptera |  | Chloropidae |  | D97 | Tourist | 5 |
| Insecta |  | Diptera |  | Clusiidae |  | D104 | Detritivore | 1 |
| Insecta |  | Diptera |  | Clusiidae |  | D149 | Detritivore | 1 |
| Insecta |  | Diptera |  | Clusiidae |  | D177 | Detritivore | 4 |
| Insecta |  | Diptera |  | Clusiidae |  | D187 | Detritivore | 1 |
| Insecta |  | Diptera |  | Clusiidae |  | D188 | Detritivore | 4 |
| Insecta |  | Diptera |  | Clusiidae |  | D210 | Detritivore | 1 |
| Insecta |  | Diptera |  | Clusiidae |  | D211 | Detritivore | 2 |
| Insecta |  | Diptera |  | Culicidae |  | D14 | Tourist | 6 |
| Insecta |  | Diptera |  | Culicidae |  | D168 | Tourist | 1 |
| Insecta |  | Diptera |  | Culicidae |  | D20 | Tourist | 6 |
| Insecta |  | Diptera |  | Culicidae |  | D201 | Tourist | 1 |
| Insecta |  | Diptera |  | Culicidae |  | D40 | Tourist | 1 |
| Insecta |  | Diptera |  | Culicidae |  | D41 | Tourist | 3 |
| Insecta |  | Diptera |  | Culicidae |  | D76 | Tourist | 2 |
| Insecta |  | Diptera |  | Culicidae |  | D8 | Tourist | 8 |
| Insecta |  | Diptera |  | Curtonotidae |  | D95 | Tourist | 2 |
| Insecta |  | Diptera |  | Diastatidae |  | D80 | Tourist | 1 |
| Insecta |  | Diptera |  | Diastatidae |  | D85 | Tourist | 2 |
| Insecta |  | Diptera |  | Dixidae |  | D32 | Tourist | 14 |
| Insecta |  | Diptera |  | Dolichopodidae |  | D113 | Predator | 2 |
| Insecta |  | Diptera |  | Drosophilidae |  | D109 | Detritivore | 2 |
| Insecta |  | Diptera |  | Drosophilidae |  | D141 | Detritivore | 7 |
| Insecta |  | Diptera |  | Empididae |  | D207 | Predator | 1 |
| Insecta |  | Diptera |  | Ephydridae |  | D123 | Tourist | 10 |
| Insecta |  | Diptera |  | Ephydridae |  | D145 | Tourist | 1 |
| Insecta |  | Diptera |  | Ephydridae |  | D96 | Tourist | 2 |
| Insecta |  | Diptera |  | Fanniidae |  | D115 | Tourist | 4 |
| Insecta |  | Diptera |  | Fanniidae |  | D170 | Tourist | 1 |
| Insecta |  | Diptera |  | Heleomyzidae |  | D102 | Tourist | 10 |
| Insecta |  | Diptera |  | Heleomyzidae |  | D189 | Tourist | 2 |
| Insecta |  | Diptera |  | Lauxaniidae |  | D106 | Tourist | 1 |
| Insecta |  | Diptera |  | Lauxaniidae |  | D173 | Tourist | 1 |
| Insecta |  | Diptera |  | Lauxaniidae |  | D18 | Tourist | 1 |
| Insecta |  | Diptera |  | Lauxaniidae |  | D91 | Tourist | 2 |
| Insecta |  | Diptera |  | Lonchaeidae |  | D157 | Tourist | 1 |
| Insecta |  | Diptera |  | Lonchaeidae |  | D198 | Tourist | 2 |
| Insecta |  | Diptera |  | Lonchaeidae |  | D200 | Tourist | 1 |
| Insecta |  | Diptera |  | Lonchaeidae |  | D36 | Tourist | 118 |
| Insecta |  | Diptera |  | Lonchaeidae |  | D81 | Tourist | 8 |
| Insecta |  | Diptera |  | Milichiidae |  | D128 | Tourist | 6 |
| Insecta |  | Diptera |  | Milichiidae |  | D93 | Tourist | 3 |
| Insecta |  | Diptera |  | Muscidae |  | D120 | Tourist | 9 |
| Insecta |  | Diptera |  | Muscidae |  | D15 | Tourist | 5 |
| Insecta |  | Diptera |  | Muscidae |  | D162 | Tourist | 1 |
| Insecta |  | Diptera |  | Muscidae |  | D174 | Tourist | 1 |
| Insecta |  | Diptera |  | Muscidae |  | D179 | Tourist | 4 |
| Insecta |  | Diptera |  | Muscidae |  | D21 | Tourist | 12 |
| Insecta |  | Diptera |  | Muscidae |  | D42 | Tourist | 6 |
| Insecta |  | Diptera |  | Muscidae |  | D71 | Tourist | 1 |
| Insecta |  | Diptera |  | Muscidae |  | D99 | Tourist | 4 |
| Insecta |  | Diptera |  | Mycetophilidae |  | D135 | Detritivore | 1 |
| Insecta |  | Diptera |  | Mycetophilidae |  | D138 | Detritivore | 4 |
| Insecta |  | Diptera |  | Mycetophilidae |  | D139 | Detritivore | 1 |
| Insecta |  | Diptera |  | Mycetophilidae |  | D151 | Detritivore | 5 |
| Insecta |  | Diptera |  | Mycetophilidae |  | D164 | Detritivore | 1 |
| Insecta |  | Diptera |  | Mycetophilidae |  | D192 | Detritivore | 1 |
| Insecta |  | Diptera |  | Mycetophilidae |  | D44 | Detritivore | 216 |
| Insecta |  | Diptera |  | Mycetophilidae |  | D45 | Detritivore | 37 |
| Insecta |  | Diptera |  | Mycetophilidae |  | D52 | Detritivore | 5 |
| Insecta |  | Diptera |  | Mycetophilidae |  | D53 | Detritivore | 4 |
| Insecta |  | Diptera |  | Mycetophilidae |  | D60 | Detritivore | 6 |
| Insecta |  | Diptera |  | Mycetophilidae |  | D61 | Detritivore | 1 |
| Insecta |  | Diptera |  | Mycetophilidae |  | D63 | Detritivore | 1 |
| Insecta |  | Diptera |  | Mycetophilidae |  | D64 | Detritivore | 31 |
| Insecta |  | Diptera |  | Mycetophilidae |  | D69 | Detritivore | 9 |
| Insecta |  | Diptera |  | Odiniidae |  | D110 | Tourist | 1 |
| Insecta |  | Diptera |  | Phoridae |  | D100 | Tourist | 32 |
| Insecta |  | Diptera |  | Phoridae |  | D108 | Tourist | 1 |
| Insecta |  | Diptera |  | Phoridae |  | D112 | Tourist | 1 |
| Insecta |  | Diptera |  | Phoridae |  | D126 | Tourist | 4 |
| Insecta |  | Diptera |  | Phoridae |  | D182 | Tourist | 1 |
| Insecta |  | Diptera |  | Phoridae |  | D204 | Tourist | 1 |
| Insecta |  | Diptera |  | Phoridae |  | D215 | Tourist | 1 |
| Insecta |  | Diptera |  | Phoridae |  | D37 | Tourist | 12 |
| Insecta |  | Diptera |  | Phoridae |  | D43 | Tourist | 19 |
| Insecta |  | Diptera |  | Phoridae |  | D54 | Tourist | 1 |
| Insecta |  | Diptera |  | Phoridae |  | D55 | Tourist | 1 |
| Insecta |  | Diptera |  | Phoridae |  | D62 | Tourist | 1 |
| Insecta |  | Diptera |  | Phoridae |  | D66 | Tourist | 6 |
| Insecta |  | Diptera |  | Phoridae |  | D73 | Tourist | 8 |
| Insecta |  | Diptera |  | Phoridae |  | D98 | Tourist | 1 |
| Insecta |  | Diptera |  | Pipunculidae |  | D103 | Predator | 1 |
| Insecta |  | Diptera |  | Pipunculidae |  | D152 | Predator | 1 |
| Insecta |  | Diptera |  | Pipunculidae |  | D16 | Predator | 15 |
| Insecta |  | Diptera |  | Pipunculidae |  | D163 | Predator | 1 |
| Insecta |  | Diptera |  | Pipunculidae |  | D72 | Predator | 3 |
| Insecta |  | Diptera |  | Psilidae |  | D118 | Tourist | 13 |
| Insecta |  | Diptera |  | Psilidae |  | D193 | Tourist | 1 |
| Insecta |  | Diptera |  | Psilidae |  | D88 | Tourist | 6 |
| Insecta |  | Diptera |  | Ptychopteridae |  | D136 | Tourist | 1 |
| Insecta |  | Diptera |  | Pyrgotidae |  | D79 | Parasite | 2 |
| Insecta |  | Diptera |  | Pyrgotidae |  | D94 | Parasite | 1 |
| Insecta |  | Diptera |  | Rhagionidae |  | D125 | Tourist | 1 |
| Insecta |  | Diptera |  | Rhinophoridae |  | D117 | Predator | 1 |
| Insecta |  | Diptera |  | Rhinophoridae |  | D127 | Predator | 8 |
| Insecta |  | Diptera |  | Rhinophoridae |  | D134 | Predator | 4 |
| Insecta |  | Diptera |  | Rhinophoridae |  | D169 | Predator | 1 |
| Insecta |  | Diptera |  | Rhinophoridae |  | D186 | Predator | 8 |
| Insecta |  | Diptera |  | Rhinophoridae |  | D206 | Predator | 1 |
| Insecta |  | Diptera |  | Rhinophoridae |  | D33 | Predator | 3 |
| Insecta |  | Diptera |  | Rhinophoridae |  | D92 | Predator | 18 |
| Insecta |  | Diptera |  | Scatopsidae |  | D1 | Tourist | 95 |
| Insecta |  | Diptera |  | Sciaridae |  | D183 | Detritivore | 1 |
| Insecta |  | Diptera |  | Sciaridae |  | D28 | Detritivore | 850 |
| Insecta |  | Diptera |  | Sciaridae |  | D59 | Detritivore | 1 |
| Insecta |  | Diptera |  | Sciomyzidae |  | D155 | Tourist | 1 |
| Insecta |  | Diptera |  | Sciomyzidae |  | D214 | Tourist | 1 |
| Insecta |  | Diptera |  | Sciomyzidae |  | D51 | Tourist | 32 |
| Insecta |  | Diptera |  | Simuliidae |  | D166 | Tourist | 2 |
| Insecta |  | Diptera |  | Simuliidae |  | D26 | Tourist | 1 |
| Insecta |  | Diptera |  | Stratiomyidae |  | D137 | Tourist | 1 |
| Insecta |  | Diptera |  | Syrphidae |  | D119 | Tourist | 1 |
| Insecta |  | Diptera |  | Syrphidae |  | D160 | Tourist | 1 |
| Insecta |  | Diptera |  | Syrphidae |  | D2 | Tourist | 3 |
| Insecta |  | Diptera |  | Syrphidae |  | D38 | Tourist | 3 |
| Insecta |  | Diptera |  | Syrphidae |  | D65 | Tourist | 1 |
| Insecta |  | Diptera |  | Tachinidae |  | D161 | Predator | 1 |
| Insecta |  | Diptera |  | Tachinidae |  | D175 | Predator | 1 |
| Insecta |  | Diptera |  | Tachinidae |  | D181 | Predator | 1 |
| Insecta |  | Diptera |  | Tachinidae |  | D194 | Predator | 1 |
| Insecta |  | Diptera |  | Tachinidae |  | D199 | Predator | 1 |
| Insecta |  | Diptera |  | Tachinidae |  | D25 | Predator | 1 |
| Insecta |  | Diptera |  | Tachinidae |  | D34 | Predator | 3 |
| Insecta |  | Diptera |  | Tachinidae |  | D70 | Predator | 1 |
| Insecta |  | Diptera |  | Tachinidae |  | D74 | Predator | 1 |
| Insecta |  | Diptera |  | Tachinidae |  | D89 | Predator | 1 |
| Insecta |  | Diptera |  | Tachinidae |  | D9 | Predator | 34 |
| Insecta |  | Diptera |  | Tachinidae |  | D90 | Predator | 9 |
| Insecta |  | Diptera |  | Tephritidae |  | D19 | Tourist | 18 |
| Insecta |  | Diptera |  | Tephritidae |  | D114 | Tourist | 3 |
| Insecta |  | Diptera |  | Tephritidae |  | D116 | Tourist | 1 |
| Insecta |  | Diptera |  | Tephritidae |  | D129 | Tourist | 17 |
| Insecta |  | Diptera |  | Tephritidae |  | D131 | Tourist | 3 |
| Insecta |  | Diptera |  | Tephritidae |  | D133 | Tourist | 1 |
| Insecta |  | Diptera |  | Tephritidae |  | D147 | Tourist | 2 |
| Insecta |  | Diptera |  | Tephritidae |  | D165 | Tourist | 3 |
| Insecta |  | Diptera |  | Tephritidae |  | D190 | Tourist | 1 |
| Insecta |  | Diptera |  | Tephritidae |  | D24 | Tourist | 9 |
| Insecta |  | Diptera |  | Tephritidae |  | D35 | Tourist | 17 |
| Insecta |  | Diptera |  | Tephritidae |  | D39 | Tourist | 1 |
| Insecta |  | Diptera |  | Tephritidae |  | D46 | Tourist | 2 |
| Insecta |  | Diptera |  | Tephritidae |  | D50 | Tourist | 2 |
| Insecta |  | Diptera |  | Tephritidae |  | D86 | Tourist | 2 |
| Insecta |  | Diptera |  | Therevidae |  | D172 | Herbivore | 2 |
| Insecta |  | Diptera |  | Therevidae |  | D56 | Herbivore | 6 |
| Insecta |  | Diptera |  | Tipulidae |  | D10 | Tourist | 48 |
| Insecta |  | Diptera |  | Tipulidae |  | D101 | Tourist | 20 |
| Insecta |  | Diptera |  | Tipulidae |  | D122 | Tourist | 6 |
| Insecta |  | Diptera |  | Tipulidae |  | D124 | Tourist | 1 |
| Insecta |  | Diptera |  | Tipulidae |  | D142 | Tourist | 3 |
| Insecta |  | Diptera |  | Tipulidae |  | D144 | Tourist | 3 |
| Insecta |  | Diptera |  | Tipulidae |  | D171 | Tourist | 1 |
| Insecta |  | Diptera |  | Tipulidae |  | D209 | Tourist | 1 |
| Insecta |  | Diptera |  | Tipulidae |  | D212 | Tourist | 1 |
| Insecta |  | Diptera |  | Tipulidae |  | D47 | Tourist | 21 |
| Insecta |  | Diptera |  | Tipulidae |  | D48 | Tourist | 16 |
| Insecta |  | Diptera |  | Tipulidae |  | D49 | Tourist | 10 |
| Insecta |  | Diptera |  | Tipulidae |  | D57 | Tourist | 1 |
| Insecta |  | Diptera |  | Xylomyidae |  | D132 | Herbivore | 1 |
| Insecta |  | Diptera |  |  |  | LA10 | Detritivore | 19 |
| Insecta |  | Diptera |  |  |  | LA28 | Detritivore | 2 |
| Insecta |  | Diptera |  |  |  | LA30 | Detritivore | 6 |
| Insecta |  | Diptera |  |  |  | LA51 | Detritivore | 1 |
| Insecta |  | Diptera |  |  |  | LA54 | Detritivore | 44 |
| Insecta |  | Diptera |  |  |  | LA61 | Detritivore | 1 |
| Insecta |  | Diptera |  |  |  | LA63 | Detritivore | 1 |
| Insecta |  | Ephemeroptera |  |  |  | E2 | Tourist | 4 |
| Insecta |  | Ephemeroptera |  |  |  | N12 | Tourist | 1 |
| Insecta |  | Hemiptera |  | Achilidae |  | HE133 | Herbivore | 2 |
| Insecta |  | Hemiptera |  | Aphrophoridae |  | HE124 | Herbivore | 1 |
| Insecta |  | Hemiptera |  | Aphrophoridae |  | HE70 | Herbivore | 12 |
| Insecta |  | Hemiptera |  | Aphrophoridae |  | HE88 | Herbivore | 4 |
| Insecta |  | Hemiptera |  | Asopinae |  | HE75 | Predator | 1 |
| Insecta |  | Hemiptera |  | Berytidae |  | HE125 | Herbivore | 1 |
| Insecta |  | Hemiptera |  | Cercopidae |  | HE119 | Herbivore | 1 |
| Insecta |  | Hemiptera |  | Cercopidae |  | HE126 | Herbivore | 1 |
| Insecta |  | Hemiptera |  | Cercopidae |  | HE14 | Herbivore | 4 |
| Insecta |  | Hemiptera |  | Cercopidae |  | HE6 | Herbivore | 58 |
| Insecta |  | Hemiptera |  | Cercopidae |  | HE93 | Herbivore | 1 |
| Insecta |  | Hemiptera |  | Cicadellidae |  | HE102 | Herbivore | 1 |
| Insecta |  | Hemiptera |  | Cicadellidae |  | HE103 | Herbivore | 1 |
| Insecta |  | Hemiptera |  | Cicadellidae |  | HE106 | Herbivore | 6 |
| Insecta |  | Hemiptera |  | Cicadellidae |  | HE108 | Herbivore | 1 |
| Insecta |  | Hemiptera |  | Cicadellidae |  | HE118 | Herbivore | 12 |
| Insecta |  | Hemiptera |  | Cicadellidae |  | HE121 | Herbivore | 10 |
| Insecta |  | Hemiptera |  | Cicadellidae |  | HE123 | Herbivore | 1 |
| Insecta |  | Hemiptera |  | Cicadellidae |  | HE13 | Herbivore | 2 |
| Insecta |  | Hemiptera |  | Cicadellidae |  | HE130 | Herbivore | 1 |
| Insecta |  | Hemiptera |  | Cicadellidae |  | HE131 | Herbivore | 1 |
| Insecta |  | Hemiptera |  | Cicadellidae |  | HE135 | Herbivore | 1 |
| Insecta |  | Hemiptera |  | Cicadellidae |  | HE136 | Herbivore | 1 |
| Insecta |  | Hemiptera |  | Cicadellidae |  | HE139 | Herbivore | 1 |
| Insecta |  | Hemiptera |  | Cicadellidae |  | HE143 | Herbivore | 1 |
| Insecta |  | Hemiptera |  | Cicadellidae |  | HE17 | Herbivore | 504 |
| Insecta |  | Hemiptera |  | Cicadellidae |  | HE24 | Herbivore | 9 |
| Insecta |  | Hemiptera |  | Cicadellidae |  | HE26 | Herbivore | 48 |
| Insecta |  | Hemiptera |  | Cicadellidae |  | HE27 | Herbivore | 4 |
| Insecta |  | Hemiptera |  | Cicadellidae |  | HE30 | Herbivore | 6 |
| Insecta |  | Hemiptera |  | Cicadellidae |  | HE34 | Herbivore | 37 |
| Insecta |  | Hemiptera |  | Cicadellidae |  | HE35 | Herbivore | 11 |
| Insecta |  | Hemiptera |  | Cicadellidae |  | HE38 | Herbivore | 2 |
| Insecta |  | Hemiptera |  | Cicadellidae |  | HE4 | Herbivore | 5 |
| Insecta |  | Hemiptera |  | Cicadellidae |  | HE43 | Herbivore | 24 |
| Insecta |  | Hemiptera |  | Cicadellidae |  | HE51 | Herbivore | 1 |
| Insecta |  | Hemiptera |  | Cicadellidae |  | HE52 | Herbivore | 18 |
| Insecta |  | Hemiptera |  | Cicadellidae |  | HE58 | Herbivore | 3 |
| Insecta |  | Hemiptera |  | Cicadellidae |  | HE61 | Herbivore | 2 |
| Insecta |  | Hemiptera |  | Cicadellidae |  | HE66 | Herbivore | 7 |
| Insecta |  | Hemiptera |  | Cicadellidae |  | HE68 | Herbivore | 2 |
| Insecta |  | Hemiptera |  | Cicadellidae |  | HE69 | Herbivore | 5 |
| Insecta |  | Hemiptera |  | Cicadellidae |  | HE72 | Herbivore | 3 |
| Insecta |  | Hemiptera |  | Cicadellidae |  | HE73 | Herbivore | 1 |
| Insecta |  | Hemiptera |  | Cicadellidae |  | HE79 | Herbivore | 1 |
| Insecta |  | Hemiptera |  | Cicadellidae |  | HE83 | Herbivore | 1 |
| Insecta |  | Hemiptera |  | Cicadellidae |  | HE91 | Herbivore | 6 |
| Insecta |  | Hemiptera |  | Cicadellidae |  | HE92 | Herbivore | 1 |
| Insecta |  | Hemiptera |  | Cicadellidae |  | HE94 | Herbivore | 3 |
| Insecta |  | Hemiptera |  | Cicadellidae |  | HE95 | Herbivore | 2 |
| Insecta |  | Hemiptera |  | Cicadellidae |  | HE97 | Herbivore | 4 |
| Insecta |  | Hemiptera |  | Cicadidae |  | HE105 | Herbivore | 1 |
| Insecta |  | Hemiptera |  | Cicadidae |  | HE132 | Herbivore | 2 |
| Insecta |  | Hemiptera |  | Cixiidae |  | HE31 | Herbivore | 6 |
| Insecta |  | Hemiptera |  | Coccidae |  | HE86 | Herbivore | 2 |
| Insecta |  | Hemiptera |  | Coreidae |  | HE122 | Herbivore | 6 |
| Insecta |  | Hemiptera |  | Delphacidae |  | HE128 | Herbivore | 2 |
| Insecta |  | Hemiptera |  | Delphacidae |  | HE134 | Herbivore | 1 |
| Insecta |  | Hemiptera |  | Delphacidae |  | HE21 | Herbivore | 7 |
| Insecta |  | Hemiptera |  | Delphacidae |  | HE59 | Herbivore | 2 |
| Insecta |  | Hemiptera |  | Dictyopharidae |  | HE116 | Herbivore | 3 |
| Insecta |  | Hemiptera |  | Dictyopharidae |  | HE71 | Herbivore | 4 |
| Insecta |  | Hemiptera |  | Emesinae |  | HE16 | Predator | 35 |
| Insecta |  | Hemiptera |  | Emesinae |  | HE74 | Predator | 1 |
| Insecta |  | Hemiptera |  | Enicocephalidae |  | HE144 | Predator | 2 |
| Insecta |  | Hemiptera |  | Enicocephalidae |  | HE41 | Predator | 7 |
| Insecta |  | Hemiptera |  | Enicocephalidae |  | HE53 | Predator | 1 |
| Insecta |  | Hemiptera |  | Enicocephalidae |  | U5 | Predator | 8 |
| Insecta |  | Hemiptera |  | Flatidae |  | HE120 | Herbivore | 2 |
| Insecta |  | Hemiptera |  | Fulgoridae |  | HE39 | Herbivore | 94 |
| Insecta |  | Hemiptera |  | Fulgoridae |  | HE7 | Herbivore | 1 |
| Insecta |  | Hemiptera |  | Gengidae |  | HE141 | Herbivore | 1 |
| Insecta |  | Hemiptera |  | Gengidae |  | HE45 | Herbivore | 11 |
| Insecta |  | Hemiptera |  | Gengidae |  | HE47 | Herbivore | 3 |
| Insecta |  | Hemiptera |  | Gengidae |  | HE48 | Herbivore | 32 |
| Insecta |  | Hemiptera |  | Gengidae |  | HE62 | Herbivore | 1 |
| Insecta |  | Hemiptera |  | Gengidae |  | HE64 | Herbivore | 15 |
| Insecta |  | Hemiptera |  | Ischnorhynchinae |  | HE112 | Herbivore | 4 |
| Insecta |  | Hemiptera |  | Lygaeidae |  | HE113 | Herbivore | 1 |
| Insecta |  | Hemiptera |  | Lygaeidae |  | HE22 | Herbivore | 3 |
| Insecta |  | Hemiptera |  | Lygaeidae |  | HE23 | Herbivore | 24 |
| Insecta |  | Hemiptera |  | Meenoplidae |  | HE111 | Herbivore | 6 |
| Insecta |  | Hemiptera |  | Meenoplidae |  | HE114 | Herbivore | 2 |
| Insecta |  | Hemiptera |  | Meenoplidae |  | HE99 | Herbivore | 14 |
| Insecta |  | Hemiptera |  | Nabidae |  | HE90 | Predator | 1 |
| Insecta |  | Hemiptera |  | Notonectidae |  | HE76 | Predator | 23 |
| Insecta |  | Hemiptera |  | Pentatomidae |  | HE129 | Herbivore | 1 |
| Insecta |  | Hemiptera |  | Pentatomidae |  | HE5 | Herbivore | 19 |
| Insecta |  | Hemiptera |  | Pentatomidae |  | HE56 | Herbivore | 3 |
| Insecta |  | Hemiptera |  | Pentatomidae |  | HE63 | Herbivore | 1 |
| Insecta |  | Hemiptera |  | Pentatomidae |  | HE81 | Herbivore | 5 |
| Insecta |  | Hemiptera |  | Pentatomidae |  | HE84 | Herbivore | 1 |
| Insecta |  | Hemiptera |  | Pentatomidae |  | HE85 | Herbivore | 6 |
| Insecta |  | Hemiptera |  | Pentatomidae |  | HE96 | Herbivore | 1 |
| Insecta |  | Hemiptera |  | Psyllidae |  | HE110 | Herbivore | 1 |
| Insecta |  | Hemiptera |  | Psyllidae |  | HE137 | Herbivore | 1 |
| Insecta |  | Hemiptera |  | Psyllidae |  | HE140 | Herbivore | 1 |
| Insecta |  | Hemiptera |  | Psyllidae |  | HE19 | Herbivore | 8 |
| Insecta |  | Hemiptera |  | Psyllidae |  | HE20 | Herbivore | 115 |
| Insecta |  | Hemiptera |  | Psyllidae |  | HE28 | Herbivore | 2 |
| Insecta |  | Hemiptera |  | Psyllidae |  | HE3 | Herbivore | 177 |
| Insecta |  | Hemiptera |  | Psyllidae |  | HE44 | Herbivore | 4 |
| Insecta |  | Hemiptera |  | Psyllidae |  | HE54 | Herbivore | 1 |
| Insecta |  | Hemiptera |  | Psyllidae |  | HE67 | Herbivore | 1 |
| Insecta |  | Hemiptera |  | Pyrrhocoridae |  | HE1 | Herbivore | 48 |
| Insecta |  | Hemiptera |  | Pyrrhocoridae |  | HE100 | Herbivore | 3 |
| Insecta |  | Hemiptera |  | Pyrrhocoridae |  | HE101 | Herbivore | 1 |
| Insecta |  | Hemiptera |  | Pyrrhocoridae |  | HE104 | Herbivore | 1 |
| Insecta |  | Hemiptera |  | Pyrrhocoridae |  | HE107 | Herbivore | 2 |
| Insecta |  | Hemiptera |  | Pyrrhocoridae |  | HE11 | Herbivore | 3 |
| Insecta |  | Hemiptera |  | Pyrrhocoridae |  | HE142 | Herbivore | 1 |
| Insecta |  | Hemiptera |  | Pyrrhocoridae |  | HE46 | Herbivore | 1 |
| Insecta |  | Hemiptera |  | Rhyparochromidae |  | HE10 | Herbivore | 6 |
| Insecta |  | Hemiptera |  | Rhyparochromidae |  | HE109 | Herbivore | 1 |
| Insecta |  | Hemiptera |  | Rhyparochromidae |  | HE12 | Herbivore | 3 |
| Insecta |  | Hemiptera |  | Rhyparochromidae |  | HE18 | Herbivore | 25 |
| Insecta |  | Hemiptera |  | Rhyparochromidae |  | HE50 | Herbivore | 140 |
| Insecta |  | Hemiptera |  | Rhyparochromidae |  | HE57 | Herbivore | 1 |
| Insecta |  | Hemiptera |  | Rhyparochromidae |  | HE60 | Herbivore | 3 |
| Insecta |  | Hemiptera |  | Rhyparochromidae |  | HE82 | Herbivore | 1 |
| Insecta |  | Hemiptera |  | Rhyparochromidae |  | HE87 | Herbivore | 2 |
| Insecta |  | Hemiptera |  | Rhyparochromidae |  | HE89 | Herbivore | 1 |
| Insecta |  | Hemiptera |  | Rhyparochromidae |  | HE9 | Herbivore | 3 |
| Insecta |  | Hemiptera |  | Rhyparochromidae |  | HE98 | Herbivore | 1 |
| Insecta |  | Hemiptera |  | Tingidae |  | HE2 | Herbivore | 120 |
| Insecta |  | Hemiptera |  | Tingidae |  | HE8 | Herbivore | 289 |
| Insecta |  | Hymenoptera |  | Formicidae | *Axinidris lignicola* | HY19 | Ant | 53 |
| Insecta |  | Hymenoptera |  | Formicidae | *Camponotus auropubens* | HY503 | Ant | 1 |
| Insecta |  | Hymenoptera |  | Formicidae | *Camponotus maculatus* | HY90 | Ant | 4 |
| Insecta |  | Hymenoptera |  | Formicidae | *Camponotus werthi* | HY517 | Ant | 1 |
| Insecta |  | Hymenoptera |  | Formicidae | *Crematogaster liengmei* | HY66 | Ant | 63 |
| Insecta |  | Hymenoptera |  | Formicidae | *Crematogaster peringueyi* | HY452 | Ant | 21 |
| Insecta |  | Hymenoptera |  | Formicidae | *Formicinae* sp. *2* | HY59 | Ant | 2 |
| Insecta |  | Hymenoptera |  | Formicidae | *Hyponera austra* | HY415 | Ant | 1 |
| Insecta |  | Hymenoptera |  | Formicidae | *Hyponera spei* | HY20 | Ant | 2 |
| Insecta |  | Hymenoptera |  | Formicidae | *Monomorium* sp. *1* | HY21 | Ant | 1 |
| Insecta |  | Hymenoptera |  | Formicidae | *Monomorium* sp. *2* | HY23 | Ant | 275 |
| Insecta |  | Hymenoptera |  | Formicidae | *Myrmecinae* sp. *1* | HY513 | Ant | 4 |
| Insecta |  | Hymenoptera |  | Formicidae | *Myrmecinae* sp. *2* | HY514 | Ant | 1 |
| Insecta |  | Hymenoptera |  | Formicidae | *Myrmecinae* sp. *3* | HY515 | Ant | 7 |
| Insecta |  | Hymenoptera |  | Formicidae | *Myrmecinae* sp. *4* | HY516 | Ant | 1 |
| Insecta |  | Hymenoptera |  | Formicidae | *Myrmecinae* sp. *5* | HY69 | Ant | 2 |
| Insecta |  | Hymenoptera |  | Formicidae | *Nesomyrmex denticulatus* | HY518 | Ant | 50 |
| Insecta |  | Hymenoptera |  | Formicidae | *Pheidole* sp. *1* | HY524 | Ant | 1 |
| Insecta |  | Hymenoptera |  | Formicidae | *Pheidole* sp. *2* | HY22 | Ant | 1 |
| Insecta |  | Hymenoptera |  | Formicidae | *Pheidole* sp. *3* | HY172 | Ant | 2 |
| Insecta |  | Hymenoptera |  | Formicidae | *Plagiolepis brunni* | HY519 | Ant | 167 |
| Insecta |  | Hymenoptera |  | Formicidae | *Plagiolepis decora* | HY124 | Ant | 249 |
| Insecta |  | Hymenoptera |  | Formicidae | *Plagiolepis deweti* | HY300 | Ant | 72 |
| Insecta |  | Hymenoptera |  | Formicidae | *Plagiolepis jouberti* | HY92 | Ant | 27 |
| Insecta |  | Hymenoptera |  | Formicidae | *Plagiolepis puncta* | HY414 | Ant | 1 |
| Insecta |  | Hymenoptera |  | Formicidae | *Plagiolepis* sp. *1* | HY188 | Ant | 18 |
| Insecta |  | Hymenoptera |  | Formicidae | *Plagiolepis* sp. *2* | HY473 | Ant | 1 |
| Insecta |  | Hymenoptera |  | Formicidae | *Polyrhachnis spinicola* | HY123 | Ant | 18 |
| Insecta |  | Hymenoptera |  | Formicidae | *Tapinoma* sp. *1* | HY472 | Ant | 5 |
| Insecta |  | Hymenoptera |  | Formicidae | *Tetramorium capense* | HY455 | Ant | 2 |
| Insecta |  | Hymenoptera |  | Formicidae | *Tetramorium grassi* | HY521 | Ant | 42 |
| Insecta |  | Hymenoptera |  | Formicidae | *Tetramorium longoi* | HY284 | Ant | 1 |
| Insecta |  | Hymenoptera |  | Formicidae | *Tetramorium pusillum* | HY520 | Ant | 16 |
| Insecta |  | Hymenoptera |  | Formicidae | *Tetramorium regulare* | HY525 | Ant | 1 |
| Insecta |  | Hymenoptera |  | Formicidae | *Tetraponera emeryi* | HY522 | Ant | 64 |
| Insecta |  | Hymenoptera |  | Formicidae | *Tetraponera natalensis* | HY523 | Ant | 1 |
| Insecta |  | Hymenoptera |  |  |  | HY1 | Predator | 1 |
| Insecta |  | Hymenoptera |  |  |  | HY10 | Predator | 1 |
| Insecta |  | Hymenoptera |  |  |  | HY100 | Predator | 12 |
| Insecta |  | Hymenoptera |  |  |  | HY101 | Predator | 2 |
| Insecta |  | Hymenoptera |  |  |  | HY102 | Predator | 144 |
| Insecta |  | Hymenoptera |  |  |  | HY103 | Predator | 3 |
| Insecta |  | Hymenoptera |  |  |  | HY104 | Predator | 1 |
| Insecta |  | Hymenoptera |  |  |  | HY105 | Predator | 1 |
| Insecta |  | Hymenoptera |  |  |  | HY106 | Predator | 5 |
| Insecta |  | Hymenoptera |  |  |  | HY107 | Predator | 2 |
| Insecta |  | Hymenoptera |  |  |  | HY108 | Predator | 1 |
| Insecta |  | Hymenoptera |  |  |  | HY109 | Predator | 1 |
| Insecta |  | Hymenoptera |  |  |  | HY11 | Predator | 3 |
| Insecta |  | Hymenoptera |  |  |  | HY110 | Predator | 3 |
| Insecta |  | Hymenoptera |  |  |  | HY111 | Predator | 1 |
| Insecta |  | Hymenoptera |  |  |  | HY112 | Predator | 29 |
| Insecta |  | Hymenoptera |  |  |  | HY113 | Predator | 13 |
| Insecta |  | Hymenoptera |  |  |  | HY114 | Predator | 1 |
| Insecta |  | Hymenoptera |  |  |  | HY115 | Predator | 1 |
| Insecta |  | Hymenoptera |  |  |  | HY116 | Predator | 4 |
| Insecta |  | Hymenoptera |  |  |  | HY117 | Predator | 5 |
| Insecta |  | Hymenoptera |  |  |  | HY118 | Predator | 158 |
| Insecta |  | Hymenoptera |  |  |  | HY119 | Predator | 3 |
| Insecta |  | Hymenoptera |  |  |  | HY12 | Predator | 1 |
| Insecta |  | Hymenoptera |  |  |  | HY120 | Predator | 1 |
| Insecta |  | Hymenoptera |  |  |  | HY121 | Predator | 2 |
| Insecta |  | Hymenoptera |  |  |  | HY126 | Predator | 1 |
| Insecta |  | Hymenoptera |  |  |  | HY127 | Predator | 2 |
| Insecta |  | Hymenoptera |  |  |  | HY128 | Predator | 13 |
| Insecta |  | Hymenoptera |  |  |  | HY129 | Predator | 4 |
| Insecta |  | Hymenoptera |  |  |  | HY13 | Predator | 123 |
| Insecta |  | Hymenoptera |  |  |  | HY130 | Predator | 5 |
| Insecta |  | Hymenoptera |  |  |  | HY131 | Predator | 1 |
| Insecta |  | Hymenoptera |  |  |  | HY132 | Predator | 4 |
| Insecta |  | Hymenoptera |  |  |  | HY133 | Predator | 1 |
| Insecta |  | Hymenoptera |  |  |  | HY134 | Predator | 13 |
| Insecta |  | Hymenoptera |  |  |  | HY135 | Predator | 7 |
| Insecta |  | Hymenoptera |  |  |  | HY136 | Predator | 7 |
| Insecta |  | Hymenoptera |  |  |  | HY137 | Predator | 10 |
| Insecta |  | Hymenoptera |  |  |  | HY138 | Predator | 1 |
| Insecta |  | Hymenoptera |  |  |  | HY139 | Predator | 7 |
| Insecta |  | Hymenoptera |  |  |  | HY14 | Predator | 16 |
| Insecta |  | Hymenoptera |  |  |  | HY140 | Predator | 9 |
| Insecta |  | Hymenoptera |  |  |  | HY141 | Predator | 2 |
| Insecta |  | Hymenoptera |  |  |  | HY142 | Predator | 1 |
| Insecta |  | Hymenoptera |  |  |  | HY143 | Predator | 1 |
| Insecta |  | Hymenoptera |  |  |  | HY144 | Predator | 2 |
| Insecta |  | Hymenoptera |  |  |  | HY145 | Predator | 18 |
| Insecta |  | Hymenoptera |  |  |  | HY146 | Predator | 3 |
| Insecta |  | Hymenoptera |  |  |  | HY147 | Predator | 1 |
| Insecta |  | Hymenoptera |  |  |  | HY148 | Predator | 3 |
| Insecta |  | Hymenoptera |  |  |  | HY149 | Predator | 2 |
| Insecta |  | Hymenoptera |  |  |  | HY15 | Predator | 3 |
| Insecta |  | Hymenoptera |  |  |  | HY150 | Predator | 2 |
| Insecta |  | Hymenoptera |  |  |  | HY151 | Predator | 1 |
| Insecta |  | Hymenoptera |  |  |  | HY152 | Predator | 18 |
| Insecta |  | Hymenoptera |  |  |  | HY153 | Predator | 21 |
| Insecta |  | Hymenoptera |  |  |  | HY154 | Predator | 4 |
| Insecta |  | Hymenoptera |  |  |  | HY155 | Predator | 27 |
| Insecta |  | Hymenoptera |  |  |  | HY156 | Predator | 13 |
| Insecta |  | Hymenoptera |  |  |  | HY157 | Predator | 9 |
| Insecta |  | Hymenoptera |  |  |  | HY158 | Predator | 12 |
| Insecta |  | Hymenoptera |  |  |  | HY159 | Predator | 2 |
| Insecta |  | Hymenoptera |  |  |  | HY16 | Predator | 6 |
| Insecta |  | Hymenoptera |  |  |  | HY160 | Predator | 4 |
| Insecta |  | Hymenoptera |  |  |  | HY161 | Predator | 3 |
| Insecta |  | Hymenoptera |  |  |  | HY162 | Predator | 3 |
| Insecta |  | Hymenoptera |  |  |  | HY163 | Predator | 37 |
| Insecta |  | Hymenoptera |  |  |  | HY164 | Predator | 6 |
| Insecta |  | Hymenoptera |  |  |  | HY165 | Predator | 31 |
| Insecta |  | Hymenoptera |  |  |  | HY166 | Predator | 6 |
| Insecta |  | Hymenoptera |  |  |  | HY167 | Predator | 5 |
| Insecta |  | Hymenoptera |  |  |  | HY168 | Predator | 1 |
| Insecta |  | Hymenoptera |  |  |  | HY169 | Predator | 2 |
| Insecta |  | Hymenoptera |  |  |  | HY170 | Predator | 2 |
| Insecta |  | Hymenoptera |  |  |  | HY171 | Predator | 1 |
| Insecta |  | Hymenoptera |  |  |  | HY173 | Predator | 1 |
| Insecta |  | Hymenoptera |  |  |  | HY174 | Predator | 4 |
| Insecta |  | Hymenoptera |  |  |  | HY175 | Predator | 1 |
| Insecta |  | Hymenoptera |  |  |  | HY176 | Predator | 3 |
| Insecta |  | Hymenoptera |  |  |  | HY177 | Predator | 3 |
| Insecta |  | Hymenoptera |  |  |  | HY178 | Predator | 4 |
| Insecta |  | Hymenoptera |  |  |  | HY179 | Predator | 3 |
| Insecta |  | Hymenoptera |  |  |  | HY18 | Predator | 4 |
| Insecta |  | Hymenoptera |  |  |  | HY180 | Predator | 10 |
| Insecta |  | Hymenoptera |  |  |  | HY181 | Predator | 28 |
| Insecta |  | Hymenoptera |  |  |  | HY182 | Predator | 3 |
| Insecta |  | Hymenoptera |  |  |  | HY183 | Predator | 4 |
| Insecta |  | Hymenoptera |  |  |  | HY184 | Predator | 13 |
| Insecta |  | Hymenoptera |  |  |  | HY185 | Predator | 2 |
| Insecta |  | Hymenoptera |  |  |  | HY186 | Predator | 3 |
| Insecta |  | Hymenoptera |  |  |  | HY187 | Predator | 4 |
| Insecta |  | Hymenoptera |  |  |  | HY189 | Predator | 33 |
| Insecta |  | Hymenoptera |  |  |  | HY190 | Predator | 9 |
| Insecta |  | Hymenoptera |  |  |  | HY191 | Predator | 4 |
| Insecta |  | Hymenoptera |  |  |  | HY192 | Predator | 6 |
| Insecta |  | Hymenoptera |  |  |  | HY193 | Predator | 1 |
| Insecta |  | Hymenoptera |  |  |  | HY194 | Predator | 1 |
| Insecta |  | Hymenoptera |  |  |  | HY195 | Predator | 13 |
| Insecta |  | Hymenoptera |  |  |  | HY196 | Predator | 1 |
| Insecta |  | Hymenoptera |  |  |  | HY197 | Predator | 5 |
| Insecta |  | Hymenoptera |  |  |  | HY198 | Predator | 2 |
| Insecta |  | Hymenoptera |  |  |  | HY199 | Predator | 21 |
| Insecta |  | Hymenoptera |  |  |  | HY2 | Predator | 18 |
| Insecta |  | Hymenoptera |  |  |  | HY200 | Predator | 5 |
| Insecta |  | Hymenoptera |  |  |  | HY201 | Predator | 3 |
| Insecta |  | Hymenoptera |  |  |  | HY202 | Predator | 2 |
| Insecta |  | Hymenoptera |  |  |  | HY203 | Predator | 2 |
| Insecta |  | Hymenoptera |  |  |  | HY204 | Predator | 4 |
| Insecta |  | Hymenoptera |  |  |  | HY205 | Predator | 2 |
| Insecta |  | Hymenoptera |  |  |  | HY206 | Predator | 4 |
| Insecta |  | Hymenoptera |  |  |  | HY207 | Predator | 6 |
| Insecta |  | Hymenoptera |  |  |  | HY208 | Predator | 1 |
| Insecta |  | Hymenoptera |  |  |  | HY209 | Predator | 3 |
| Insecta |  | Hymenoptera |  |  |  | HY210 | Predator | 5 |
| Insecta |  | Hymenoptera |  |  |  | HY211 | Predator | 1 |
| Insecta |  | Hymenoptera |  |  |  | HY212 | Predator | 3 |
| Insecta |  | Hymenoptera |  |  |  | HY213 | Predator | 1 |
| Insecta |  | Hymenoptera |  |  |  | HY214 | Predator | 1 |
| Insecta |  | Hymenoptera |  |  |  | HY215 | Predator | 35 |
| Insecta |  | Hymenoptera |  |  |  | HY216 | Predator | 4 |
| Insecta |  | Hymenoptera |  |  |  | HY217 | Predator | 2 |
| Insecta |  | Hymenoptera |  |  |  | HY218 | Predator | 8 |
| Insecta |  | Hymenoptera |  |  |  | HY219 | Predator | 3 |
| Insecta |  | Hymenoptera |  |  |  | HY220 | Predator | 2 |
| Insecta |  | Hymenoptera |  |  |  | HY222 | Predator | 7 |
| Insecta |  | Hymenoptera |  |  |  | HY223 | Predator | 11 |
| Insecta |  | Hymenoptera |  |  |  | HY224 | Predator | 3 |
| Insecta |  | Hymenoptera |  |  |  | HY225 | Predator | 1 |
| Insecta |  | Hymenoptera |  |  |  | HY226 | Predator | 1 |
| Insecta |  | Hymenoptera |  |  |  | HY227 | Predator | 1 |
| Insecta |  | Hymenoptera |  |  |  | HY228 | Predator | 2 |
| Insecta |  | Hymenoptera |  |  |  | HY229 | Predator | 1 |
| Insecta |  | Hymenoptera |  |  |  | HY230 | Predator | 1 |
| Insecta |  | Hymenoptera |  |  |  | HY231 | Predator | 1 |
| Insecta |  | Hymenoptera |  |  |  | HY233 | Predator | 2 |
| Insecta |  | Hymenoptera |  |  |  | HY234 | Predator | 4 |
| Insecta |  | Hymenoptera |  |  |  | HY235 | Predator | 1 |
| Insecta |  | Hymenoptera |  |  |  | HY236 | Predator | 1 |
| Insecta |  | Hymenoptera |  |  |  | HY237 | Predator | 3 |
| Insecta |  | Hymenoptera |  |  |  | HY238 | Predator | 1 |
| Insecta |  | Hymenoptera |  |  |  | HY239 | Predator | 4 |
| Insecta |  | Hymenoptera |  |  |  | HY24 | Predator | 8 |
| Insecta |  | Hymenoptera |  |  |  | HY240 | Predator | 3 |
| Insecta |  | Hymenoptera |  |  |  | HY241 | Predator | 1 |
| Insecta |  | Hymenoptera |  |  |  | HY242 | Predator | 1 |
| Insecta |  | Hymenoptera |  |  |  | HY243 | Predator | 2 |
| Insecta |  | Hymenoptera |  |  |  | HY244 | Predator | 1 |
| Insecta |  | Hymenoptera |  |  |  | HY245 | Predator | 6 |
| Insecta |  | Hymenoptera |  |  |  | HY246 | Predator | 1 |
| Insecta |  | Hymenoptera |  |  |  | HY247 | Predator | 12 |
| Insecta |  | Hymenoptera |  |  |  | HY248 | Predator | 1 |
| Insecta |  | Hymenoptera |  |  |  | HY249 | Predator | 6 |
| Insecta |  | Hymenoptera |  |  |  | HY250 | Predator | 1 |
| Insecta |  | Hymenoptera |  |  |  | HY251 | Predator | 6 |
| Insecta |  | Hymenoptera |  |  |  | HY252 | Predator | 2 |
| Insecta |  | Hymenoptera |  |  |  | HY253 | Predator | 2 |
| Insecta |  | Hymenoptera |  |  |  | HY254 | Predator | 11 |
| Insecta |  | Hymenoptera |  |  |  | HY255 | Predator | 1 |
| Insecta |  | Hymenoptera |  |  |  | HY256 | Predator | 23 |
| Insecta |  | Hymenoptera |  |  |  | HY257 | Predator | 1 |
| Insecta |  | Hymenoptera |  |  |  | HY258 | Predator | 6 |
| Insecta |  | Hymenoptera |  |  |  | HY259 | Predator | 4 |
| Insecta |  | Hymenoptera |  |  |  | HY26 | Predator | 1 |
| Insecta |  | Hymenoptera |  |  |  | HY260 | Predator | 2 |
| Insecta |  | Hymenoptera |  |  |  | HY261 | Predator | 1 |
| Insecta |  | Hymenoptera |  |  |  | HY262 | Predator | 2 |
| Insecta |  | Hymenoptera |  |  |  | HY263 | Predator | 4 |
| Insecta |  | Hymenoptera |  |  |  | HY264 | Predator | 1 |
| Insecta |  | Hymenoptera |  |  |  | HY265 | Predator | 6 |
| Insecta |  | Hymenoptera |  |  |  | HY266 | Predator | 1 |
| Insecta |  | Hymenoptera |  |  |  | HY267 | Predator | 1 |
| Insecta |  | Hymenoptera |  |  |  | HY268 | Predator | 1 |
| Insecta |  | Hymenoptera |  |  |  | HY269 | Predator | 10 |
| Insecta |  | Hymenoptera |  |  |  | HY27 | Predator | 1 |
| Insecta |  | Hymenoptera |  |  |  | HY270 | Predator | 2 |
| Insecta |  | Hymenoptera |  |  |  | HY271 | Predator | 2 |
| Insecta |  | Hymenoptera |  |  |  | HY272 | Predator | 7 |
| Insecta |  | Hymenoptera |  |  |  | HY273 | Predator | 5 |
| Insecta |  | Hymenoptera |  |  |  | HY274 | Predator | 3 |
| Insecta |  | Hymenoptera |  |  |  | HY275 | Predator | 2 |
| Insecta |  | Hymenoptera |  |  |  | HY276 | Predator | 3 |
| Insecta |  | Hymenoptera |  |  |  | HY277 | Predator | 3 |
| Insecta |  | Hymenoptera |  |  |  | HY278 | Predator | 3 |
| Insecta |  | Hymenoptera |  |  |  | HY279 | Predator | 1 |
| Insecta |  | Hymenoptera |  |  |  | HY28 | Predator | 1 |
| Insecta |  | Hymenoptera |  |  |  | HY280 | Predator | 1 |
| Insecta |  | Hymenoptera |  |  |  | HY281 | Predator | 5 |
| Insecta |  | Hymenoptera |  |  |  | HY282 | Predator | 2 |
| Insecta |  | Hymenoptera |  |  |  | HY283 | Predator | 2 |
| Insecta |  | Hymenoptera |  |  |  | HY285 | Predator | 3 |
| Insecta |  | Hymenoptera |  |  |  | HY286 | Predator | 4 |
| Insecta |  | Hymenoptera |  |  |  | HY287 | Predator | 1 |
| Insecta |  | Hymenoptera |  |  |  | HY288 | Predator | 2 |
| Insecta |  | Hymenoptera |  |  |  | HY289 | Predator | 11 |
| Insecta |  | Hymenoptera |  |  |  | HY29 | Predator | 40 |
| Insecta |  | Hymenoptera |  |  |  | HY290 | Predator | 5 |
| Insecta |  | Hymenoptera |  |  |  | HY291 | Predator | 2 |
| Insecta |  | Hymenoptera |  |  |  | HY292 | Predator | 1 |
| Insecta |  | Hymenoptera |  |  |  | HY293 | Predator | 3 |
| Insecta |  | Hymenoptera |  |  |  | HY294 | Predator | 2 |
| Insecta |  | Hymenoptera |  |  |  | HY295 | Predator | 1 |
| Insecta |  | Hymenoptera |  |  |  | HY296 | Predator | 39 |
| Insecta |  | Hymenoptera |  |  |  | HY297 | Predator | 1 |
| Insecta |  | Hymenoptera |  |  |  | HY298 | Predator | 7 |
| Insecta |  | Hymenoptera |  |  |  | HY299 | Predator | 7 |
| Insecta |  | Hymenoptera |  |  |  | HY30 | Predator | 8 |
| Insecta |  | Hymenoptera |  |  |  | HY302 | Predator | 3 |
| Insecta |  | Hymenoptera |  |  |  | HY303 | Predator | 1 |
| Insecta |  | Hymenoptera |  |  |  | HY304 | Predator | 1 |
| Insecta |  | Hymenoptera |  |  |  | HY305 | Predator | 1 |
| Insecta |  | Hymenoptera |  |  |  | HY306 | Predator | 1 |
| Insecta |  | Hymenoptera |  |  |  | HY307 | Predator | 1 |
| Insecta |  | Hymenoptera |  |  |  | HY308 | Predator | 3 |
| Insecta |  | Hymenoptera |  |  |  | HY309 | Predator | 1 |
| Insecta |  | Hymenoptera |  |  |  | HY310 | Predator | 1 |
| Insecta |  | Hymenoptera |  |  |  | HY311 | Predator | 1 |
| Insecta |  | Hymenoptera |  |  |  | HY312 | Predator | 1 |
| Insecta |  | Hymenoptera |  |  |  | HY313 | Predator | 1 |
| Insecta |  | Hymenoptera |  |  |  | HY314 | Predator | 2 |
| Insecta |  | Hymenoptera |  |  |  | HY315 | Predator | 1 |
| Insecta |  | Hymenoptera |  |  |  | HY316 | Predator | 1 |
| Insecta |  | Hymenoptera |  |  |  | HY317 | Predator | 1 |
| Insecta |  | Hymenoptera |  |  |  | HY318 | Predator | 6 |
| Insecta |  | Hymenoptera |  |  |  | HY319 | Predator | 1 |
| Insecta |  | Hymenoptera |  |  |  | HY32 | Predator | 2 |
| Insecta |  | Hymenoptera |  |  |  | HY320 | Predator | 1 |
| Insecta |  | Hymenoptera |  |  |  | HY321 | Predator | 1 |
| Insecta |  | Hymenoptera |  |  |  | HY322 | Predator | 1 |
| Insecta |  | Hymenoptera |  |  |  | HY323 | Predator | 1 |
| Insecta |  | Hymenoptera |  |  |  | HY324 | Predator | 3 |
| Insecta |  | Hymenoptera |  |  |  | HY325 | Predator | 1 |
| Insecta |  | Hymenoptera |  |  |  | HY326 | Predator | 3 |
| Insecta |  | Hymenoptera |  |  |  | HY327 | Predator | 1 |
| Insecta |  | Hymenoptera |  |  |  | HY328 | Predator | 1 |
| Insecta |  | Hymenoptera |  |  |  | HY329 | Predator | 1 |
| Insecta |  | Hymenoptera |  |  |  | HY33 | Predator | 41 |
| Insecta |  | Hymenoptera |  |  |  | HY330 | Predator | 2 |
| Insecta |  | Hymenoptera |  |  |  | HY331 | Predator | 3 |
| Insecta |  | Hymenoptera |  |  |  | HY332 | Predator | 1 |
| Insecta |  | Hymenoptera |  |  |  | HY333 | Predator | 8 |
| Insecta |  | Hymenoptera |  |  |  | HY334 | Predator | 4 |
| Insecta |  | Hymenoptera |  |  |  | HY335 | Predator | 2 |
| Insecta |  | Hymenoptera |  |  |  | HY336 | Predator | 1 |
| Insecta |  | Hymenoptera |  |  |  | HY337 | Predator | 2 |
| Insecta |  | Hymenoptera |  |  |  | HY338 | Predator | 1 |
| Insecta |  | Hymenoptera |  |  |  | HY339 | Predator | 2 |
| Insecta |  | Hymenoptera |  |  |  | HY34 | Predator | 6 |
| Insecta |  | Hymenoptera |  |  |  | HY340 | Predator | 2 |
| Insecta |  | Hymenoptera |  |  |  | HY341 | Predator | 1 |
| Insecta |  | Hymenoptera |  |  |  | HY342 | Predator | 1 |
| Insecta |  | Hymenoptera |  |  |  | HY343 | Predator | 3 |
| Insecta |  | Hymenoptera |  |  |  | HY344 | Predator | 1 |
| Insecta |  | Hymenoptera |  |  |  | HY345 | Predator | 1 |
| Insecta |  | Hymenoptera |  |  |  | HY346 | Predator | 2 |
| Insecta |  | Hymenoptera |  |  |  | HY348 | Predator | 71 |
| Insecta |  | Hymenoptera |  |  |  | HY349 | Predator | 3 |
| Insecta |  | Hymenoptera |  |  |  | HY35 | Predator | 4 |
| Insecta |  | Hymenoptera |  |  |  | HY350 | Predator | 3 |
| Insecta |  | Hymenoptera |  |  |  | HY351 | Predator | 1 |
| Insecta |  | Hymenoptera |  |  |  | HY352 | Predator | 3 |
| Insecta |  | Hymenoptera |  |  |  | HY353 | Predator | 2 |
| Insecta |  | Hymenoptera |  |  |  | HY354 | Predator | 2 |
| Insecta |  | Hymenoptera |  |  |  | HY356 | Predator | 1 |
| Insecta |  | Hymenoptera |  |  |  | HY357 | Predator | 1 |
| Insecta |  | Hymenoptera |  |  |  | HY358 | Predator | 1 |
| Insecta |  | Hymenoptera |  |  |  | HY359 | Predator | 2 |
| Insecta |  | Hymenoptera |  |  |  | HY36 | Predator | 8 |
| Insecta |  | Hymenoptera |  |  |  | HY360 | Predator | 4 |
| Insecta |  | Hymenoptera |  |  |  | HY361 | Predator | 1 |
| Insecta |  | Hymenoptera |  |  |  | HY362 | Predator | 5 |
| Insecta |  | Hymenoptera |  |  |  | HY363 | Predator | 2 |
| Insecta |  | Hymenoptera |  |  |  | HY364 | Predator | 4 |
| Insecta |  | Hymenoptera |  |  |  | HY365 | Predator | 2 |
| Insecta |  | Hymenoptera |  |  |  | HY366 | Predator | 1 |
| Insecta |  | Hymenoptera |  |  |  | HY367 | Predator | 2 |
| Insecta |  | Hymenoptera |  |  |  | HY368 | Predator | 1 |
| Insecta |  | Hymenoptera |  |  |  | HY369 | Predator | 1 |
| Insecta |  | Hymenoptera |  |  |  | HY37 | Predator | 129 |
| Insecta |  | Hymenoptera |  |  |  | HY370 | Predator | 1 |
| Insecta |  | Hymenoptera |  |  |  | HY371 | Predator | 1 |
| Insecta |  | Hymenoptera |  |  |  | HY372 | Predator | 2 |
| Insecta |  | Hymenoptera |  |  |  | HY373 | Predator | 2 |
| Insecta |  | Hymenoptera |  |  |  | HY374 | Predator | 1 |
| Insecta |  | Hymenoptera |  |  |  | HY375 | Predator | 1 |
| Insecta |  | Hymenoptera |  |  |  | HY376 | Predator | 1 |
| Insecta |  | Hymenoptera |  |  |  | HY377 | Predator | 2 |
| Insecta |  | Hymenoptera |  |  |  | HY378 | Predator | 1 |
| Insecta |  | Hymenoptera |  |  |  | HY379 | Predator | 1 |
| Insecta |  | Hymenoptera |  |  |  | HY38 | Predator | 5 |
| Insecta |  | Hymenoptera |  |  |  | HY380 | Predator | 1 |
| Insecta |  | Hymenoptera |  |  |  | HY381 | Predator | 1 |
| Insecta |  | Hymenoptera |  |  |  | HY382 | Predator | 1 |
| Insecta |  | Hymenoptera |  |  |  | HY383 | Predator | 3 |
| Insecta |  | Hymenoptera |  |  |  | HY384 | Predator | 6 |
| Insecta |  | Hymenoptera |  |  |  | HY385 | Predator | 3 |
| Insecta |  | Hymenoptera |  |  |  | HY386 | Predator | 2 |
| Insecta |  | Hymenoptera |  |  |  | HY387 | Predator | 1 |
| Insecta |  | Hymenoptera |  |  |  | HY388 | Predator | 1 |
| Insecta |  | Hymenoptera |  |  |  | HY389 | Predator | 1 |
| Insecta |  | Hymenoptera |  |  |  | HY39 | Predator | 6 |
| Insecta |  | Hymenoptera |  |  |  | HY390 | Predator | 3 |
| Insecta |  | Hymenoptera |  |  |  | HY391 | Predator | 1 |
| Insecta |  | Hymenoptera |  |  |  | HY392 | Predator | 1 |
| Insecta |  | Hymenoptera |  |  |  | HY393 | Predator | 4 |
| Insecta |  | Hymenoptera |  |  |  | HY394 | Predator | 3 |
| Insecta |  | Hymenoptera |  |  |  | HY395 | Predator | 1 |
| Insecta |  | Hymenoptera |  |  |  | HY396 | Predator | 1 |
| Insecta |  | Hymenoptera |  |  |  | HY397 | Predator | 3 |
| Insecta |  | Hymenoptera |  |  |  | HY398 | Predator | 1 |
| Insecta |  | Hymenoptera |  |  |  | HY399 | Predator | 1 |
| Insecta |  | Hymenoptera |  |  |  | HY4 | Predator | 53 |
| Insecta |  | Hymenoptera |  |  |  | HY40 | Predator | 1 |
| Insecta |  | Hymenoptera |  |  |  | HY400 | Predator | 1 |
| Insecta |  | Hymenoptera |  |  |  | HY401 | Predator | 1 |
| Insecta |  | Hymenoptera |  |  |  | HY402 | Predator | 3 |
| Insecta |  | Hymenoptera |  |  |  | HY403 | Predator | 2 |
| Insecta |  | Hymenoptera |  |  |  | HY404 | Predator | 1 |
| Insecta |  | Hymenoptera |  |  |  | HY405 | Predator | 2 |
| Insecta |  | Hymenoptera |  |  |  | HY406 | Predator | 1 |
| Insecta |  | Hymenoptera |  |  |  | HY407 | Predator | 1 |
| Insecta |  | Hymenoptera |  |  |  | HY408 | Predator | 1 |
| Insecta |  | Hymenoptera |  |  |  | HY409 | Predator | 3 |
| Insecta |  | Hymenoptera |  |  |  | HY41 | Predator | 44 |
| Insecta |  | Hymenoptera |  |  |  | HY410 | Predator | 4 |
| Insecta |  | Hymenoptera |  |  |  | HY411 | Predator | 2 |
| Insecta |  | Hymenoptera |  |  |  | HY412 | Predator | 4 |
| Insecta |  | Hymenoptera |  |  |  | HY413 | Predator | 1 |
| Insecta |  | Hymenoptera |  |  |  | HY416 | Predator | 1 |
| Insecta |  | Hymenoptera |  |  |  | HY417 | Predator | 1 |
| Insecta |  | Hymenoptera |  |  |  | HY418 | Predator | 1 |
| Insecta |  | Hymenoptera |  |  |  | HY419 | Predator | 1 |
| Insecta |  | Hymenoptera |  |  |  | HY42 | Predator | 2 |
| Insecta |  | Hymenoptera |  |  |  | HY420 | Predator | 2 |
| Insecta |  | Hymenoptera |  |  |  | HY421 | Predator | 1 |
| Insecta |  | Hymenoptera |  |  |  | HY422 | Predator | 1 |
| Insecta |  | Hymenoptera |  |  |  | HY423 | Predator | 2 |
| Insecta |  | Hymenoptera |  |  |  | HY424 | Predator | 1 |
| Insecta |  | Hymenoptera |  |  |  | HY425 | Predator | 1 |
| Insecta |  | Hymenoptera |  |  |  | HY426 | Predator | 3 |
| Insecta |  | Hymenoptera |  |  |  | HY427 | Predator | 1 |
| Insecta |  | Hymenoptera |  |  |  | HY428 | Predator | 1 |
| Insecta |  | Hymenoptera |  |  |  | HY429 | Predator | 1 |
| Insecta |  | Hymenoptera |  |  |  | HY43 | Predator | 8 |
| Insecta |  | Hymenoptera |  |  |  | HY430 | Predator | 2 |
| Insecta |  | Hymenoptera |  |  |  | HY431 | Predator | 3 |
| Insecta |  | Hymenoptera |  |  |  | HY432 | Predator | 8 |
| Insecta |  | Hymenoptera |  |  |  | HY433 | Predator | 1 |
| Insecta |  | Hymenoptera |  |  |  | HY434 | Predator | 1 |
| Insecta |  | Hymenoptera |  |  |  | HY435 | Predator | 2 |
| Insecta |  | Hymenoptera |  |  |  | HY436 | Predator | 1 |
| Insecta |  | Hymenoptera |  |  |  | HY437 | Predator | 5 |
| Insecta |  | Hymenoptera |  |  |  | HY438 | Predator | 1 |
| Insecta |  | Hymenoptera |  |  |  | HY439 | Predator | 1 |
| Insecta |  | Hymenoptera |  |  |  | HY440 | Predator | 1 |
| Insecta |  | Hymenoptera |  |  |  | HY441 | Predator | 1 |
| Insecta |  | Hymenoptera |  |  |  | HY442 | Predator | 1 |
| Insecta |  | Hymenoptera |  |  |  | HY443 | Predator | 2 |
| Insecta |  | Hymenoptera |  |  |  | HY444 | Predator | 1 |
| Insecta |  | Hymenoptera |  |  |  | HY445 | Predator | 1 |
| Insecta |  | Hymenoptera |  |  |  | HY446 | Predator | 1 |
| Insecta |  | Hymenoptera |  |  |  | HY447 | Predator | 1 |
| Insecta |  | Hymenoptera |  |  |  | HY448 | Predator | 1 |
| Insecta |  | Hymenoptera |  |  |  | HY45 | Predator | 22 |
| Insecta |  | Hymenoptera |  |  |  | HY450 | Predator | 1 |
| Insecta |  | Hymenoptera |  |  |  | HY451 | Predator | 1 |
| Insecta |  | Hymenoptera |  |  |  | HY453 | Predator | 1 |
| Insecta |  | Hymenoptera |  |  |  | HY454 | Predator | 1 |
| Insecta |  | Hymenoptera |  |  |  | HY456 | Predator | 1 |
| Insecta |  | Hymenoptera |  |  |  | HY457 | Predator | 1 |
| Insecta |  | Hymenoptera |  |  |  | HY458 | Predator | 2 |
| Insecta |  | Hymenoptera |  |  |  | HY459 | Predator | 3 |
| Insecta |  | Hymenoptera |  |  |  | HY46 | Predator | 13 |
| Insecta |  | Hymenoptera |  |  |  | HY460 | Predator | 1 |
| Insecta |  | Hymenoptera |  |  |  | HY461 | Predator | 1 |
| Insecta |  | Hymenoptera |  |  |  | HY462 | Predator | 2 |
| Insecta |  | Hymenoptera |  |  |  | HY463 | Predator | 1 |
| Insecta |  | Hymenoptera |  |  |  | HY464 | Predator | 1 |
| Insecta |  | Hymenoptera |  |  |  | HY465 | Predator | 1 |
| Insecta |  | Hymenoptera |  |  |  | HY466 | Predator | 1 |
| Insecta |  | Hymenoptera |  |  |  | HY467 | Predator | 1 |
| Insecta |  | Hymenoptera |  |  |  | HY468 | Predator | 1 |
| Insecta |  | Hymenoptera |  |  |  | HY469 | Predator | 2 |
| Insecta |  | Hymenoptera |  |  |  | HY47 | Predator | 2 |
| Insecta |  | Hymenoptera |  |  |  | HY470 | Predator | 1 |
| Insecta |  | Hymenoptera |  |  |  | HY471 | Predator | 1 |
| Insecta |  | Hymenoptera |  |  |  | HY474 | Predator | 2 |
| Insecta |  | Hymenoptera |  |  |  | HY475 | Predator | 1 |
| Insecta |  | Hymenoptera |  |  |  | HY476 | Predator | 1 |
| Insecta |  | Hymenoptera |  |  |  | HY477 | Predator | 1 |
| Insecta |  | Hymenoptera |  |  |  | HY478 | Predator | 3 |
| Insecta |  | Hymenoptera |  |  |  | HY479 | Predator | 2 |
| Insecta |  | Hymenoptera |  |  |  | HY48 | Predator | 29 |
| Insecta |  | Hymenoptera |  |  |  | HY480 | Predator | 1 |
| Insecta |  | Hymenoptera |  |  |  | HY481 | Predator | 4 |
| Insecta |  | Hymenoptera |  |  |  | HY482 | Predator | 1 |
| Insecta |  | Hymenoptera |  |  |  | HY483 | Predator | 1 |
| Insecta |  | Hymenoptera |  |  |  | HY484 | Predator | 3 |
| Insecta |  | Hymenoptera |  |  |  | HY485 | Predator | 1 |
| Insecta |  | Hymenoptera |  |  |  | HY486 | Predator | 1 |
| Insecta |  | Hymenoptera |  |  |  | HY487 | Predator | 1 |
| Insecta |  | Hymenoptera |  |  |  | HY488 | Predator | 3 |
| Insecta |  | Hymenoptera |  |  |  | HY489 | Predator | 1 |
| Insecta |  | Hymenoptera |  |  |  | HY49 | Predator | 11 |
| Insecta |  | Hymenoptera |  |  |  | HY490 | Predator | 4 |
| Insecta |  | Hymenoptera |  |  |  | HY491 | Predator | 1 |
| Insecta |  | Hymenoptera |  |  |  | HY492 | Predator | 1 |
| Insecta |  | Hymenoptera |  |  |  | HY493 | Predator | 1 |
| Insecta |  | Hymenoptera |  |  |  | HY494 | Predator | 1 |
| Insecta |  | Hymenoptera |  |  |  | HY495 | Predator | 1 |
| Insecta |  | Hymenoptera |  |  |  | HY496 | Predator | 1 |
| Insecta |  | Hymenoptera |  |  |  | HY497 | Predator | 1 |
| Insecta |  | Hymenoptera |  |  |  | HY498 | Predator | 1 |
| Insecta |  | Hymenoptera |  |  |  | HY499 | Predator | 1 |
| Insecta |  | Hymenoptera |  |  |  | HY5 | Predator | 5 |
| Insecta |  | Hymenoptera |  |  |  | HY50 | Predator | 2 |
| Insecta |  | Hymenoptera |  |  |  | HY500 | Predator | 1 |
| Insecta |  | Hymenoptera |  |  |  | HY501 | Predator | 1 |
| Insecta |  | Hymenoptera |  |  |  | HY502 | Predator | 1 |
| Insecta |  | Hymenoptera |  |  |  | HY504 | Predator | 1 |
| Insecta |  | Hymenoptera |  |  |  | HY505 | Predator | 1 |
| Insecta |  | Hymenoptera |  |  |  | HY506 | Predator | 1 |
| Insecta |  | Hymenoptera |  |  |  | HY507 | Predator | 1 |
| Insecta |  | Hymenoptera |  |  |  | HY508 | Predator | 1 |
| Insecta |  | Hymenoptera |  |  |  | HY509 | Predator | 1 |
| Insecta |  | Hymenoptera |  |  |  | HY51 | Predator | 31 |
| Insecta |  | Hymenoptera |  |  |  | HY510 | Predator | 1 |
| Insecta |  | Hymenoptera |  |  |  | HY511 | Predator | 1 |
| Insecta |  | Hymenoptera |  |  |  | HY512 | Predator | 1 |
| Insecta |  | Hymenoptera |  |  |  | HY52 | Predator | 27 |
| Insecta |  | Hymenoptera |  |  |  | HY53 | Predator | 11 |
| Insecta |  | Hymenoptera |  |  |  | HY54 | Predator | 17 |
| Insecta |  | Hymenoptera |  |  |  | HY55 | Predator | 1 |
| Insecta |  | Hymenoptera |  |  |  | HY56 | Predator | 1 |
| Insecta |  | Hymenoptera |  |  |  | HY57 | Predator | 4 |
| Insecta |  | Hymenoptera |  |  |  | HY58 | Predator | 24 |
| Insecta |  | Hymenoptera |  |  |  | HY6 | Predator | 2 |
| Insecta |  | Hymenoptera |  |  |  | HY62 | Predator | 1 |
| Insecta |  | Hymenoptera |  |  |  | HY63 | Predator | 34 |
| Insecta |  | Hymenoptera |  |  |  | HY64 | Predator | 6 |
| Insecta |  | Hymenoptera |  |  |  | HY65 | Predator | 1 |
| Insecta |  | Hymenoptera |  |  |  | HY67 | Predator | 2 |
| Insecta |  | Hymenoptera |  |  |  | HY68 | Predator | 1 |
| Insecta |  | Hymenoptera |  |  |  | HY7 | Predator | 4 |
| Insecta |  | Hymenoptera |  |  |  | HY70 | Predator | 5 |
| Insecta |  | Hymenoptera |  |  |  | HY71 | Predator | 51 |
| Insecta |  | Hymenoptera |  |  |  | HY72 | Predator | 9 |
| Insecta |  | Hymenoptera |  |  |  | HY73 | Predator | 2 |
| Insecta |  | Hymenoptera |  |  |  | HY74 | Predator | 36 |
| Insecta |  | Hymenoptera |  |  |  | HY75 | Predator | 27 |
| Insecta |  | Hymenoptera |  |  |  | HY76 | Predator | 14 |
| Insecta |  | Hymenoptera |  |  |  | HY77 | Predator | 1 |
| Insecta |  | Hymenoptera |  |  |  | HY78 | Predator | 30 |
| Insecta |  | Hymenoptera |  |  |  | HY79 | Predator | 3 |
| Insecta |  | Hymenoptera |  |  |  | HY8 | Predator | 2 |
| Insecta |  | Hymenoptera |  |  |  | HY80 | Predator | 2 |
| Insecta |  | Hymenoptera |  |  |  | HY81 | Predator | 1 |
| Insecta |  | Hymenoptera |  |  |  | HY82 | Predator | 2 |
| Insecta |  | Hymenoptera |  |  |  | HY83 | Predator | 22 |
| Insecta |  | Hymenoptera |  |  |  | HY85 | Predator | 1 |
| Insecta |  | Hymenoptera |  |  |  | HY86 | Predator | 90 |
| Insecta |  | Hymenoptera |  |  |  | HY87 | Predator | 2 |
| Insecta |  | Hymenoptera |  |  |  | HY88 | Predator | 4 |
| Insecta |  | Hymenoptera |  |  |  | HY89 | Predator | 5 |
| Insecta |  | Hymenoptera |  |  |  | HY9 | Predator | 29 |
| Insecta |  | Hymenoptera |  |  |  | HY93 | Predator | 9 |
| Insecta |  | Hymenoptera |  |  |  | HY94 | Predator | 1 |
| Insecta |  | Hymenoptera |  |  |  | HY95 | Predator | 6 |
| Insecta |  | Hymenoptera |  |  |  | HY96 | Predator | 1 |
| Insecta |  | Hymenoptera |  |  |  | HY97 | Predator | 5 |
| Insecta |  | Hymenoptera |  |  |  | HY98 | Predator | 4 |
| Insecta |  | Hymenoptera |  |  |  | HY99 | Predator | 1 |
| Insecta |  | Hymenoptera |  |  |  | LA39 | Predator | 5 |
| Insecta |  | Hymenoptera |  |  |  | LA41 | Predator | 1 |
| Insecta |  | Hymenoptera |  |  |  | LA47 | Predator | 1 |
| Insecta |  | Hymenoptera |  |  |  | LA73 | Predator | 1 |
| Insecta |  | Lepidoptera |  | Adelidae |  | L22 | Pollinator | 2 |
| Insecta |  | Lepidoptera |  | Adelidae |  | L25 | Pollinator | 1 |
| Insecta |  | Lepidoptera |  | Arctiidae |  | L1 | Pollinator | 22 |
| Insecta |  | Lepidoptera |  | Arctiidae |  | L18 | Pollinator | 2 |
| Insecta |  | Lepidoptera |  | Arctiidae |  | L21 | Pollinator | 2 |
| Insecta |  | Lepidoptera |  | Arctiidae |  | L26 | Pollinator | 1 |
| Insecta |  | Lepidoptera |  | Arctiidae |  | L28 | Pollinator | 1 |
| Insecta |  | Lepidoptera |  | Arctiidae |  | L4 | Pollinator | 9 |
| Insecta |  | Lepidoptera |  | Arctiidae |  | L6 | Pollinator | 1 |
| Insecta |  | Lepidoptera |  | Arctiidae |  | L8 | Pollinator | 10 |
| Insecta |  | Lepidoptera |  | Noctuidae |  | L10 | Pollinator | 1 |
| Insecta |  | Lepidoptera |  | Noctuidae |  | L12 | Pollinator | 1 |
| Insecta |  | Lepidoptera |  | Noctuidae |  | L13 | Pollinator | 1 |
| Insecta |  | Lepidoptera |  | Noctuidae |  | L14 | Pollinator | 1 |
| Insecta |  | Lepidoptera |  | Noctuidae |  | L16 | Pollinator | 1 |
| Insecta |  | Lepidoptera |  | Noctuidae |  | L23 | Pollinator | 1 |
| Insecta |  | Lepidoptera |  | Noctuidae |  | L24 | Pollinator | 1 |
| Insecta |  | Lepidoptera |  | Noctuidae |  | L7 | Pollinator | 9 |
| Insecta |  | Lepidoptera |  | Noctuidae |  | L9 | Pollinator | 2 |
| Insecta |  | Lepidoptera |  | Pyralidae |  | L15 | Pollinator | 1 |
| Insecta |  | Lepidoptera |  | Pyralidae |  | L17 | Pollinator | 1 |
| Insecta |  | Lepidoptera |  | Pyralidae |  | L2 | Pollinator | 1 |
| Insecta |  | Lepidoptera |  | Pyralidae |  | L20 | Pollinator | 1 |
| Insecta |  | Lepidoptera |  | Pyralidae |  | L5 | Pollinator | 2 |
| Insecta |  | Lepidoptera |  |  |  | L3 | Pollinator | 4 |
| Insecta |  | Lepidoptera |  | Arctiidae |  | LA52 | Herbivore | 1 |
| Insecta |  | Lepidoptera |  | Arctiidae |  | LA57 | Herbivore | 1 |
| Insecta |  | Lepidoptera |  | Eupterotidae |  | LA21 | Herbivore | 1 |
| Insecta |  | Lepidoptera |  | Geometridae |  | LA18 | Herbivore | 1 |
| Insecta |  | Lepidoptera |  | Geometridae |  | LA33 | Herbivore | 1 |
| Insecta |  | Lepidoptera |  | Geometridae |  | LA43 | Herbivore | 1 |
| Insecta |  | Lepidoptera |  | Geometridae |  | LA46 | Herbivore | 1 |
| Insecta |  | Lepidoptera |  | Geometridae |  | LA56 | Herbivore | 3 |
| Insecta |  | Lepidoptera |  | Lasiocampidae |  | LA49 | Herbivore | 1 |
| Insecta |  | Lepidoptera |  | Lasiocampidae |  | LA5 | Herbivore | 2 |
| Insecta |  | Lepidoptera |  | Limacodidae |  | LA40 | Herbivore | 1 |
| Insecta |  | Lepidoptera |  | Limacodidae |  | LA6 | Herbivore | 2 |
| Insecta |  | Lepidoptera |  | Lycaenidae |  | LA44 | Herbivore | 1 |
| Insecta |  | Lepidoptera |  | Noctuidae |  | LA14 | Herbivore | 1 |
| Insecta |  | Lepidoptera |  | Noctuidae |  | LA16 | Herbivore | 2 |
| Insecta |  | Lepidoptera |  | Noctuidae |  | LA2 | Herbivore | 5 |
| Insecta |  | Lepidoptera |  | Noctuidae |  | LA34 | Herbivore | 12 |
| Insecta |  | Lepidoptera |  | Noctuidae |  | LA35 | Herbivore | 1 |
| Insecta |  | Lepidoptera |  | Noctuidae |  | LA45 | Herbivore | 1 |
| Insecta |  | Lepidoptera |  | Noctuidae |  | LA50 | Herbivore | 2 |
| Insecta |  | Lepidoptera |  | Noctuidae |  | LA62 | Herbivore | 4 |
| Insecta |  | Lepidoptera |  | Noctuidae |  | LA66 | Herbivore | 1 |
| Insecta |  | Lepidoptera |  | Noctuidae |  | LA72 | Herbivore | 1 |
| Insecta |  | Lepidoptera |  | Noctuidae |  | LA74 | Herbivore | 1 |
| Insecta |  | Lepidoptera |  | Noctuidae |  | LA8 | Herbivore | 12 |
| Insecta |  | Lepidoptera |  | Noctuidae |  | LA9 | Herbivore | 1 |
| Insecta |  | Lepidoptera |  | Sphingidae |  | LA69 | Herbivore | 1 |
| Insecta |  | Lepidoptera |  | Zygaeinidae |  | LA19 | Herbivore | 11 |
| Insecta |  | Lepidoptera |  |  |  | LA1 | Herbivore | 17 |
| Insecta |  | Lepidoptera |  |  |  | LA42 | Herbivore | 3 |
| Insecta |  | Mantodea |  | Mantidae |  | M1 | Predator | 1 |
| Insecta |  | Neuroptera |  | Chrysopidae |  | C18 | Predator | 44 |
| Insecta |  | Neuroptera |  | Chrysopidae |  | N1 | Predator | 33 |
| Insecta |  | Neuroptera |  | Chrysopidae |  | N11 | Predator | 4 |
| Insecta |  | Neuroptera |  | Chrysopidae |  | N2 | Predator | 4 |
| Insecta |  | Neuroptera |  | Chrysopidae |  | N4 | Predator | 1 |
| Insecta |  | Neuroptera |  | Chrysopidae |  | N5 | Predator | 6 |
| Insecta |  | Neuroptera |  | Chrysopidae |  | N7 | Predator | 1 |
| Insecta |  | Neuroptera |  | Chrysopidae |  | N8 | Predator | 1 |
| Insecta |  | Neuroptera |  | Chrysopidae |  | N9 | Predator | 12 |
| Insecta |  | Neuroptera |  | Hemerobiidae |  | K13 | Predator | 3 |
| Insecta |  | Neuroptera |  | Hemerobiidae |  | L11 | Predator | 1 |
| Insecta |  | Neuroptera |  | Hemerobiidae |  | N3 | Predator | 22 |
| Insecta |  | Neuroptera |  | Hemerobiidae |  | N6 | Predator | 4 |
| Insecta |  | Orthoptera |  | Acrididae |  | O11 | Herbivore | 5 |
| Insecta |  | Orthoptera |  | Acrididae |  | O13 | Herbivore | 1 |
| Insecta |  | Orthoptera |  | Acrididae |  | O17 | Herbivore | 2 |
| Insecta |  | Orthoptera |  | Acrididae |  | O19 | Herbivore | 1 |
| Insecta |  | Orthoptera |  | Acrididae |  | O20 | Herbivore | 1 |
| Insecta |  | Orthoptera |  | Acrididae |  | O6 | Herbivore | 1 |
| Insecta |  | Orthoptera |  | Acrididae |  | O8 | Herbivore | 1 |
| Insecta |  | Orthoptera |  | Gryllidae |  | O12 | Herbivore | 1 |
| Insecta |  | Orthoptera |  | Gryllidae |  | O16 | Herbivore | 1 |
| Insecta |  | Orthoptera |  | Gryllidae |  | O18 | Herbivore | 1 |
| Insecta |  | Orthoptera |  | Gryllidae |  | O3 | Herbivore | 48 |
| Insecta |  | Orthoptera |  | Gryllidae |  | O5 | Herbivore | 1 |
| Insecta |  | Orthoptera |  | Gryllidae |  | O9 | Herbivore | 11 |
| Insecta |  | Orthoptera |  | Pamphagidae |  | O4 | Herbivore | 1 |
| Insecta |  | Orthoptera |  | Tettigoniidae |  | O1 | Herbivore | 1 |
| Insecta |  | Orthoptera |  | Tettigoniidae |  | O10 | Herbivore | 1 |
| Insecta |  | Orthoptera |  | Tettigoniidae |  | O14 | Herbivore | 1 |
| Insecta |  | Orthoptera |  | Tettigoniidae |  | O15 | Herbivore | 3 |
| Insecta |  | Orthoptera |  | Tettigoniidae |  | O2 | Herbivore | 2 |
| Insecta |  | Orthoptera |  | Tettigoniidae |  | O7 | Herbivore | 1 |
| Insecta |  | Phasmatodea |  | Heteronemiidae |  | PH2 | Herbivore | 3 |
| Insecta |  | Phasmatodea |  | Heteronemiidae |  | PH3 | Herbivore | 1 |
| Insecta |  | Phasmatodea |  | Phasmatidae |  | PH1 | Herbivore | 3 |
| Insecta |  | Plecoptera |  | Nemouridae |  | K2 | Detritivore | 5 |
| Insecta |  | Plecoptera |  | Nemouridae |  | N10 | Detritivore | 1 |
| Insecta |  | Psocoptera |  | Ectopsocidae |  | K1 | Detritivore | 24 |
| Insecta |  | Psocoptera |  | Ectopsocidae |  | K14 | Detritivore | 1 |
| Insecta |  | Psocoptera |  | Ectopsocidae |  | K4 | Detritivore | 169 |
| Insecta |  | Psocoptera |  | Hemipsocidae |  | K10 | Detritivore | 3 |
| Insecta |  | Psocoptera |  | Hemipsocidae |  | K15 | Detritivore | 4 |
| Insecta |  | Psocoptera |  | Hemipsocidae |  | K5 | Detritivore | 5 |
| Insecta |  | Psocoptera |  | Lachesillidae |  | K3 | Detritivore | 75 |
| Insecta |  | Psocoptera |  | Psocidae |  | K16 | Detritivore | 1 |
| Insecta |  | Psocoptera |  | Psocidae |  | K17 | Detritivore | 1 |
| Insecta |  | Psocoptera |  | Psocidae |  | K18 | Detritivore | 11 |
| Insecta |  | Psocoptera |  | Psocidae |  | K6 | Detritivore | 15 |
| Insecta |  | Psocoptera |  | Psocidae |  | K8 | Detritivore | 33 |
| Insecta |  | Psocoptera |  | Psocidae |  | K9 | Detritivore | 11 |
| Insecta |  | Psocoptera |  |  |  | K12 | Detritivore | 1 |
| Insecta |  | Psocoptera |  |  |  | K19 | Detritivore | 1 |
| Insecta |  | Thysanoptera |  | Merothripidae |  | TY10 | Detritivore | 7 |
| Insecta |  | Thysanoptera |  | Merothripidae |  | TY6 | Detritivore | 2 |
| Insecta |  | Thysanoptera |  | Merothripidae |  | TY9 | Detritivore | 77 |
| Insecta |  | Thysanoptera |  | Thripidae |  | E1 | Detritivore | 2 |
| Insecta |  | Thysanoptera |  | Thripidae |  | LA27 | Detritivore | 5 |
| Insecta |  | Thysanoptera |  | Thripidae |  | TY1 | Detritivore | 64 |
| Insecta |  | Thysanoptera |  | Thripidae |  | TY11 | Detritivore | 60 |
| Insecta |  | Thysanoptera |  | Thripidae |  | TY12 | Detritivore | 18 |
| Insecta |  | Thysanoptera |  | Thripidae |  | TY13 | Detritivore | 1 |
| Insecta |  | Thysanoptera |  | Thripidae |  | TY14 | Detritivore | 14 |
| Insecta |  | Thysanoptera |  | Thripidae |  | TY15 | Detritivore | 39 |
| Insecta |  | Thysanoptera |  | Thripidae |  | TY16 | Detritivore | 10 |
| Insecta |  | Thysanoptera |  | Thripidae |  | TY17 | Detritivore | 23 |
| Insecta |  | Thysanoptera |  | Thripidae |  | TY18 | Detritivore | 4 |
| Insecta |  | Thysanoptera |  | Thripidae |  | TY19 | Detritivore | 2 |
| Insecta |  | Thysanoptera |  | Thripidae |  | TY2 | Detritivore | 103 |
| Insecta |  | Thysanoptera |  | Thripidae |  | TY20 | Detritivore | 1 |
| Insecta |  | Thysanoptera |  | Thripidae |  | TY21 | Detritivore | 12 |
| Insecta |  | Thysanoptera |  | Thripidae |  | TY3 | Detritivore | 5 |
| Insecta |  | Thysanoptera |  | Thripidae |  | TY4 | Detritivore | 50 |
| Insecta |  | Thysanoptera |  | Thripidae |  | TY5 | Detritivore | 18 |
| Insecta |  | Thysanoptera |  | Thripidae |  | TY7 | Detritivore | 42 |
| Insecta |  | Thysanoptera |  | Thripidae |  | TY8 | Detritivore | 9 |
| Malacostraca |  | Amphipoda |  |  |  | I1 | Detritivore | 51 |
| Malacostraca |  | Isopoda |  |  |  | I11 | Detritivore | 1 |
| Malacostraca |  | Isopoda |  |  |  | I2 | Detritivore | 1 |
| Malacostraca |  | Isopoda |  |  |  | I3 | Detritivore | 4 |
| Malacostraca |  | Isopoda |  |  |  | I4 | Detritivore | 3 |
| Malacostraca |  | Isopoda |  |  |  | I5 | Detritivore | 80 |
| Malacostraca |  | Isopoda |  |  |  | I7 | Detritivore | 3 |
| Malacostraca |  | Isopoda |  |  |  | I8 | Detritivore | 6 |


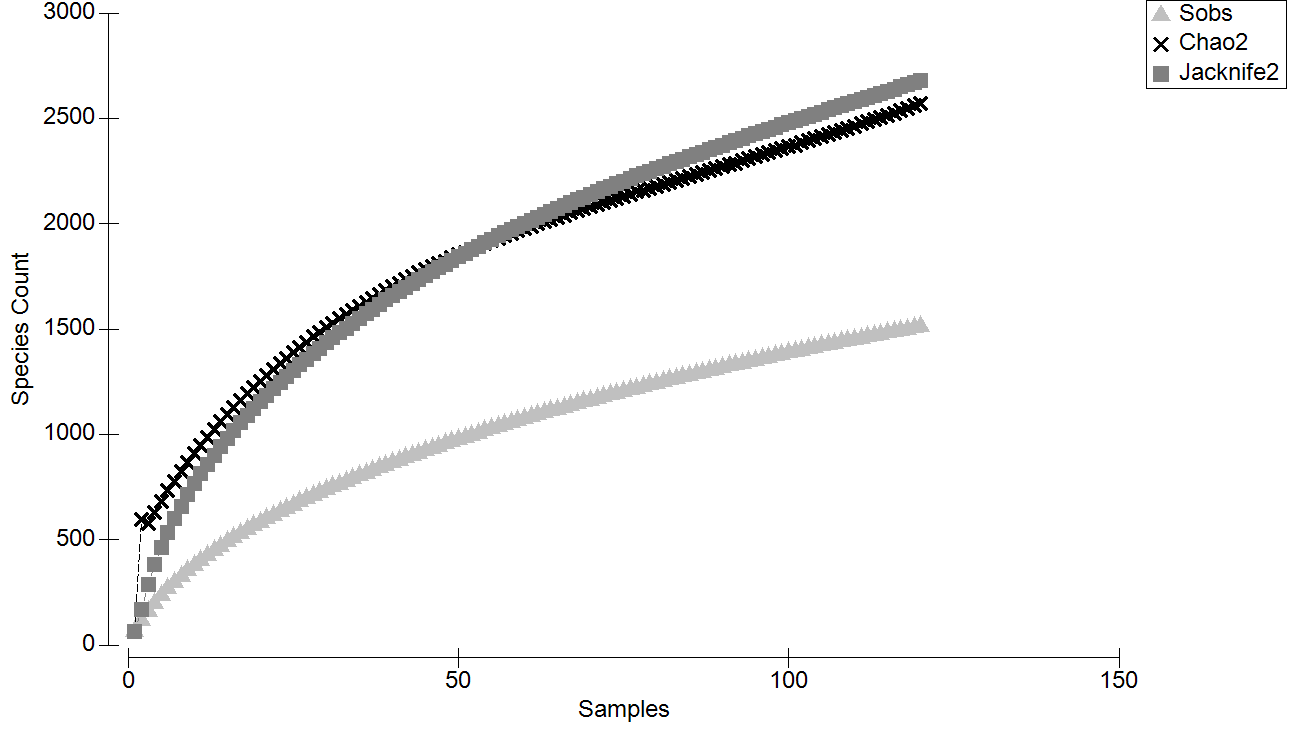


**Figure S1-1:** Species accumulation curves for overall arthropod richness sampled from southern Afro-temperate forest canopies featuring both Chao2 and Jacknife2 indices


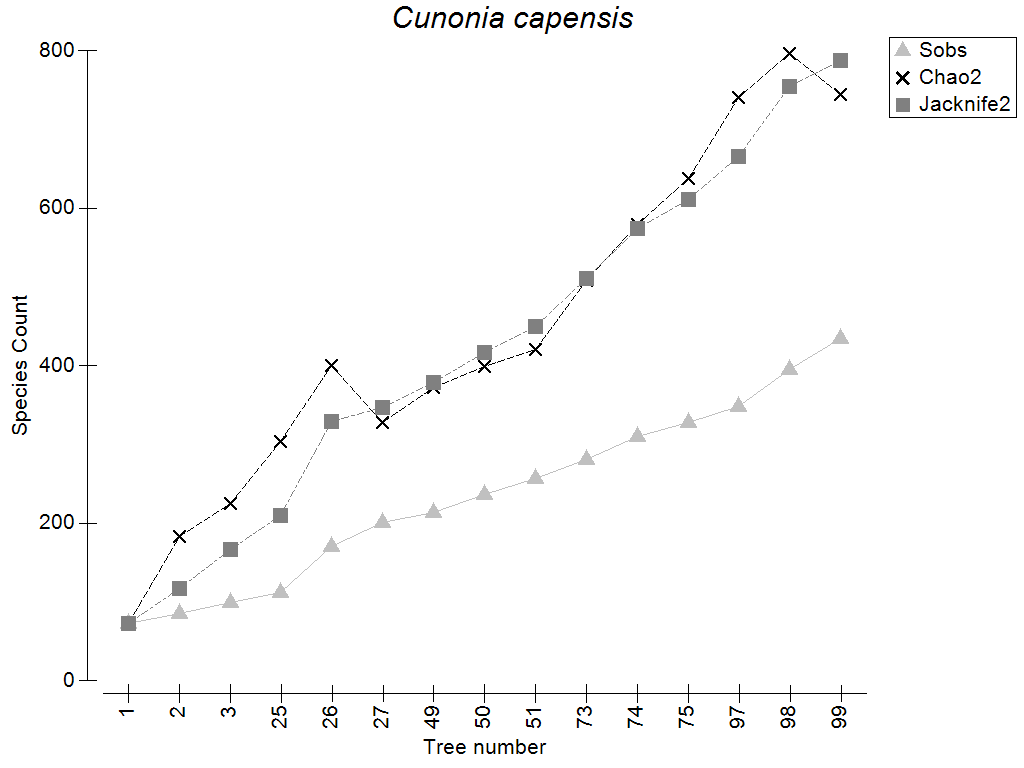


**Figure S1-2:** Species accumulation curves for overall arthropod richness sampled from 15 Cunonia capensis canopies featuring both Chao2 and Jacknife2 indices


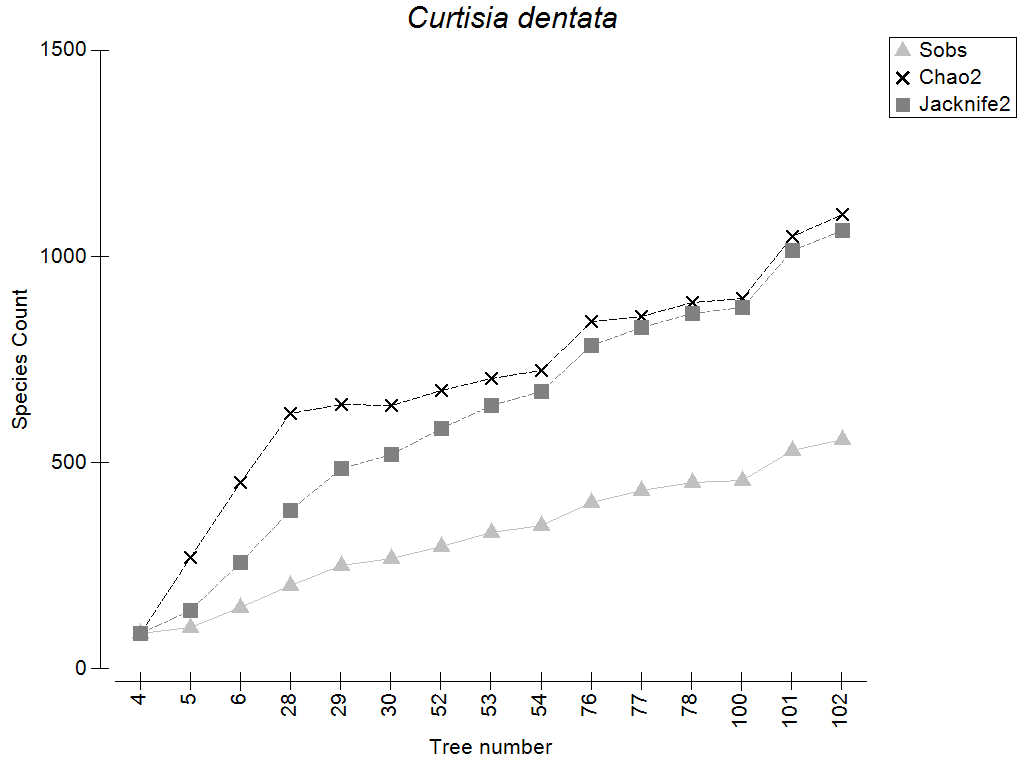


**Figure S1-3:** Species accumulation curves for overall arthropod richness sampled from 15 Curtisia dentata canopies featuring both Chao2 and Jacknife2 indices


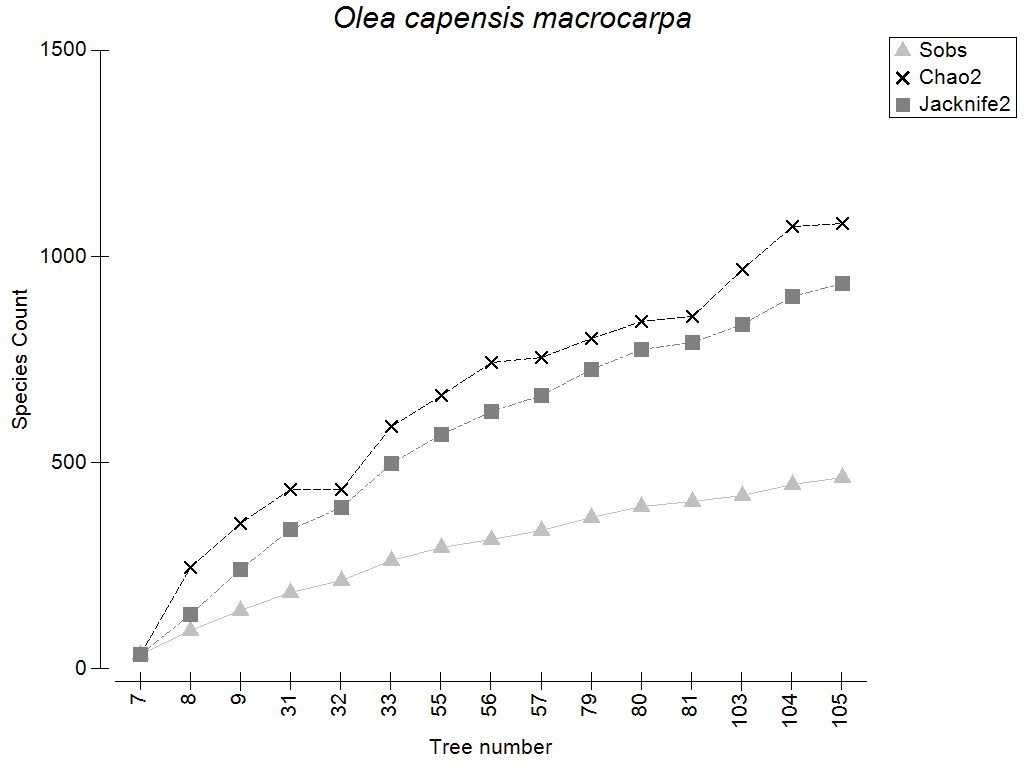


**Figure S1-4:** Species accumulation curves for overall arthropod richness sampled from 15 Olea capensis macrocarpa canopies featuring both Chao2 and Jacknife2 indices


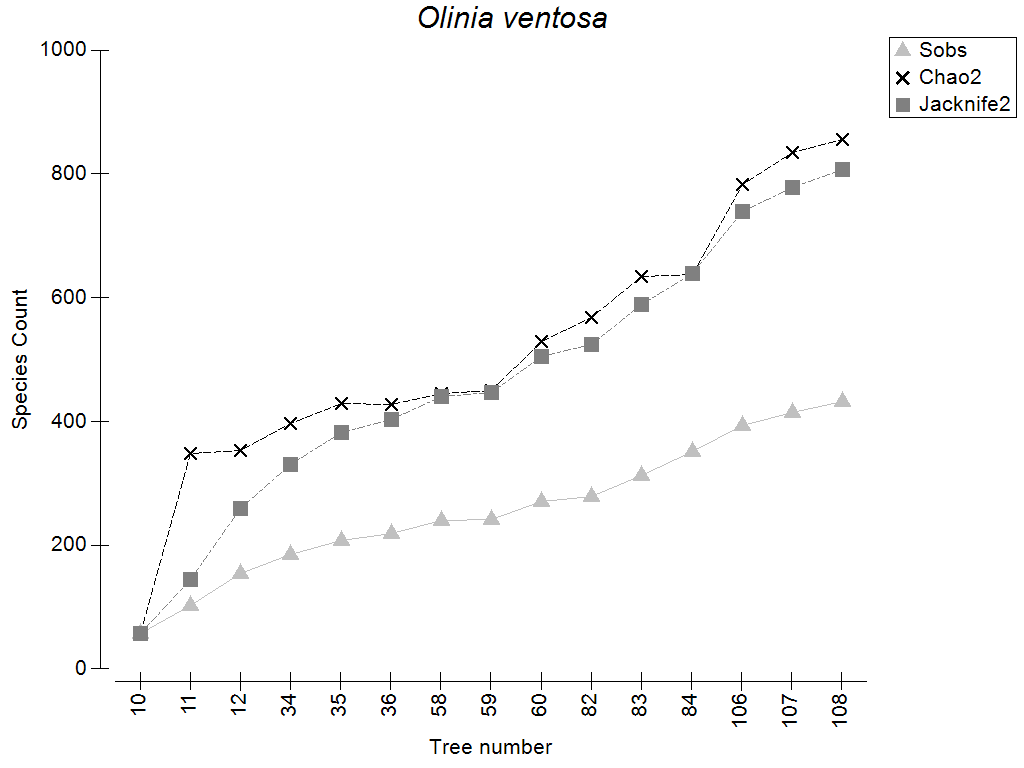


**Figure S1-5:** Species accumulation curves for overall arthropod richness sampled from 15 Olinia ventosa canopies featuring both Chao2 and Jacknife2 indices


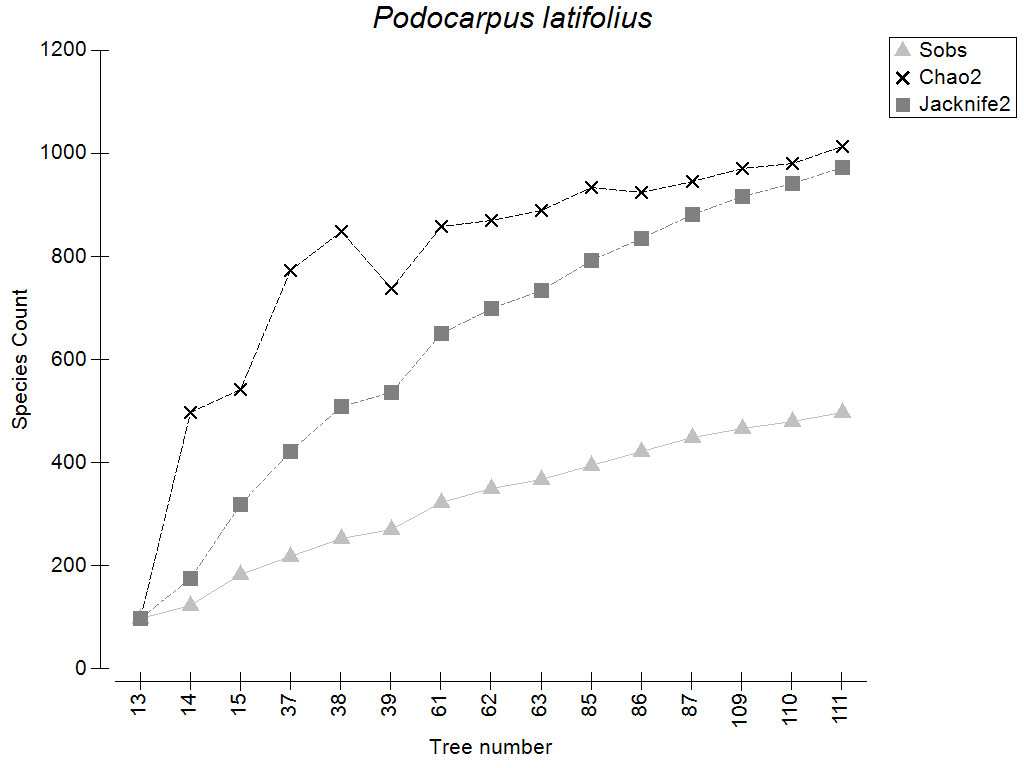


**Figure S1-6:** Species accumulation curves for overall arthropod richness sampled from 15 Podocarpus latifolius canopies featuring both Chao2 and Jacknife2 indices


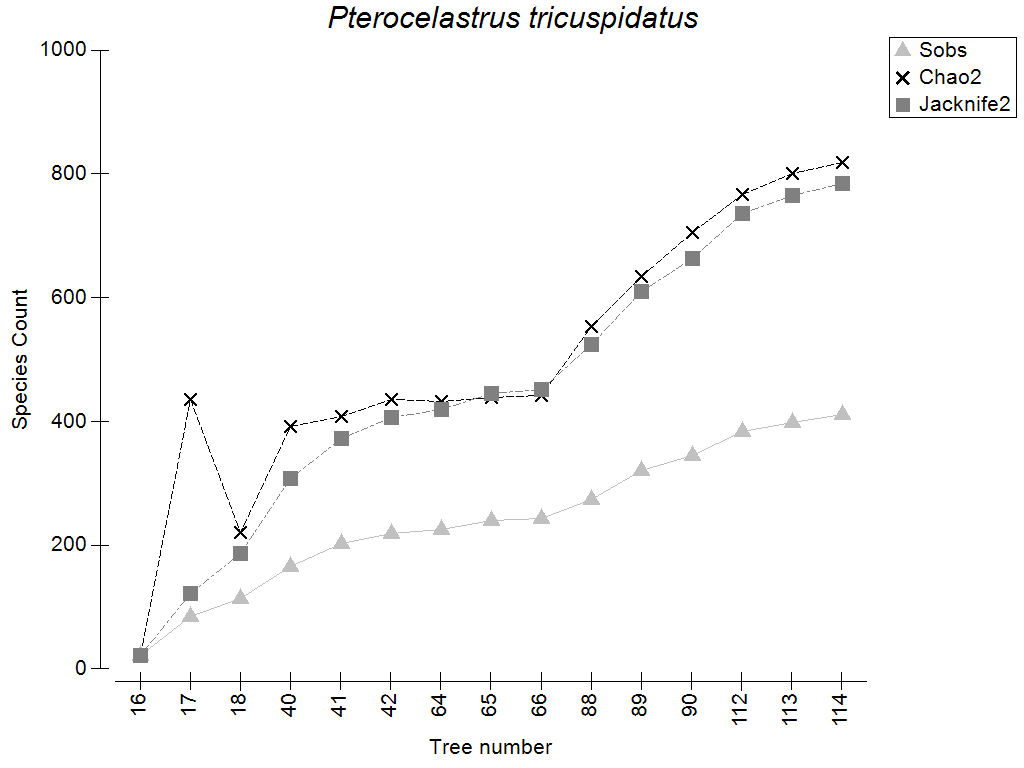


**Figure S1-7:** Species accumulation curves for overall arthropod richness sampled from 15 Pterocelastrus tricuspidatus canopies featuring both Chao2 and Jacknife2 indices


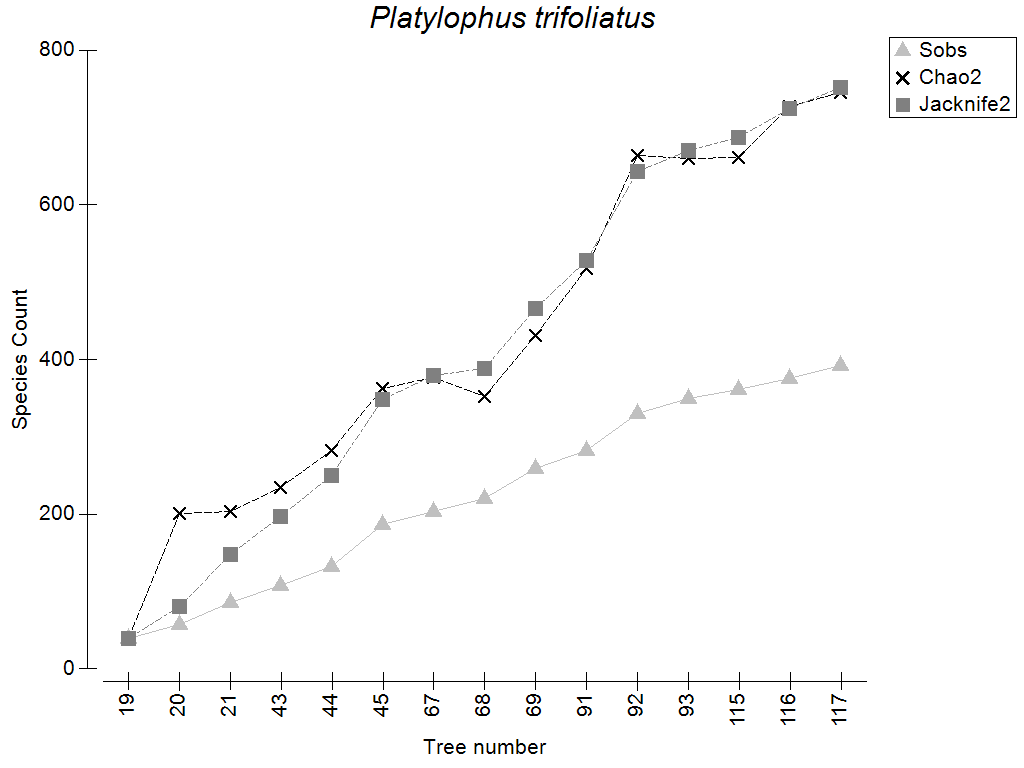


**Figure S1-8:** Species accumulation curves for overall arthropod richness sampled from 15 Platylophus trifoliatus canopies featuring both Chao2 and Jacknife2 indices


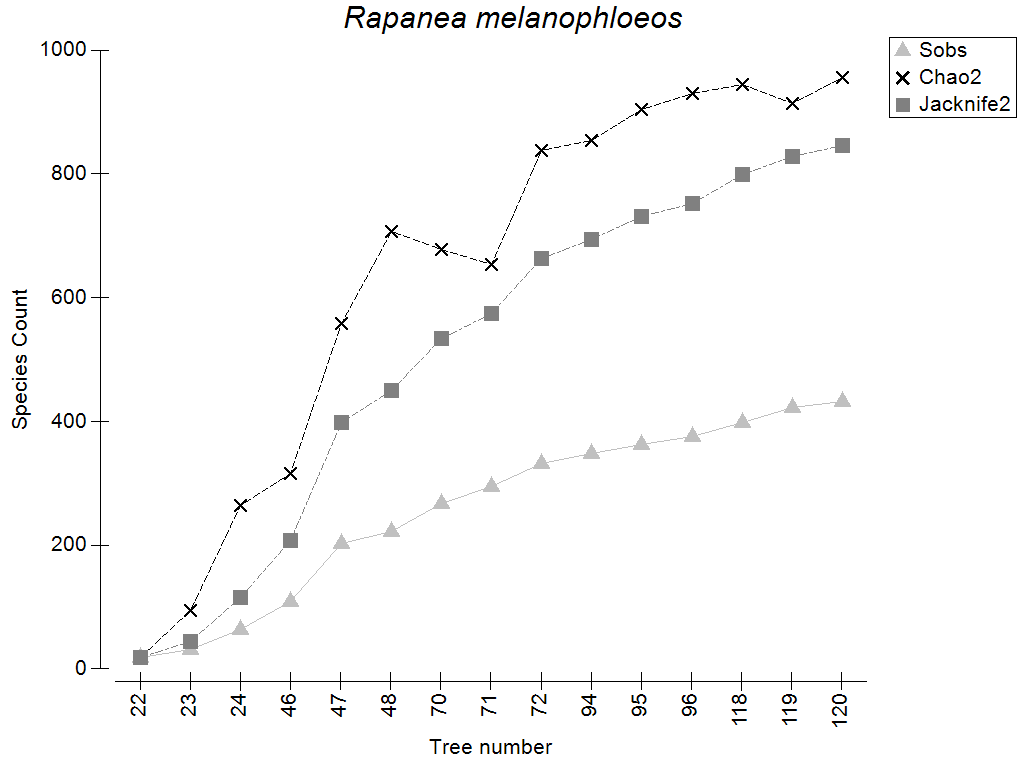


**Figure S1-9:** Species accumulation curves for overall arthropod richness sampled from 15 Rapanea melanophloeos canopies featuring both Chao2 and Jacknife2 indices


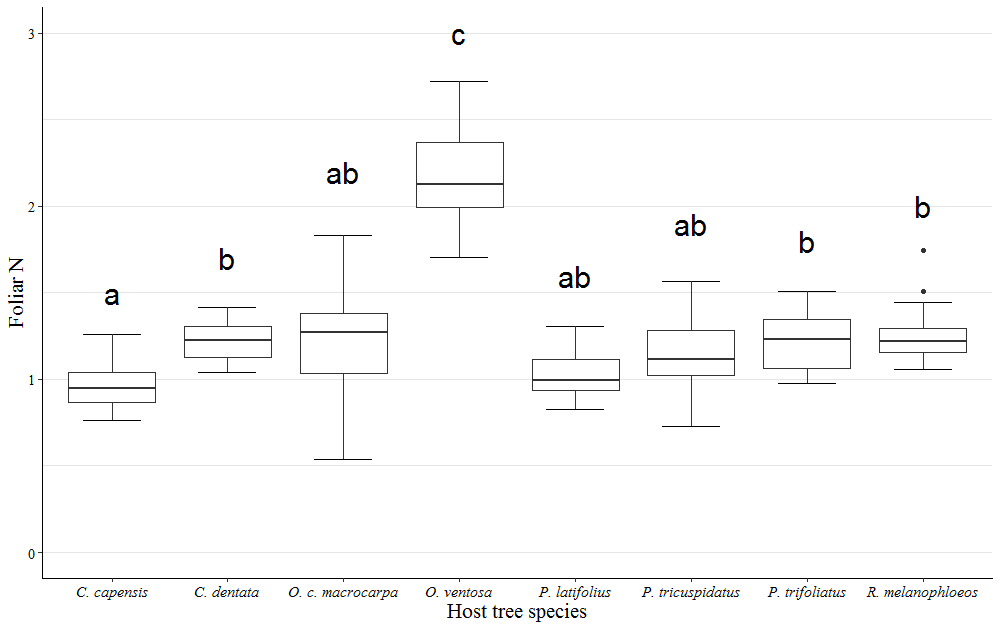


**Figure S2:** Box-and-whisker plots indicating interspecific differences in median of total foliar N determined by LMMs with shared letters above plots indicating similar medians based on a Tukey post-hoc test. Box indicates 25%-75% data range; whiskers indicate min (max(x), Q3 + 1.5*IQR) and max (min(x), Q1 – 1.5*IQR) outside the respective quartiles.


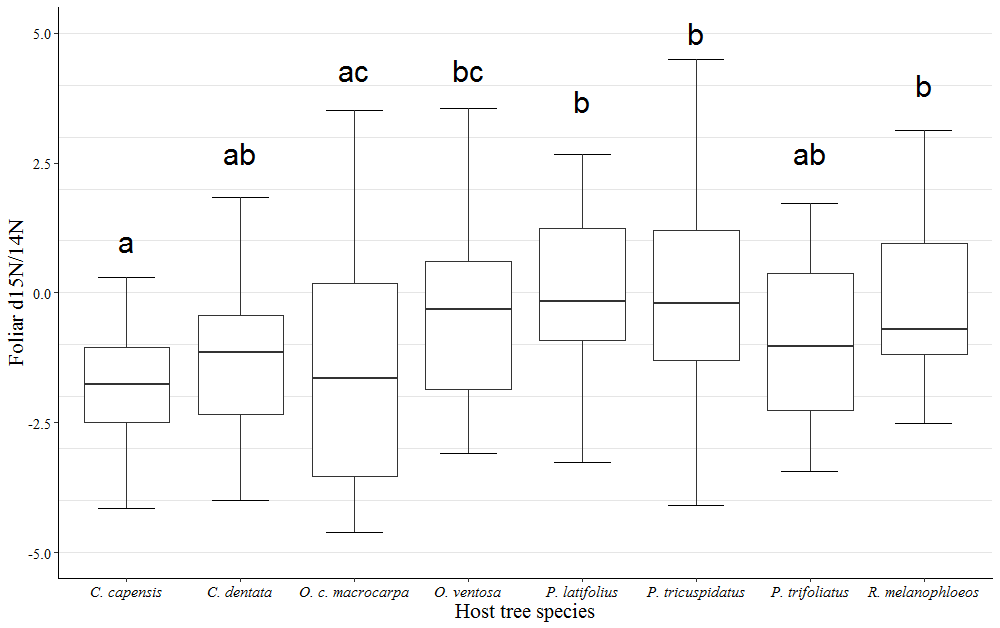


**Figure S3:** Box-and-whisker plots indicating interspecific differences in medians of foliar δ15N/14N determined by LMMs with shared letters above plots indicating similar medians based on a Tukey post-hoc test. Box indicates 25%-75% data range; whiskers indicate min (max(x), Q3 + 1.5*IQR) and max (min(x), Q1 – 1.5*IQR) outside the respective quartiles.


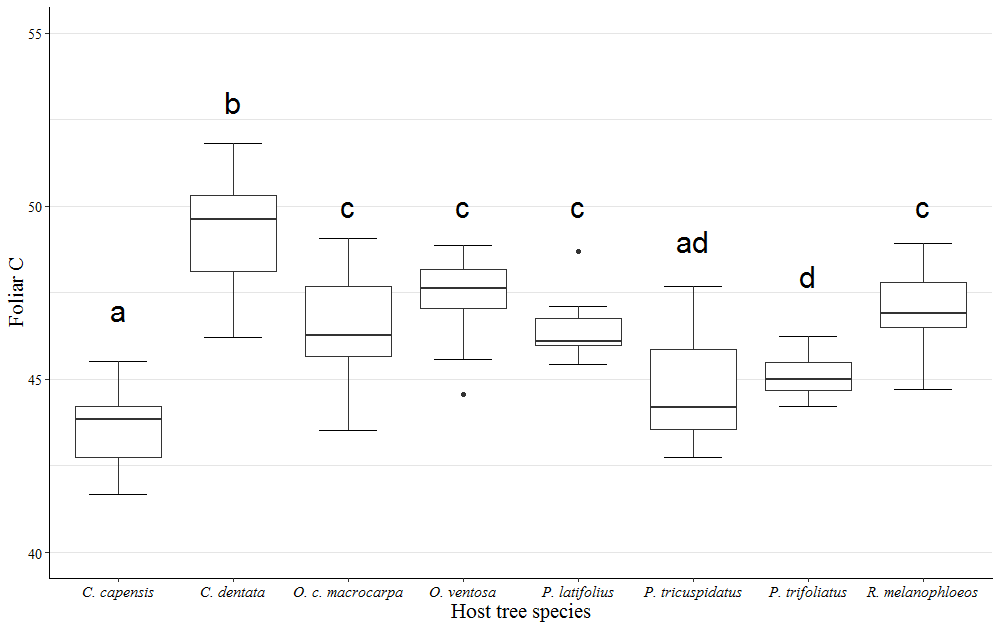


**Figure S4:** Box-and-whisker plots indicating interspecific differences in medians of total foliar C determined by LMMs with shared letters above plots indicating similar medians based on a Tukey post-hoc test. Box indicates 25%-75% data range; whiskers indicate min (max(x), Q3 + 1.5*IQR) and max (min(x), Q1 – 1.5*IQR) outside the respective quartiles.


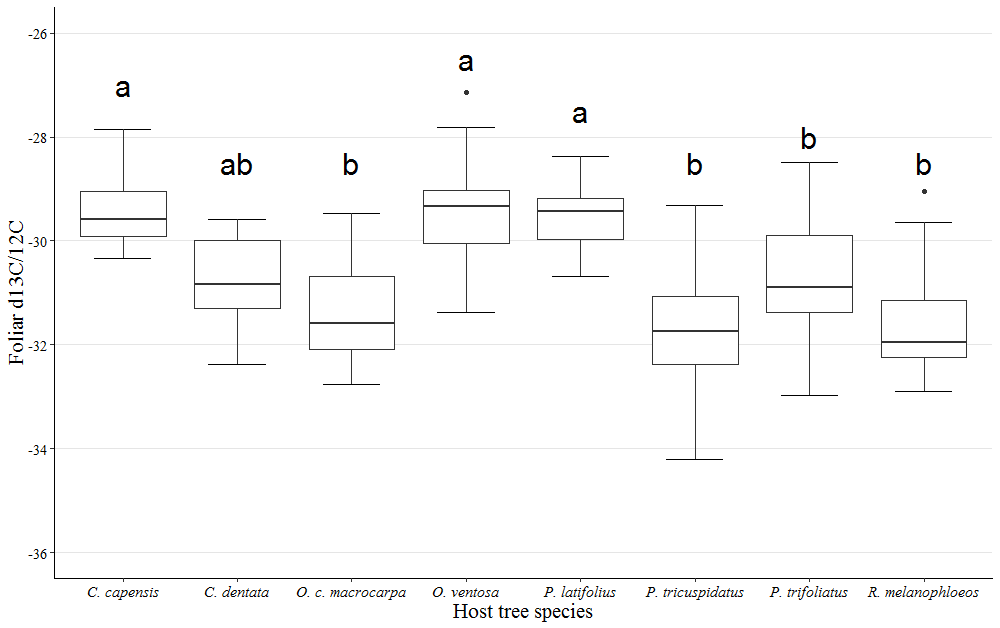


**Figure S5:** Box-and-whisker plots indicating interspecific differences in medians of foliar δ13C/12C determined by LMMs with shared letters above plots indicating similar medians based on a Tukey post-hoc test. Box indicates 25%-75% data range; whiskers indicate min (max(x), Q3 + 1.5*IQR) and max (min(x), Q1 – 1.5*IQR) outside the respective quartiles.


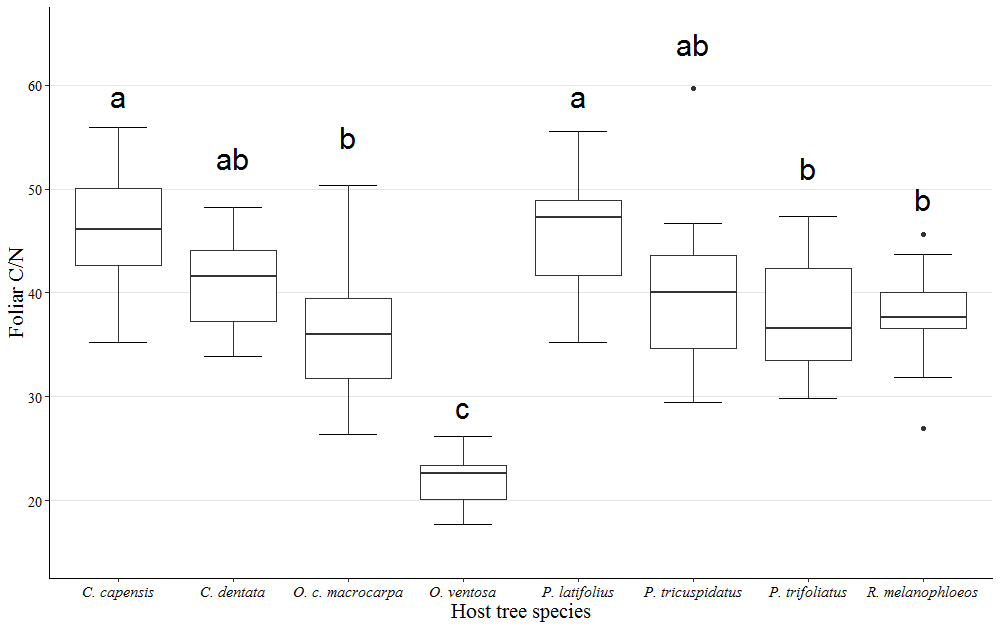


**Figure S6:** Box-and-whisker plots indicating interspecific differences in medians of foliar C/N determined by LMMs with shared letters above plots indicating similar medians based on a Tukey post-hoc test. Box indicates 25%-75% data range; whiskers indicate min (max(x), Q3 + 1.5*IQR) and max (min(x), Q1 – 1.5*IQR) outside the respective quartiles.


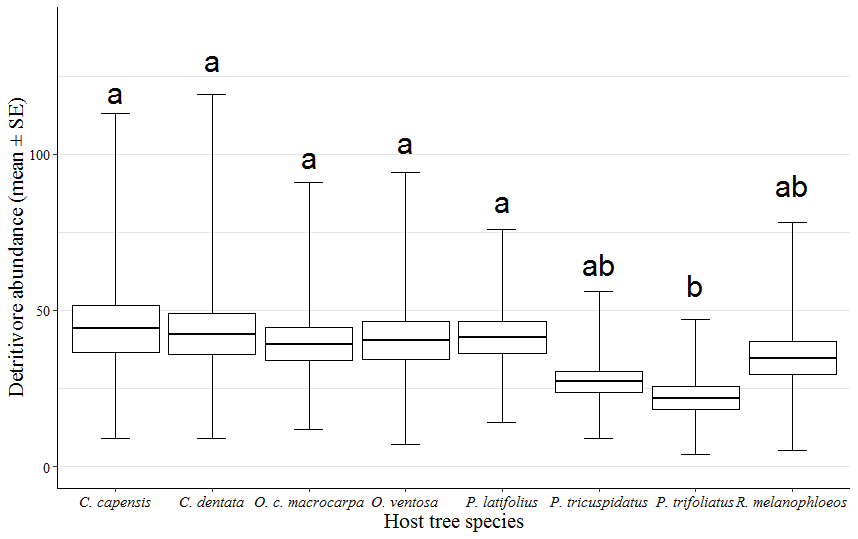


**Figure S7:** Box-and-whisker plots indicating interspecific differences in means of detritivore abundances determined by GLMMs with shared letters above plots indicating similar means. Boxes indicate standard error of the mean; whiskers indicate minimum and maximum values.


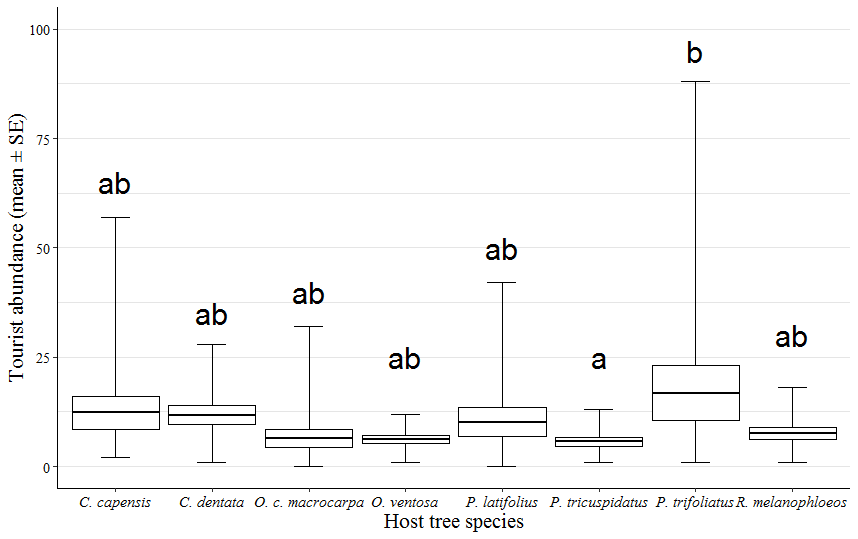


**Figure S8:** Box-and-whisker plots indicating interspecific differences in means of tourist abundances determined by GLMMs with shared letters above plots indicating similar means. Boxes indicate standard error of the mean; whiskers indicate minimum and maximum values.


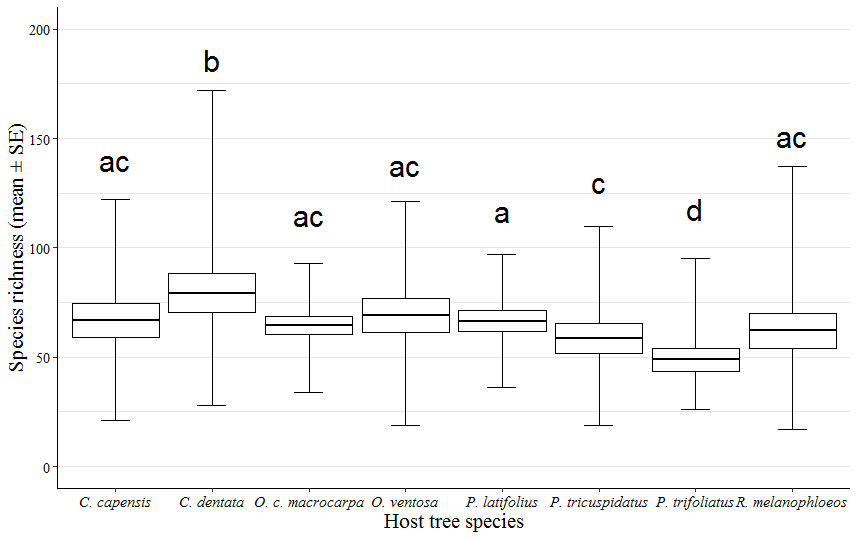


**Figure S9:** Box-and-whisker plots indicating interspecific differences in means of overall species richness determined by GLMMs with shared letters above plots indicating similar means. Boxes indicate standard error of the mean; whiskers indicate minimum and maximum values.

***
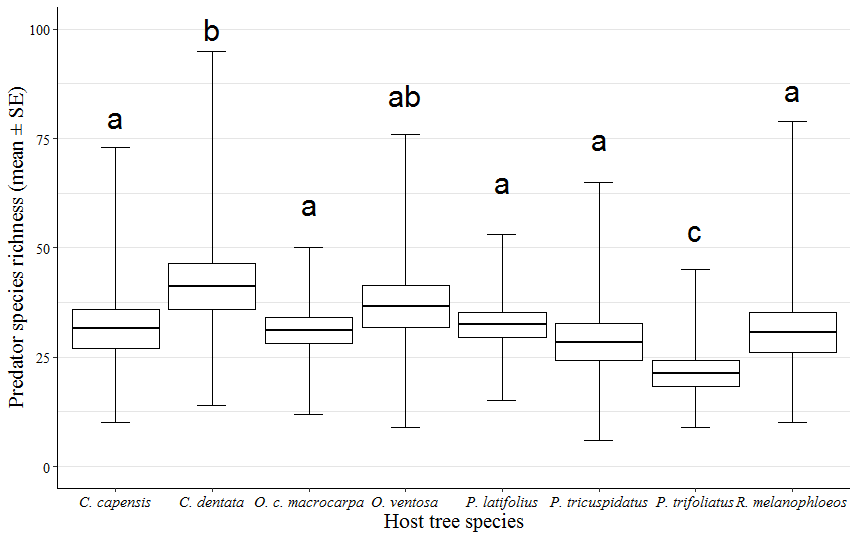
***

**Figure S10:** Box-and-whisker plots indicating interspecific differences in means of predator species richness determined by GLMMs with shared letters above plots indicating similar means. Boxes indicate standard error of the mean; whiskers indicate minimum and maximum values.


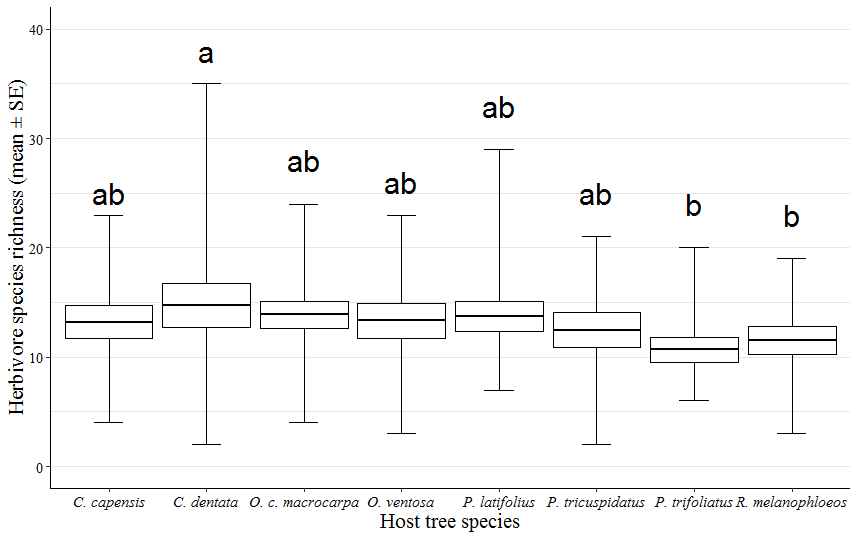


**Figure S11:** Box-and-whisker plots indicating interspecific differences in means of herbivore species richness determined by GLMMs with shared letters above plots indicating similar means. Boxes indicate standard error of the mean; whiskers indicate minimum and maximum values.


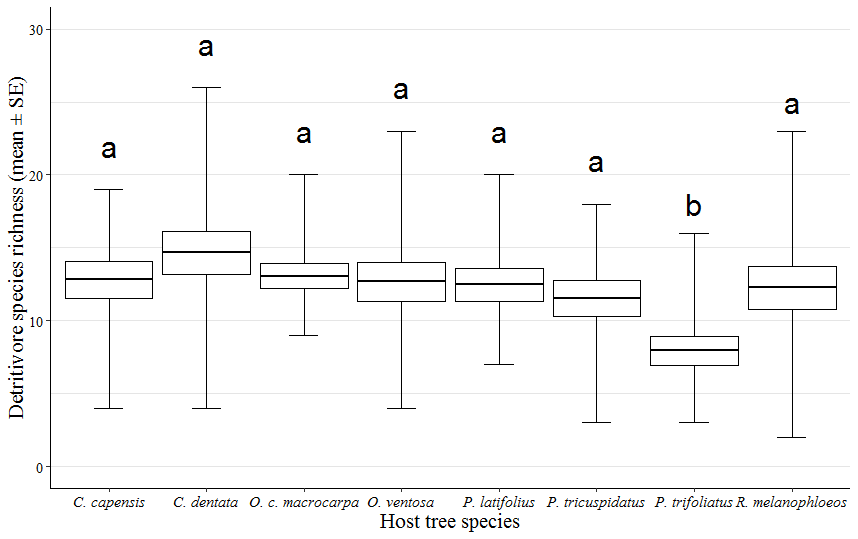


**Figure S12:** Box-and-whisker plots indicating interspecific differences in means of detritivore species richness determined by GLMMs with shared letters above plots indicating similar means. Boxes indicate standard error of the mean; whiskers indicate minimum and maximum values.

***
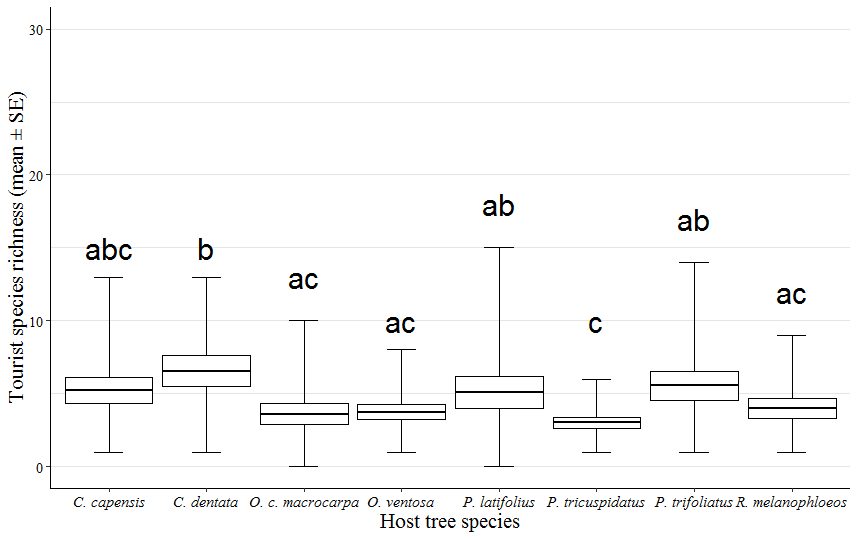
***

**Figure S13:** Box-and-whisker plots indicating interspecific differences in means of tourist species richness determined by GLMMs with shared letters above plots indicating similar means. Boxes indicate standard error of the mean; whiskers indicate minimum and maximum values.

**Table S2:** Results of the Permutational Multivariate Analysis of Variance indicating similarities in arthropod assemblage composition between selected tree species for each respective arthropod guild. Reported t-values for pairwise differences. Significance indicated in bold.

| Guild | Pseudo-F | Host species | *C. capensis* | *C. dentata* | *O. c. macrocarpa* | *O. ventosa* | *P. latifolius* | *P. tricuspidatus* | *P. trifoliatus* |
| --- | --- | --- | --- | --- | --- | --- | --- | --- | --- |
| Overall | **1.84**** | *C. dentata* | **1.22*** |  |  |  |  |  |  |
|  |  | *O. c. macrocarpa* | 1.01 | **1.27**** |  |  |  |  |  |
|  |  | *O. ventosa* | **1.22*** | **1.28**** | **1.29**** |  |  |  |  |
|  |  | *P. latifolius* | 1.15 | 1.14 | 1.16 | 1.02 |  |  |  |
|  |  | *P. tricuspidatus* | **1.25*** | **1.22*** | **1.35**** | **1.27*** | 1.16 |  |  |
|  |  | *P. trifoliatus* | 1.14 | **1.40***** | **1.21*** | **1.36**** | **1.17*** | **1.33**** |  |
|  |  | *R. melanophloeos* | 1.10 | **1.24*** | **1.21*** | **1.22*** | 1.09 | 0.99 | 1.13 |
| Herbivores | **2.01***** | *C. dentata* | 1.14 |  |  |  |  |  |  |
|  |  | *O. c. macrocarpa* | 0.96 | **1.50**** |  |  |  |  |  |
|  |  | *O. ventosa* | **1.44**** | **1.31*** | 1.21 |  |  |  |  |
|  |  | *P. latifolius* | **1.26*** | **1.30*** | **1.39*** | 0.97 |  |  |  |
|  |  | *P. tricuspidatus* | **1.40**** | 1.14 | **1.53**** | 1.15 | **1.31*** |  |  |
|  |  | *P. trifoliatus* | 1.10 | **1.33*** | **1.38*** | **1.44**** | 1.26 | **1.28*** |  |
|  |  | *R. melanophloeos* | 1.22 | 1.21 | **1.44*** | 1.18 | **1.32*** | 1.11 | 1.10 |
| Predators | **1.57***** | *C. dentata* | **1.35***** |  |  |  |  |  |  |
|  |  | *O. c. macrocarpa* | 1.02 | **1.20*** |  |  |  |  |  |
|  |  | *O. ventosa* | 1.06 | **1.27**** | **1.36***** |  |  |  |  |
|  |  | *P. latifolius* | 1.11 | 1.10 | 1.15 | 1.00 |  |  |  |
|  |  | *P. tricuspidatus* | **1.22*** | 1.12 | 1.14 | **1.23*** | 1.06 |  |  |
|  |  | *P. trifoliatus* | 1.00 | **1.37***** | 1.06 | **1.29**** | 0.96 | **1.17*** |  |
|  |  | *R. melanophloeos* | 0.94 | **1.27**** | 1.09 | **1.19*** | 0.98 | 0.90 | 0.97 |
| Detritivores | **2.23***** | *C. dentata* | 1.05 |  |  |  |  |  |  |
|  |  | *O. c. macrocarpa* | 1.03 | 1.22 |  |  |  |  |  |
|  |  | *O. ventosa* | 1.24 | **1.39*** | 1.23 |  |  |  |  |
|  |  | *P. latifolius* | 1.11 | 1.06 | 1.08 | 1.16 |  |  |  |
|  |  | *P. tricuspidatus* | 1.21 | **1.41*** | **1.40*** | 1.20 | 1.16 |  |  |
|  |  | *P. trifoliatus* | 1.08 | **1.51**** | **1.39*** | **1.65***** | **1.36*** | **1.38*** |  |
|  |  | *R. melanophloeos* | 1.04 | 1.29 | 1.18 | 1.16 | 1.12 | 1.00 | 1.21 |
| Tourists | **1.67***** | *C. dentata* | 1.18 |  |  |  |  |  |  |
|  |  | *O. c. macrocarpa* | 0.95 | 1.11 |  |  |  |  |  |
|  |  | *O. ventosa* | 1.23 | 1.30 | 0.99 |  |  |  |  |
|  |  | *P. latifolius* | **1.47**** | 1.18 | 1.04 | 0.95 |  |  |  |
|  |  | *P. tricuspidatus* | 1.07 | **1.37*** | 1.22 | **1.45*** | 1.18 |  |  |
|  |  | *P. trifoliatus* | 1.30 | **1.39*** | 0.74 | 0.86 | 1.02 | **1.39*** |  |
|  |  | *R. melanophloeos* | **1.44*** | 1.23 | 1.24 | 1.28 | 1.14 | 1.13 | 1.28 |
| Ants | 1.22 |  |  |  |  |  |  |  |  |

‘*’ P ˂ 0.05, ‘**’P ˂ 0.01, ‘***’P ˂ 0.001.


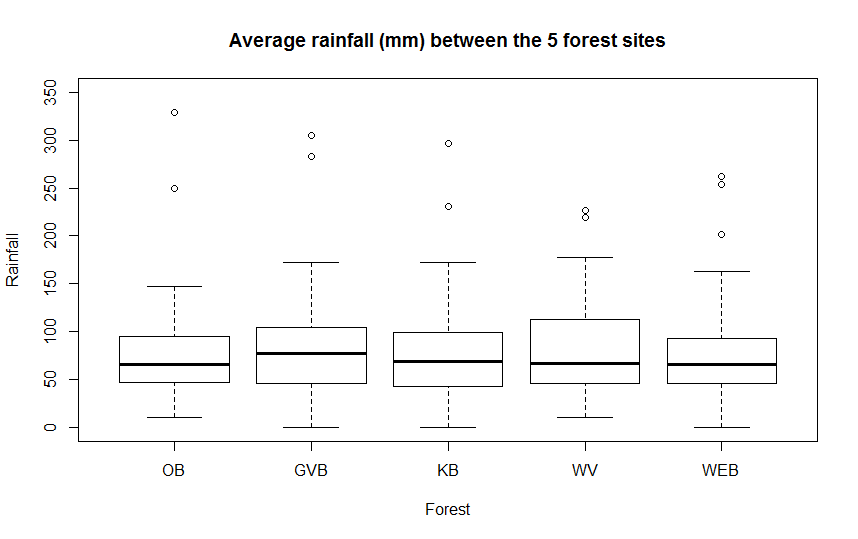


**Figure S14**: Box-and-whisker plots indicating mean monthly rainfall (mm) between the five forest sites during the five years (2012-2016) leading up to the study. Box indicates 25%-75% data range; whiskers indicate min (max(x), Q3 + 1.5*IQR) and max (min(x), Q1 – 1.5*IQR) outside the respective quartiles.OB = Oubos, GVB = Grootvadersbosch, KB = Kleinbos, WV = Woodville, WEB = Witelsbos.

**Table S3:** List of the 8 sampled tree species including their higher taxonomic classification and relative abundance

| **Order** | **Family** | **Host species** | **Relative abundance*** |
| --- | --- | --- | --- |
| Oxidales | Cunoniaceae | *Cunonia capensis* | 4.88% |
| Cornales | Curtisiaceae | *Curtisia dentata* | 4.38% |
| Lamiales | Oleaceae | *Olea capensis macrocarpa* | 12.15% |
| Myrtales | Penaeaceae | *Olinia ventosa* | 6.77% |
| Pinales | Podocarpaceae | *Podocarpus latifolius* | 9.36% |
| Celastrales | Celastraceae | *Pterocelastrus tricuspidatus* | 8.17% |
| Oxidales | Cunoniaceae | *Platylophus trifoliatus* | 4.58% |
| Ericales | Myrsinaceae | *Rapanea melanophloeos* | 14.04% |

*Determined from plot data (plot size = 200 m², n = 24 plots per forest, n = 120 plots in total) as a percentage of the number of individuals (n=1004) of all of the 40 tree species found in the plots across the 5 forest sites as described in the ‘Plant characteristics’ section.
